# Motifs of brain cortical folding from birth to adulthood

**DOI:** 10.1038/s41467-026-77314-5

**Published:** 2026-09-03

**Authors:** Yourong Guo, Mohamed A. Suliman, Logan Z. J. Williams, Renato Besenczi, Kaili Liang, Simon Dahan, Vanessa Kyriakopoulou, Grainne M. McAlonan, Alexander Hammers, Jonathan O’Muircheartaigh, Emma C. Robinson

**Affiliations:** 1 Research Department of Biomedical Computing, School of Biomedical Engineering and Imaging Sciences, King’s College London, London, UK; 2 Research Department of Early Life Imaging, School of Biomedical Engineering and Imaging Sciences, King’s College London, London, UK; 3 Department of Child and Adolescent Psychiatry, Institute of Psychiatry, Psychology and Neuroscience, King’s College London, London, UK; 4 Department of Forensic and Neurodevelopmental Sciences, Institute of Psychiatry, Psychology and Neuroscience, King’s College London, London, UK; 5 NIHR Biomedical Research Centre: Maudsley, South London and Maudsley NHS Foundation Trust and King’s College London, London, UK; 6 King’s College London and Guy’s and St. Thomas’ PET Centre, King’s College London, London, UK; 7 MRC Centre for Neurodevelopmental Disorders, King’s College London, London, UK

**Keywords:** Computational neuroscience, Brain, Image processing

## Abstract

Cortical folding of the brain is widely regarded as an interplay between genetic programming and biomechanical forces, closely linked to cytoarchitectonic regionalisation. Abnormal folding patterns are frequently observed in neurodevelopmental conditions and psychiatric disorders. However, significant inter-individual variability of secondary and tertiary folds obscures the detection of shape biomarkers and confounds the investigation of folding-functional relationships. Here, we investigate cortical folding heterogeneity at a fine scale, using Multimodal Surface Matching with Hierarchical Templates (MSM-HT), a hierarchical surface registration, to parse cortical folding patterns into a representative family of distinct anatomical templates. By applying this technique both to young adults from the Human Connectome Project (HCP) and neonates in the Developing HCP and Brain Imaging in Babies (BIBS) cohorts, we identify and characterise common lobe-wise folding patterns: observing consistency across both age groups, with neonatal samples showing less variation. Crucially, we highlight significant hemispheric asymmetry within the temporal lobe in adults, with a consistent trend in neonates. This study provides a critical step towards understanding brain asymmetry and complex relationships between folding and function, offering a robust framework to generalise the uncovered cortical folding motifs across datasets and developmental stages.

## Abstract (web summary)

Guo and colleagues parse cortical folding variability into a family of distinct anatomical motifs that generalise across adults and neonates. These motifs shed light on structural asymmetry, heritability, and folding-function links.

## Introduction

The human cerebral cortex, with its intricate patterns of sulci and gyri, achieves its remarkable surface area expansion through folding, enabling more complex brain functions. A delicate interplay of genetic factors and biomechanical forces drives this process. Starting at around 11–16 weeks, cortical progenitor cells initially establish a ‘protomap’^1^; these early cytoarchitectonic areas are genetically defined, largely stable through development, and are closely linked to primary cortical functions.

As cortical development progresses, regional variations in growth, driven by different types of neuronal progenitor cells, along with biomechanical forces, lead to cortical folding^2,3^. Primary folds, which emerge around 20 weeks of gestation, are predominantly shaped by genetic influences, resulting in conserved structures across individuals. Unlike primary folds, secondary and tertiary folds, mostly shaped by week 38, are influenced significantly by intrauterine environmental factors^4^ and biomechanical forces, leading to substantial variability and asymmetry. Even with genetically identical individuals (monozygotic twins), significant variability in folding patterns has been observed, highlighting the highly dynamic biomechanical process^5^.

It has been challenging to characterise the individual variability of cortical folding. In the absence of big open-data collections and without access to advanced image processing tools, early work was necessarily observational and performed on small samples of brains. For instance, Ono et al.^6^ performed a careful examination of 25 brains postmortem, resulting in a detailed description of the variabilities of length, branching, connections, and interruptions of major sulci throughout the entire brain. Such observational studies are highly time-intensive and dependent on neuroanatomical expertise, but do offer detailed characterisation of whole-brain cortical shape^7^. More recent data-driven, automated methods^8–14^ offer less labour-intensive alternatives; but so far have been methodologically constrained to independent investigation of a subset of sulci, limiting scope for exploratory analyses into the extent to which cortical folding patterns co-vary within or between hemispheres, or align with cortical areas.

Though it is often assumed that morphology and function are tightly coupled, this relationship may not hold across the entire brain. While primary cortical folds tend to align with cytoarchitectonic areas supporting sensory/motor functions^15–18^, evidence suggests that this relationship is less straightforward in regions supporting complex abilities^19,20^. As pointed out very early by Brodmann, folding landmarks do not correspond to cytoarchitectonic borders precisely^21^. For instance, the presence or absence of paracingulate sulci does not affect the existence of its associated cytoarchitectonic areas (area 32)^22^.

From a more global perspective of brain function, neuropsychiatric and neurodevelopmental disorders have been associated with atypical cortical shapes (both regionally and across the whole brain)^23–28^. However, associating shape with more subtle phenotypes or genotypes has been challenged by marked typical heterogeneity of folding across the population. There is a clear need for analytic approaches that consider this variability to provide a better baseline of what “typical" can be^29^. A better analytical framework that addresses heterogeneity in normal anatomy could address the problem of inconsistent and variable findings for these disorders^30,31^.

Disentangling natural cortical shape variability from clinically or cognitively salient features starts with improved characterisation of the scale of cortical shape variability across large populations. To address this, here we propose a hierarchical surface-registration approach (Multimodal Surface Matching with Hierarchical Templates, MSM-HT) that clusters—then aligns—individual hemispheres into groups that share patterns of cortical folding; resulting in a family of sharp templates, which collectively characterise the most commonly expressed cortical shape motifs, within each of the frontal, temporal and parieto-occipital lobes. These folding motifs strongly resemble those described in post-mortem studies^6^, while benefiting from the scale afforded by big-data analyses to support investigation into the prevalence and asymmetry of motifs. Moreover, propagation of spatial mappings to functional imaging data critically supports investigation into the co-localisation of cortical areas and folds.

We validate our findings in both 1110 young adults, from the Human Connectome Project (HCP), and in 781 term-age neonates from the developing HCP (dHCP) and the Brain Imaging in Babies (BIBS) studies. We reveal consistent yet evolving development of cortical folding from neonates to young adults. At term and adult ages, we found the temporal lobe motifs to be asymmetric, indicating that this morphological asymmetry is already present at birth. Importantly, the folding-function relationship was found not to be tightly correlated in secondary and tertiary-order folds. Altogether, these findings provide a unique perspective on how genetic, structural, and functional processes integrate to shape cortical organisation.

## Results

A key assumption underlying human neuroimaging studies is that spatial normalisation can map individual brains to a single population-average space, in which common structures (e.g., sulci and gyri) overlap; this then allows for straightforward comparison and modelling of these features during downstream analyses. Since optimisation of these mappings is ill-posed, image registration algorithms are typically regularised with diffeomorphic constraints, which enforce smooth, invertible transforms that allow no folding or tearing of the deformation grid. This contradicts the body of evidence, described above, which shows that equivalent sulci can appear continuous in some individuals, but branched or split in others. As a result, enforcing globally consistent correspondence may obscure, rather than capture, the true diversity of folding patterns. To address this, previous approaches have employed strategies such as multi-atlas segmentation^32^, or group-wise registration^33,34^, in which a fixed number of templates may be defined to capture shape variability; however, these have typically been formulated in the volumetric domain, where diffeomorphic regularisation is applied in Euclidean space, and therefore do not fully respect the intrinsic geometry of the cortical sheet. Consequently, they may be insufficient to capture the scale and structured variability of cortical folding across individuals.

In this paper, we explore a hierarchical image registration framework (MSM-HT), which leverages learning-based surface-registration methods^35^ to perform fast diffeomorphic alignment between all pairs of hemispheres, from a large cohort. We then assess similarity between cortical folding patterns by how easily they can be diffeomorphically aligned; this then allows common folding patterns to be clustered and co-aligned using multimodal surface matching (MSM)^36–38^ (see Hierarchical MSM registration (MSM-HT)).

### Characterising modes of cortical shape variation and their frequency

**Fig. 1:**
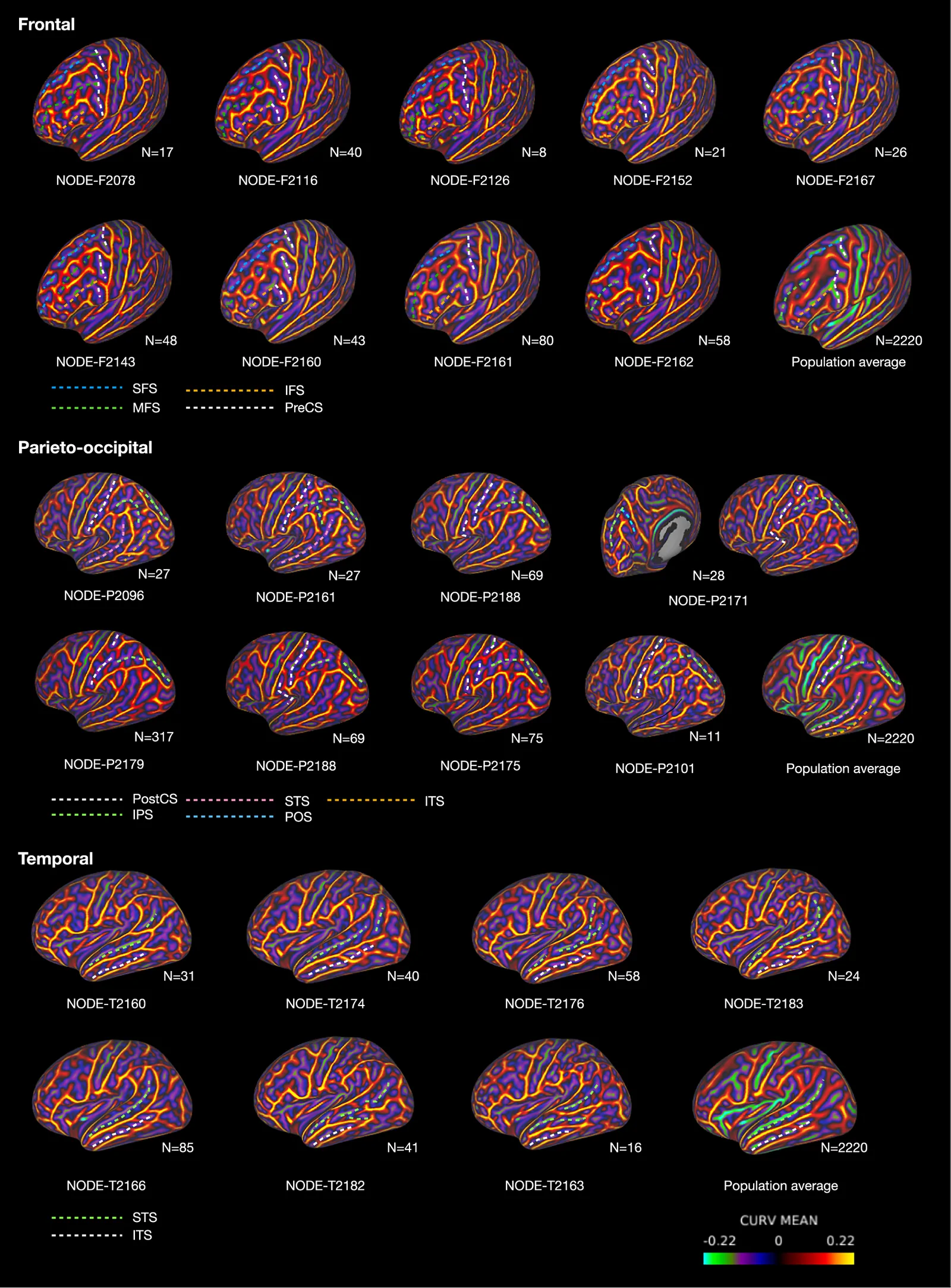
Distinctive common folding motifs identified using MSM-HT. Cortical folding motifs are visualised in curvature, where larger values correspond to gyral crowns. Top: representative folding motifs in the frontal lobe, illustrating variability in the superior Frontal sulcus (SFS), inferior frontal sulcus (IFS), middle frontal sulcus (MFS), and precentral sulcus (PreCS). Uninterrupted PreCS are rare (e.g., cluster F2126), whereas segmentation into two or three segments is more common (e.g., clusters F2078 and F2160). The PreCS is typically connected to the SFS, with occasional exceptions (e.g., cluster F2161, *N* = 80). The MFS shows the greatest variability, forming up to three or four segments (e.g., cluster F2143), and can link the IFS, SFS, and PreCS. Middle: Representative folding motifs in the parieto-occipital lobe. The postcentral sulcus (PostCS) appears either uninterrupted (17/30 clusters; e.g., cluster P2096) or divided into two segments (12/30 clusters; e.g., clusters P2188 and P2101), whereas three-segment configurations are rare (1/30 clusters, ~3.4% of hemispheres; cluster P2175), in which the middle segment connects to the intraparietal sulcus (IPS). In eight clusters, the PostCS connects to the Sylvian fissure (e.g., cluster P2171). The IPS forms between one and three segments and may exhibit ascending (e.g., cluster P2188) or descending side branches (e.g., clusters P2096, P2161). These branches can extend towards neighbouring structures, including the inferior temporal sulcus (e.g., cluster P2096) or superior temporal sulcus (STS; cluster P2161), and may also connect with parietal-occipital sulci (POS; Cluster P2171). Bottom: Representative folding motifs in the temporal lobe. The STS and ITS exhibit between one and four segments, with the STS showing more branching patterns and the ITS displaying more regular, ladder-like segments (e.g., cluster T2174). In line with Ono’s Atlas, the STS most commonly extends into the angular sulcus (77.5% of cases; e.g., cluster T2166), whereas, less frequently, it shows a double ending towards both angular and anterior occipital sulci (in 28.1% of cases; e.g., cluster T2176), or extends further into the occipital lobe (e.g., cluster T2169; *N* = 31). Branched STS segments may connect with the ITS (e.g., clusters T2183, T2182, T2163 and T2176), and rare patterns include connections with the Sylvian fissure (e.g., cluster T2183; *N* = 24, ~1.1% of the cases).

Thirty templates were generated separately for each of the frontal, parieto-occipital, and temporal lobes based on HCP young adult (HCP-YA) data (Fig. 1 and Supplementary Figs. S1–S3). These motifs show close agreement with the descriptions of Ono’s atlas^6^, particularly in terms of segment variations and branching patterns.

Focusing first on the frontal lobe, the most common pattern comprises a precentral sulcus (PreCS) divided into two segments by the middle frontal gyrus (MFG), a superior frontal sulcus (SFS) divided into two segments, and an inferior frontal sulcus (IFS) divided into one or two segments, with the posterior end of the IFS connecting to the inferior segment of the PreCS. More broadly, the PreCS often forms two or three segments, whereas uninterrupted PreCS (as seen in cluster F2126) are rare, occurring in only eight hemispheres and not described in Ono’s atlas. The PreCS is also usually connected to the SFS, with only occasional exceptions. Among frontal sulci, the middle frontal sulcus (MFS, termed intermediate frontal sulcus in^6^) showed the greatest variability, ranging up to three or four segments in some clusters and sometimes forming uncommon connections, with the anterior segment of IFS or linking the SFS, IFS and PreCS.

In the parieto-occipital lobe, the most common cortical folding pattern comprises one or two segments of postcentral sulcus (PostCS) and a segmented intraparietal sulcus (IPS), with the anterior end of the IPS typically connecting to the PostCS, and the posterior end extending towards the occipital lobe. In general, the PostCS is either continuous or divided into two segments, whereas more complex segmentations are rare. The IPS shows greater variability, forming between one and three segments and frequently giving rise to ascending or descending side branches. In some cases, these branches extend towards the temporal or occipital structures, including connections with the inferior or superior temporal sulci. Occasional variants also include direct connections between the PostCS and the Sylvian fissure or between IPS branches and parietal-occipital sulci.

In the temporal lobe, the most common folding pattern comprises one or two segments of the superior temporal sulcus (STS) and three or four segments of the inferior temporal sulcus (ITS), with the anterior end of STS located at the temporal pole and its posterior end extending towards the angular sulcus (AS). In general, both STS and ITS show segmentation variability, with STS exhibiting more complex branching patterns, whereas the ITS tends to form more ‘ladder-like’ segments. In line with Ono’s Atlas, the STS mostly extends into the AS, but it occasionally displays a double ending or extends further into the occipital lobe. In some cases, branched STS segments connect with the ITS. While rarer configurations include connections with the Sylvian fissure.

We further characterised each folding motif in terms of curvature, sulcal depth, surface area, and asymmetry (Supplementary Table S4). We then examined cross-lobar consistency and found 2 significant co-occurrences reaching high effect size (both linking frontal and parieto-occipital motifs), with cross-lobar agreement otherwise weak (see Supplementary Results S2.1 and Supplementary Fig. S4 for additional analyses).

### Genetic influences on cortical folding motifs

Heritability provides insights into the genetic mechanisms underpinning cortical folding. Although early-formed primary folds are known to be heritable, substantial variability can still be observed even in monozygotic (MZ) twins. To quantify the genetic contribution to cortical folding motifs, we modelled similarity-to-motif measures using bivariate ACE (additive genetic, shared environmental, and unique environmental) models, retaining AE models where shared environmental effects did not improve model fit. Across lobes, heritability estimates were moderate for both hemispheres (mean ± SE across motifs; frontal: left 0.48 ± 0.05, right 0.33 ± 0.03; parieto-occipital: left 0.35 ± 0.09, right 0.42 ± 0.05; temporal: left 0.50 ± 0.03, right 0.44 ± 0.04), with strong genetic correlations between hemispheres in the frontal lobe (${r}_{g}^{LR}\approx 1$), high in the parieto-occipital lobe (0.778–0.999), and moderate in the temporal lobe (0.434–0.719), indicating largely shared genetic influences rather than independent hemispheric effects (full model estimates are reported in Supplementary Table S1). Consistent with these findings, a motif-level co-occurrence analysis showed that MZ twins were more likely than dizygotic (DZ) twins to share the same folding motifs across all lobes (see Supplementary Results S2.2).

### Greater asymmetry of the temporal lobe

**Fig. 2:**
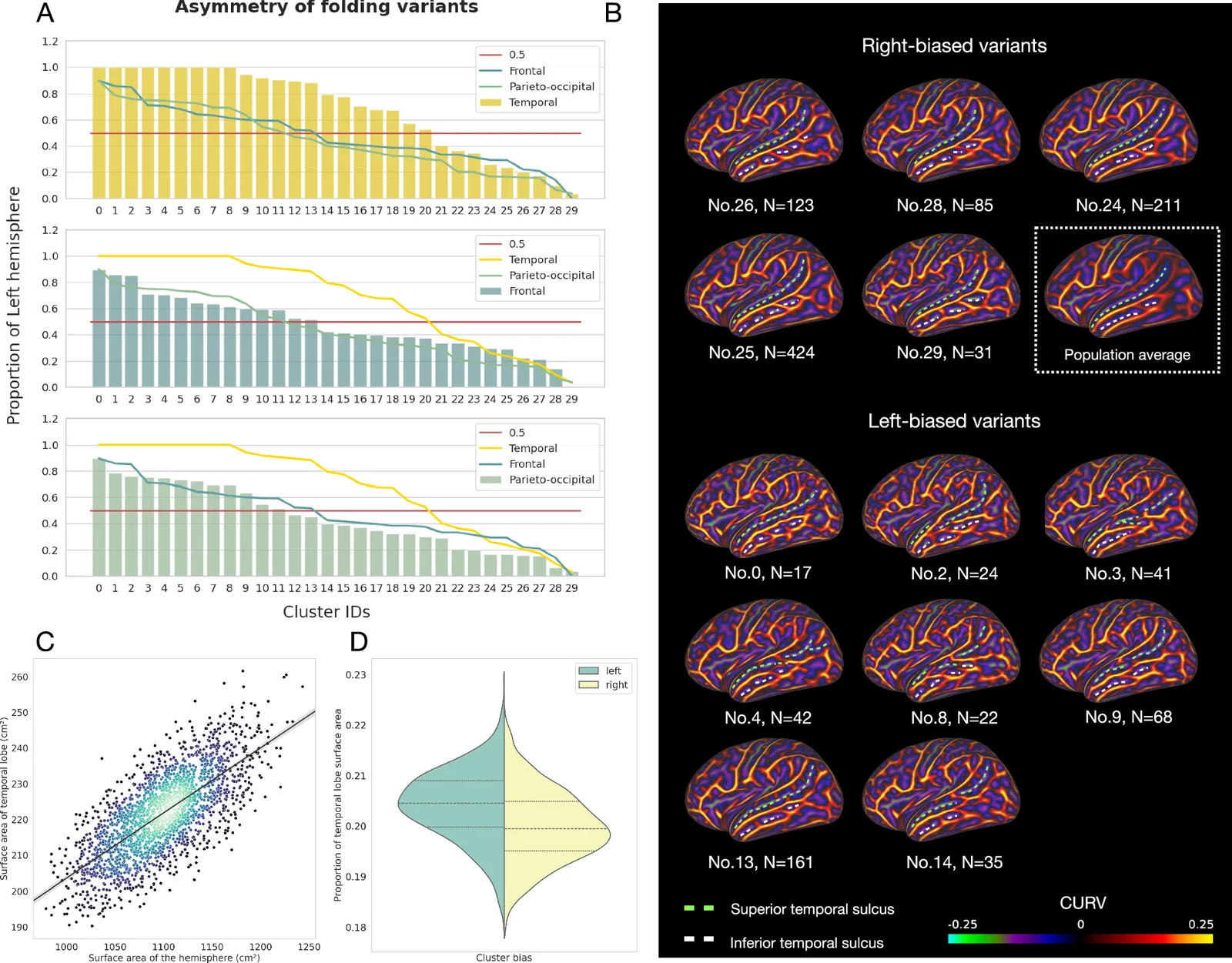
Asymmetry of the temporal lobe cortical folding patterns. **A** Proportions of the left hemispheres used to construct each template are sorted in descending order. There is significantly greater asymmetry of the temporal lobe relative to the frontal (two-sided Mann-Whitney U test, *n* = 30 templates per lobe, *U* = 626, FDR-corrected *P* = 0.014, median shift = 0.26, 95% CI [0.07, 0.39]) and parieto-occipital regions (*U *= 652, FDR-corrected *P *= 0.009, median shift = 0.25, 95% CI [0.10, 0.45]), with more templates showing leftward bias than rightward bias. **B** Leftward- and rightward-asymmetric templates in the temporal lobe. Leftward-asymmetric templates exhibited more atypical folding variation than rightward, clustering into smaller and more variable groups that display increased interruptions and branches of the STS; whereas rightward-asymmetric templates displayed a more continuous STS. Specifically, Nos. 2, 3, 4, and 8 showed branches of the middle segments, extending into ITS, while No. 4 also displayed branches of the posterior segments of the STS; **C** Normalised surface area of the temporal lobe for leftward- and rightward-asymmetric clusters. Each point represents one hemisphere. The solid line is the ordinary least-squares regression fit (measure of centre: conditional mean of temporal lobe area given hemisphere area); the shaded band is its 95% confidence interval (1000 bootstrap resamples). The surface area of the temporal lobe increases with the surface area of the entire hemisphere. **D** The normalised temporal lobe surface area was compared, where leftward-asymmetric folding motifs were significantly larger than those of rightward motifs (one-sided permutation test, *n* = 10,000 permutations; *t*(2200) = 13.93; *P* = 1/(10,000 + 1) ≈ 1.0 × 10^−4^; Cohen’s *d* = 0.60; 95% CI [0.51, 0.68]). Source data are provided as a Source Data file.

It is well known that the cortex displays morphological asymmetries, with inter-hemispheric differences in sulcal depth, curvature and thickness that have been linked to functional specialisations such as language, visuospatial processing and handedness^39,40^. Since our templates were generated through joint clustering of left and right hemispheres, this enabled us to posit the question of whether certain cortical patterns were more left- or right-dominated—simply by comparing the frequency of left hemispheres used to build each template. This demonstrated significantly greater asymmetry of the temporal lobe (Fig. 2) relative to the frontal (*U* = 626, *n* = 30 templates per lobe, FDR-corrected *P* = 0.014, median shift = 0.26, 95% CI [0.07, 0.39]) and parieto-occipital regions (*U* = 652, FDR-corrected *P* = 0.009, median shift = 0.25, 95% CI [0.10, 0.45]), with more templates showing leftward bias than rightward bias based on the frequency.

Closer examination showed that leftward-asymmetric templates exhibited more atypical folding variation than rightward, clustering into smaller and more variable groups that display increased interruptions and branches of the STS; whereas rightward-asymmetric templates displayed a more continuous STS. Specifically, No. 2, 3, 4, and 8 in Fig. 2B showed branches of the middle segments, extending into ITS, while No. 4 also displayed branches of the posterior segments of the STS (Fig. 2B); This was confirmed by comparing the normalised surface area of this region, showing that leftward-asymmetric folding motifs were significantly larger than those of rightward motifs (Fig. 2D, *n* = 10,000 permutations; *t*(2200) = 13.93; *P* = 1/(10,000 + 1) ≈ 1.0 × 10^−4^; Cohen’s *d* = 0.60; 95% CI [0.51, 0.68]). Both leftward- and rightward-asymmetric variants showed multiple interruptions of the ITS.

### Pattern and asymmetry replicate in neonates

**Fig. 3:**
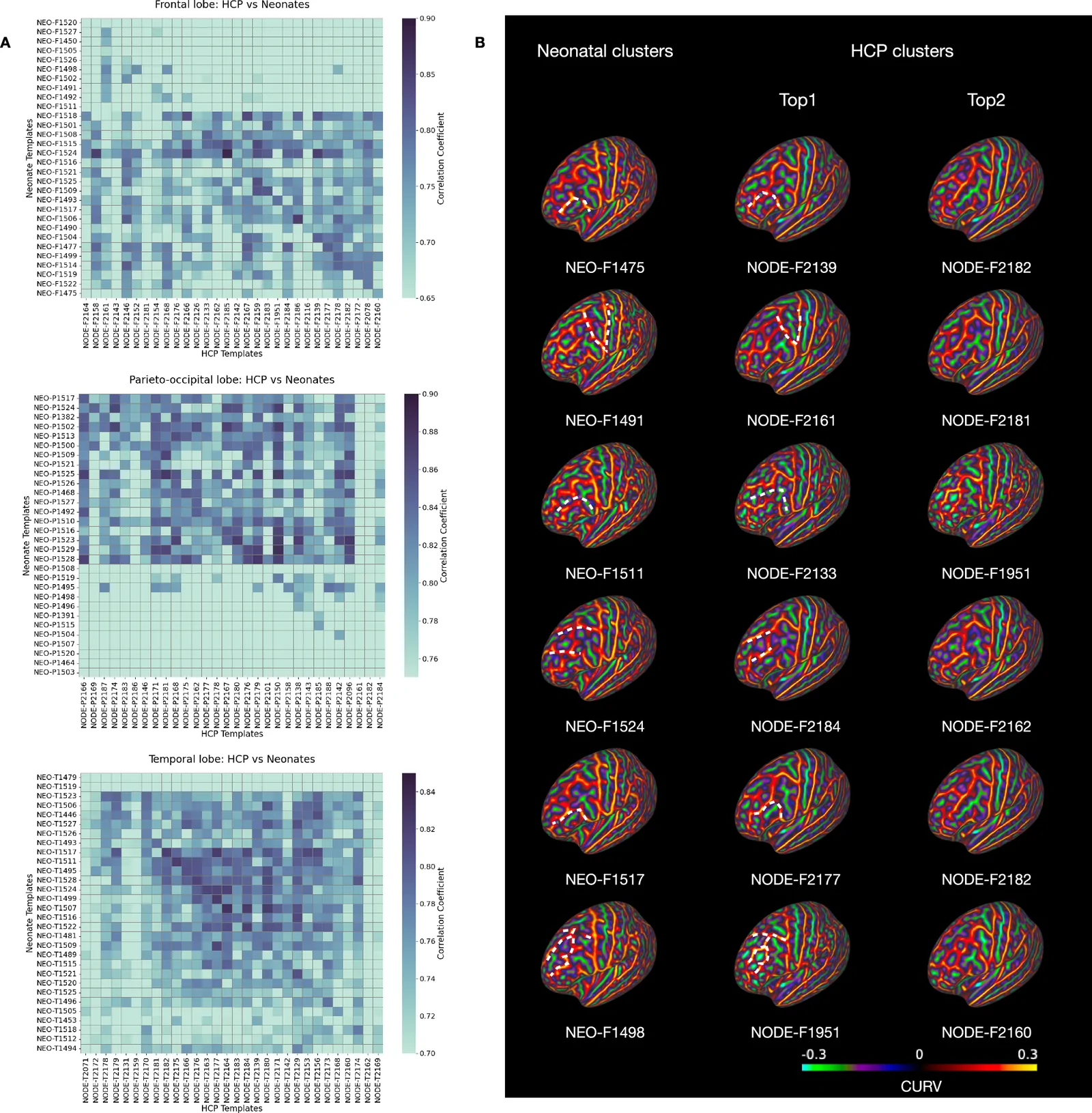
Replication of MSM-HT in the neonatal brain. **A** Correlation similarity between HCP and neonatal folding pattern. The order of the neonates' folding motifs was arranged to show the most similar neonatal motifs close to the diagonal. In almost all pairings, we find a highly correlated match. Source data are provided as a Source Data file. **B** Top matches of neonatal and adult folding motifs. The first column shows the example frontal folding motifs from neonates. The last two columns show the first and second most similar adult folding motifs. The Top 1 column folding motifs show consistent visual resemblance to the neonatal motifs in their characteristic folding patterns, and the Top 2 column motifs show far less pronounced visual similarities. Similar patterns are indicated by the white dashed line. For instance, neonatal cluster NEO-F1475 and HCP cluster F2139 both have small gyri interrupting the IFS and separating the MFS from IFS; neonatal cluster NEO-F1491 and HCP cluster F2161 both have middle frontal gyrus (MFG) detached from the precentral gyrus (PreSG); neonatal cluster NEO-F1511 and HCP cluster F2133 both have PreCS connected to MFS; neonatal cluster NEO-F1524 and HCP cluster F2184 both had parallel branches of MFG.

To evaluate the stability of folding motifs across datasets and development stages, we applied MSM-HT to term-age neonatal scans from the dHCP and BIBS cohorts and compared them to HCP-derived motifs using Pearson correlation of mean curvature maps. In most cases, neonatal motifs showed strong correspondence with specific adult motifs, often with multiple adult templates matching a single neonatal motif (Fig. 3A). Visual inspection confirmed close similarity in key folding features between matched neonatal and adult motifs (Fig. 3B), indicating that the identified patterns are reproduced across development.

**Fig. 4:**
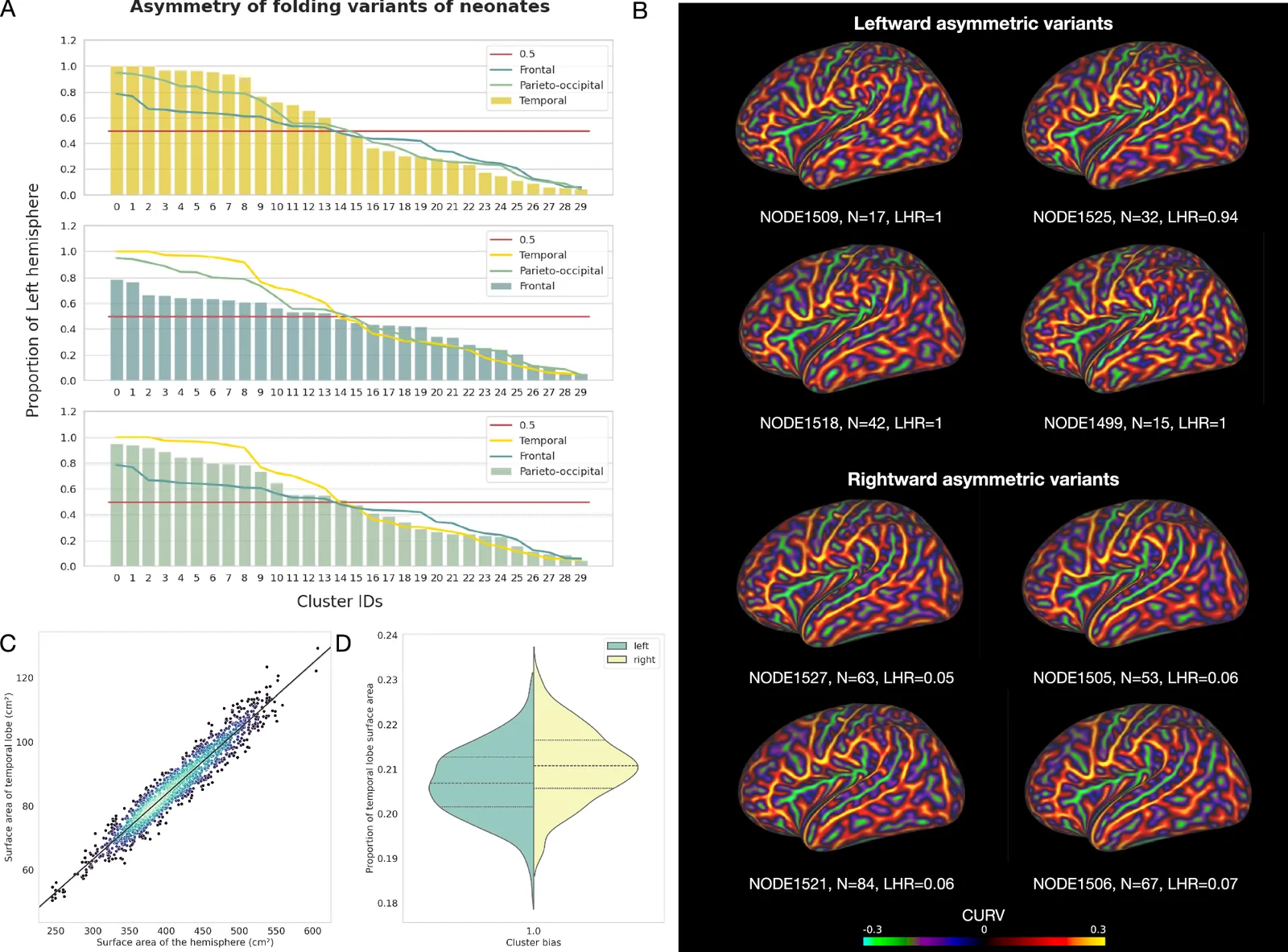
Temporal lobe asymmetry of neonatal datasets. **A** Proportions of the left hemispheres used to construct each template are sorted in descending order. While a trend of greater temporal lobe asymmetry compared to other lobes was observed, it did not reach statistical significance (two-sided Mann–Whitney U test followed by FDR correction, *n* = 30 templates per lobe, temporal vs. frontal *U* = 502, *P* = 0.44, FDR-corrected *P* = 0.62; temporal vs. parieto-occipital *U* = 484, *P* = 0.62, FDR-corrected *P* = 0.62). **B** Leftward- and rightward-asymmetric templates in the temporal lobe. Lobar asymmetry was quantified as the Left Hemisphere Rate (LHR), the proportion of left hemispheres among the examples contributing to each template (range 0–1; 0 = all right hemispheres, 1 = all left hemispheres). The leftward asymmetric folding patterns (high LHR) exhibited more interruptions on the superior temporal sulcus (STS), while the rightward-asymmetric folding patterns (low LHR) tended to have continuous STS. Both leftward- and rightward-asymmetric motifs showed multiple interruptions of the inferior temporal sulcus (ITS). The colour bar is the same as Fig. 3. **C** Normalised surface area of the temporal lobe for leftward- and rightward-asymmetric clusters. Each point represents one hemisphere. The solid line is the ordinary least-squares regression fit (measure of centre: conditional mean of temporal lobe area given hemisphere area); the shaded band is its 95% confidence interval (1000 bootstrap resamples). Similarly to the adults, the surface area of the temporal lobe increased with the surface area of the entire hemisphere. **D** Contrary to the adults, the normalised surface area of the temporal lobe shows that leftward-asymmetric folding motifs were significantly smaller than those of rightward motifs (one-sided permutation test, *n* = 10,000 permutations; *t*(1554) = −9.65; *P* = 1/(10,000 + 1) ≈ 1.0 × 10^−4^; Cohen’s *d* = −0.49; 95% CI [−0.592, −0.390]). Source data are provided as a Source Data file.

We next examined temporal lobe asymmetry in neonates. Although a trend towards greater temporal lobe asymmetry was observed, it did not reach statistical significance (Fig. 4A; two-sided Mann-Whitney U test followed by FDR correction, *n* = 30 templates per lobe, temporal vs. frontal *U* = 502, *P* = 0.44, FDR-corrected *P* = 0.62; temporal vs. parieto-occipital *U* = 484, *P* = 0.62, FDR-corrected *P* = 0.62). As in adults, leftward-asymmetric motifs exhibited more interruptions in the STS, whereas rightward-asymmetric motifs tended to show continuous STS (Fig. 4B). Both leftward- and rightward-asymmetric motifs showed multiple interruptions in the ITS. However, in contrast to adults, leftward-asymmetric motifs showed significantly smaller normalised temporal lobe surface area than rightward motifs (Fig. 4D; *n* = 10,000 permutations; *t*(1554) = −9.65; *P* = 1/(10,000 + 1) ≈ 1.0 × 10^−4^; Cohen’s *d* = −0.49; 95% CI [−0.59, −0.39]). This inverse asymmetry pattern between adults and neonates is consistent with postnatal reshaping of left temporal asymmetry, potentially reflecting environmental influence and language acquisition^41,42^.

### Functional areas lack spatial correspondence with folding patterns

**Fig. 5:**
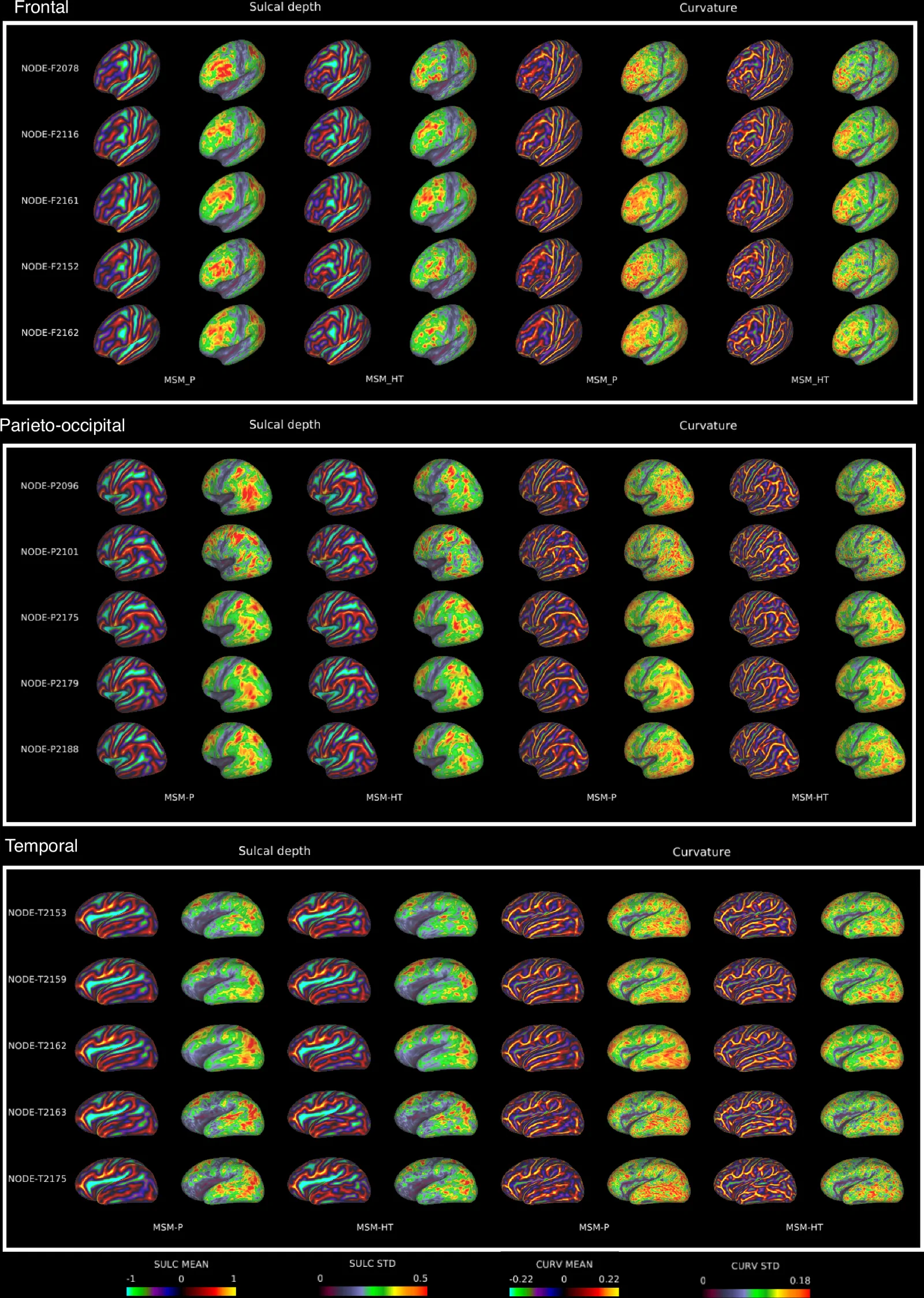
Mean and standard deviation (STD) map of example cortical folding clusters using MSM-P and MSM-HT. Top: example clusters of the frontal lobe. Middle: example clusters of the parieto-occipital lobe. Bottom: example clusters of the temporal lobe. The mean and STD maps of sulcal depth and curvature are compared between MSM-P and MSM-HT: column 1, 2, 3 & 4 are mean and STD maps of sulcal depth, registered with MSM-P and MSM-HT, respectively; column 5, 6, 7 & 8 are mean and STD maps of curvature, registered with MSM-P and MSM-HT, respectively. The colour bars are adjusted to be the same for each of the sulcal depth mean, STD, curvature mean, STD for a fair comparison between MSM-P and MSM-HT.

Cortical folding has long been hypothesised to relate to the organisation of functional areas, with function regionalisation proposed to precede and shape folding patterns^1,43^. As shown in Fig. 5, MSM-HT improves the alignment of structural features, including sulcal depth and curvature, compared to the conventional single-template registration (MSM-P), in which all subjects are aligned to a fixed population-average (MSMSulc) template. The resulting mean maps are sharper and reveal greater detail in the folding patterns. If folding and function are tightly coupled, improved structural alignment would be expected to increase the overlap of functional activation patterns. We therefore tested this by projecting the HCP task and resting-state fMRI into the MSM-HT and MSM-P template spaces.

**Fig. 6:**
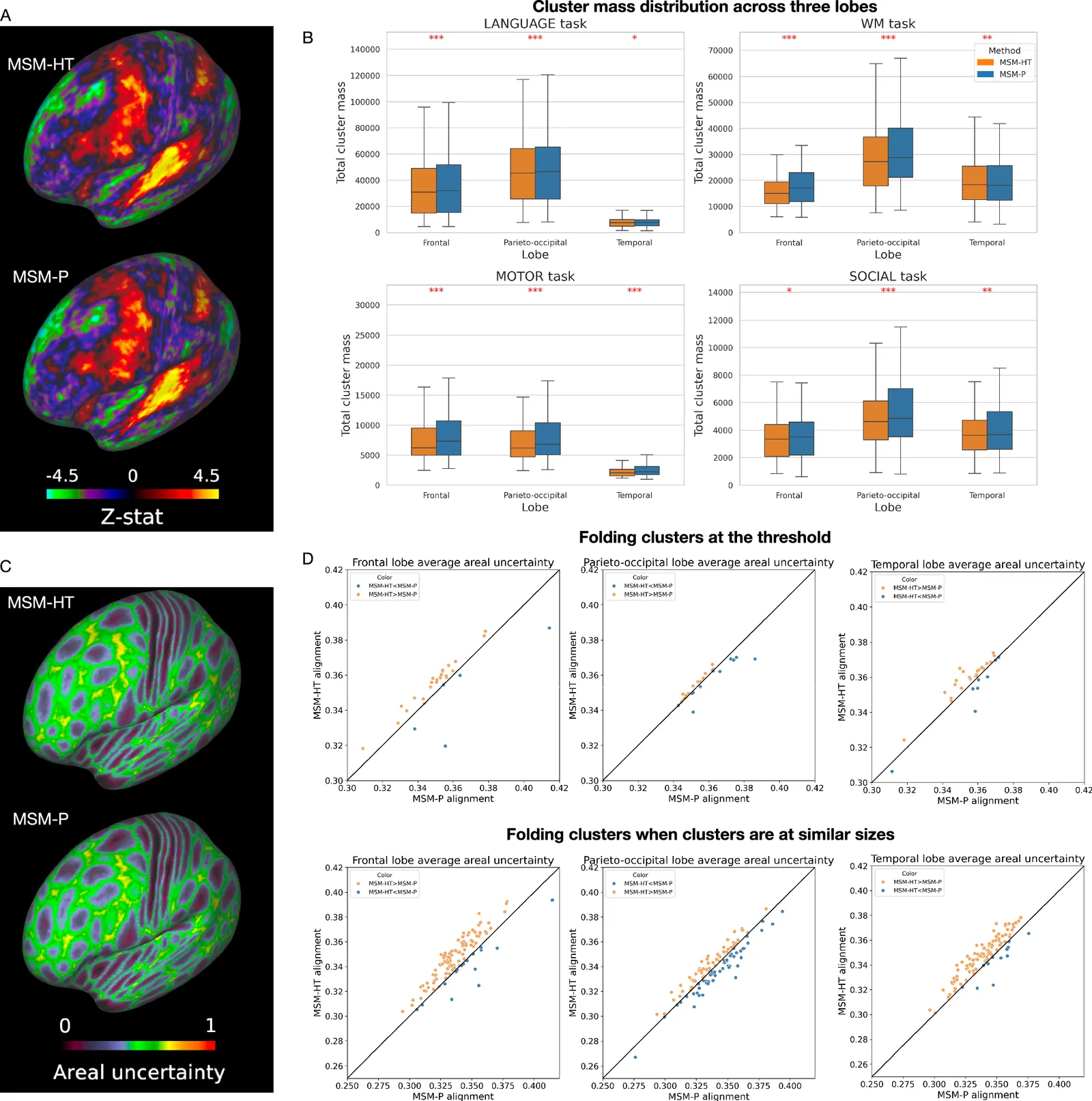
Folding-functional relationship. **A** Example cluster-averaged functional activation for the language-math contrast using MSM-HT and MSM-P (frontal lobe cluster F2161). Functional activations are similarly localised and spatially concentrated under both registration methods. **B** Cluster mass distribution across frontal, parieto-occipital, and temporal lobes for language, working memory, motor, and social tasks. For MSM-HT and MSM-P aligned functional activation maps, box plots of total cluster mass show the median (centre line) and the 25th and 75th percentiles (box bounds); whiskers extend to the most extreme cluster mass within 1.5 × IQR of the box bounds, so the whisker ends mark the minimum and maximum of the displayed data, with more extreme points (outliers) not shown. The unit of analysis is a single folding cluster within a lobar region, treated as a left- or right-hemisphere instance; each cluster is an independent experimental unit (a biological replicate), with no technical replicates pooled or averaged. Within each lobe, MSM-P and MSM-HT cluster mass were compared using a two-sided paired *t*-test; *P*-values were FDR-corrected (Benjamini-Hochberg, *q* < 0.05) across the three lobes within each task. Asterisks denote FDR-corrected significance: ^***^P < 0.001, ^**^P < 0.01, ^*^P < 0.05. *n* = 58 (frontal), 58 (parieto-occipital), 47 (temporal) paired clusters. Exact corrected *p*-values - LANGUAGE: frontal *P* = 4.6 × 10^−13^, parieto-occipital *P* = 1.3 × 10^−9^, temporal *P* = 0.043; WM: frontal *P* = 4.7 × 10^−9^, parieto-occipital *P* = 4.7 × 10^−9^, temporal *P* = 5.0 × 10^−3^; MOTOR: frontal *P *= 2.7 × 10^−8^, parieto-occipital *P* = 5.4 × 10^−9^, temporal *P* = 1.4 × 10^−4^; SOCIAL: frontal *P* = 0.016, parieto-occipital *P* = 3.7 × 10^−8^, temporal *P *= 3.4 × 10^−3^ (full statistical details in Supplementary Table S5). **C** Example multimodal parcellation-based cluster uncertainty using MSM-HT and MSM-P (frontal lobe cluster F2161). **D** Scatter plot comparing within-lobe average areal uncertainty between MSM-HT and MSM-P. The top row shows results for folding motifs defined using a threshold yielding 30 clusters, while the bottom row shows results for motifs matched for comparable cluster sizes. Source data are provided as a Source Data file.

Using the HCP task-based fMRI (tfMRI) data, we aligned language, motor, social, and working memory contrasts with the two different registration methods. The resulting within-cluster group average activation z-stat maps show that functional activations were either similarly localised in both MSM-HT and MSM-P, e.g., for the language-math contrast of the frontal lobe cluster F2161 (Fig. 6A), or in some cases were slightly less well-aligned for MSM-HT. To quantify functional alignment, we used *cluster mass* as a measure of the spatial concentration of task activation across subjects (higher values indicate better overlap). Fig. 6B shows that cluster mass was significantly lower in MSM-HT than MSM-P for frontal and parieto-occipital lobes across all tasks. In the temporal lobe, MSM-HT-aligned cluster mass was significantly lower for the language, motor, and social tasks, but significantly higher for working memory (paired two-tailed *t*-tests, FDR-corrected across the three lobes within each task, all corrected *P* < 0.05; full statistical details in Supplementary Table S5). These results indicate that improved structural alignment does not translate into improved functional correspondence, particularly in association cortices. This could be explained by the fact that the placement of architectonic areas is not constrained by morphological landmarks (sulci and gyri)^21^. Quantitative analyses confirmed that folding motifs show stronger structural coherence within clusters (Cohen’s *d* = 1.41–1.67 across lobes), whereas functional similarity shows minimal differentiation between clusters across lobes (Cohen’s *d* = 0.05−0.09; Supplementary Table S3). The coupling coefficient between structural and functional similarities ranged from *ρ* = 0.068–0.104 (all permutations *P* = 2 × 10^−4^, the floor for 5000 permutations; full statistical details in Supplementary Table S2).

To assess functional alignment beyond the four tasks, we used the spatial mappings from MSM-HT and MSM-P to align individual HCP multimodal cortical parcellations^44^. These parcellations integrate a broader set of functional features, including resting-state networks, T1w/T2w myelin maps, and cortical thickness, providing a more comprehensive view of functional organisation. Fig. 6C shows the areal uncertainty for an example cluster (F2161) under both registration methods. Average areal uncertainty is similar between MSM-HT and MSM-P (Fig. 6D), consistent with the tfMRI results. This further indicates that improved alignment of cortical folding does not mean improved functional overlap. Instead, additional factors may drive functional alignment, such as connectivity and plasticity.

## Discussion

Patterns of cortical folding result from the long process of structural and functional cortical development and offer a window into misdevelopment. Our study was motivated by a need to uncover a comprehensive baseline of “typical" cortical folding, to increase our sensitivity to detect morphological motifs of behaviour and disease. To achieve this, we parsed heterogeneous patterns of cortical folding into a discrete set of typical templates for each anatomical lobe. Previous study proposed that cortical folding patterns are organised around reproducible elementary units, including sulcal roots^43^ and deep sulcal landmarks^45,46^, which remain relatively stable across individuals despite substantial variability in the overall folding. In this context, our motifs can be interpreted as the higher-order configuration of these stable landmarks, capturing how these elements are arranged and combined into complex patterns across individuals. Our motifs aligned with variants manually defined by^6^, and our results uncovered both common cortical variants (i.e. seen in at least 5% of the sample) and known, but rarer, variants (such as the single-segment PreCS in cluster F2126). This anatomical foundation provides a framework for exploring structural brain organisation at a larger scale.

Our motifs, identified in adults, are representative of the earliest stages of post-natal life. The close resemblance between the identified neonatal and adult folding motifs suggests the developmental consistency of major cortical folding patterns established prenatally or shortly after birth across different life stages. However, there is evidence that temporal lobe folds are still under fast development at this stage^47,48^. For instance, comparing term-age neonates and adults, Hill et al.^41^ reported that the lateral temporal and anterior cingulate folding can still be curving and branching after birth. In our results, while some folding patterns in neonates mirrored those found in adults, the diversity of these patterns was notably less in the neonatal brain, especially for the frontal and temporal lobes. Importantly, the present study is cross-sectional rather than longitudinal; therefore, the comparison between neonates and adults reflects differences between cohorts rather than direct developmental trajectories within individuals.

Cortical asymmetry has long been recognised, particularly in the planum temporale, which is typically larger in the left hemisphere and linked to language processing^49,50^. More recent work has identified asymmetries in the STS, including a deeper right STS and more frequent interruptions in the left, suggesting genetically constrained features related to language networks^51–53^. Beyond previous studies that characterised asymmetry using scalar measures (e.g., sulcal depth, regional size) following alignment to a single population-average template^50,51^, or reported greater interruptions and sulcal pits in the left STS^45,52^, our approach captures asymmetry in the spatial organisation of cortical folding patterns. Such scalar summaries describe local geometric properties but do not capture how folds are arranged across the cortical surface, particularly in regions with inconsistent sulcal alignment. In contrast, our clustering-based framework characterises asymmetry at the level of folding motif organisation. Using this approach, we observed greater folding variability in the left temporal lobe, particularly in the STS, where leftward-asymmetric motifs exhibit more branching and interruptions by plis de passage. This asymmetry is evident in both neonates and adults and extends previous findings by showing how such differences manifest in the organisation and prevalence of distinct folding motifs. The increased structural complexity on the left may provide a substrate for the lateralisation of language processing, supported by evidence linking plis de passage to local U-shaped fibre connectivity^54^. In line with this, we discovered significantly higher normalised temporal lobe surface area in the left hemisphere than the right in adults, indicating more computation related to language processing.

Notably, temporal lobe asymmetry differs at the two age points. In early development, the right temporal lobe has been shown to mature earlier than the left in terms of cortical folding complexity^42^. Our neonatal results are consistent with this pattern, showing less pronounced temporal lobe asymmetry relative to other lobes and fewer leftward-asymmetric templates relative to adults. In addition, rightward-asymmetric folding clusters exhibited higher temporal lobe surface area than leftward-asymmetric clusters in neonates, whereas this pattern was reversed in adults. This aligns with^55^, who reported stronger leftward surface area asymmetry in adults compared to neonates along the STS and STG. Functionally, the right temporal lobe is associated with facial recognition and social cognition^51^ and appears to be more genetically constrained and established at birth^56^. In contrast, the left temporal lobe supports language processing^51^ and is more strongly shaped by postnatal experience, such as language exposure^42^. As development progresses, experience-dependent plasticity and synaptic pruning may shift the relative importance of these functions, with later stages placing greater demands on language-related processing. We therefore hypothesise that these processes contribute to a gradual shift in hemispheric temporal lobe surface areas, leading to more pronounced leftward asymmetry observed in adulthood.

It therefore seems that, from the link between structural asymmetry and functional lateralisation, the brain cortical folding pattern is associated with functional organisation. Indeed, theories have been proposed that suggest that early placement of cortical folds might relate to the formation of functional regions^1,43^. However, this folding-functional link hypothesis has been validated mostly in primary functional areas, including the primary motor and visual cortex^19^; whereas it has become clear from our study and others^20,41^ that the folding-function relationship in higher-order areas is more complicated. In these areas, folding is much more irregular, and their correspondence with functional organisation is much weaker. Supplementary analyses further quantified this relationship, showing that the correlation between folding similarity and functional similarity across individuals is small (*ρ* = 0.068−0.104 across lobes), indicating that folding patterns explain only a limited proportion of inter-individual variability in functional organisation. One possible interpretation for these results is that cortical folding aligns more with primary functional regions because early-forming folds are under stronger genetic control, whereas later-forming folds in association cortex are influenced by more variable biomechanical and environmental factors. In line with previous heritability research in HCP^4^, our study found a stable genetic influence within lobes in the adult brain, indicated by MZ twins being significantly more likely to fall into the same folding clusters than DZ twins. The ACE/AE models also exhibit moderate heritability across lobes. Indeed, cortical areas are influenced by genetic patterning that contributes to the early organisation of the functional regions, where early cortical folds are formed related to them^1,43,57^. Furthermore, recent studies show that gene expression is highly localised prenatally, and this persists towards adulthood^58^. The genetic gradient also sharply changes across the first-forming sulci^59^. Altogether, these studies suggest that genetic control might explain why the first-forming sulci along the time course exhibit less between-individual variability than later-forming sulci. However, later in the folding process, the competition between genetic control and biomechanical pressures might start to bias towards the latter since gene transcription becomes more synchronised across regions in the late prenatal stage^60^. The balance between different biomechanical forces, such as local cell proliferation and white matter tract tethering, can be highly sensitive^3^; small differences in the earlier developmental stage (e.g., variations in proliferation) and later neuron positioning through migration can result in significant changes in later-forming folds, leading to highly irregular sulci^59,61^. On the other hand, while overall brain shape (primary and secondary folds) is mostly fixed at birth, functional development continues into adulthood; therefore, functional plasticity may be another factor in the final placement of functional areas. The early positioning can be regulated by signalling molecules discharged from multiple patterning centres^62^. Moreover, multiple environmental factors such as social status, language exposure, and pathological circumstances can influence the development of association networks and higher-order functions throughout childhood and adulthood^63–65^. Together, these findings suggest that cortical folding motifs reflect developmental constraints that interact with genetic, biomechanical, and environmental factors, rather than deterministically defining functional organisation.

MSM-HT represents a powerful framework for uncovering and characterising cortical folding patterns, providing insights into the complex relationships between brain structure, function, and development. However, several limitations should be considered. Comparison with Ono’s atlas should be interpreted with caution. The lobe-wise clustering approach provides a holistic characterisation of folding variability but lacks the spatial resolution to capture fine-scale variations within individual sulci described in postmortem studies, limiting direct comparability. Developmental comparisons between neonates and adults also require careful interpretation, as these analyses are cross-sectional. Observed differences in motif incidence may reflect both biological maturation and methodological factors, including the lower spatial resolution relative to the brain scale, which reduces the detectability of finer sulcal branches and interruptions. In addition, the absence of tfMRI in neonates, together with inflated partial-volume effects in neonatal fMRI^66^, limits direct structure-function comparisons across developmental stages. The functional measurements used in this study, including tfMRI contrasts and HCP_MMP1.0 parcellations, may not fully capture the complexity of functional organisation. Future work incorporating complementary representations, such as continuous functional gradients, can avoid using discrete network boundaries. Finally, both cohort characteristics and analytical choices may influence the generalisability of these findings. The HCP-YA is relatively homogeneous in ethnicity and age, while neonatal cohorts (dHCP, BIBS) have narrow age ranges. In addition, the hierarchical registration and clustering pipeline involves design choices, such as the order of lobe alignment, that may affect the resulting motifs. In this regard, dHCP is now being followed longitudinally into childhood, providing an important opportunity to directly examine the developmental evolution of cortical folding motifs. Future work should expand MSM-HT to more diverse and longitudinal datasets, and integrate multimodal data, including functional connectivity and genetic information, to better characterise developmental trajectories and improve the identification of robust biomarkers.

## Methods

This research complies with all relevant ethical regulations. The study used previously collected, de-identified data from three cohorts and did not involve new data collection. The HCP-YA study was approved by the Institutional Review Board of Washington University in St. Louis (IRB #201204036). The dHCP was approved by the UK Health Research Authority (Research Ethics Committee reference 14/LO/1169). The BIBS was approved by the London - Dulwich Research Ethics Committee (reference 12/LO/2017).

### Datasets

The data used for the development and validation of the proposed method correspond to structural and functional imaging data from 2220 cortical hemispheres derived from the young adult HCP. Cortical folding templates were subsequently replicated in a neonatal cohort (1562 hemispheres) derived from the dHCP and BIBS.

#### HCP Young Adult

Data were collected from 1110 young adults aged 28.8 ±3.7 years (604 biological females) on a Siemens Skyra “Connectom” scanner, with a standard Siemens 32-channel head coil^67^. Written informed consent was obtained from all participants. T1w scans were acquired with a 3D MPRAGE sequence (TR = 2400 ms, TE = 2.14 ms, TI = 1000 ms, voxel size = 0.7 mm isotropic); whereas, T2w scans were acquired with a 3D T2-SPACE sequence (TR = 3200 ms, TE = 565 ms, voxel size = 0.7 mm isotropic). For functional acquisition, resting-state fMRI was acquired with a Gradient-echo EPI sequence (TR = 720 ms, TE = 33.1 ms, flip angle = 52 degrees, multiband factor 8, 60 min for 4 runs, voxel size = 2 mm isotropic); tfMRI was acquired with the same sequence parameters as resting-state fMRI (7 tasks including Language, Working Memory, Motor, Social Cognition, Gambling, Emotion Processing, Relational Processing, 48 min total). Full details regarding acquisition and participant inclusion criteria are described in^68^. All data is open and accessed through ConnectomeDB.

#### dHCP and BIBS

dHCP and BIBS are parallel studies run at the Evelina London Children’s Hospital, St Thomas, London, that dual-consented participants; dHCP was a community cohort which included preterm-born children, and BIBS recruited neonates with and without familial history of neurodevelopmental and/or psychiatric conditions such as autism, attention deficit hyperactivity disorder (ADHD), or mood disorder. Full inclusion criteria for dHCP and BIBS control subjects are described in^69^. All datasets were acquired on a Philips Achieva 3T scanner at the Evelina Newborn Imaging Centre with a dedicated 32-channel neonatal head coil^69,70^. A total of 817 neonatal scans were available from the combined dHCP and BIBS cohorts. For the present study, we restricted the analysis to scans acquired at term-equivalent age (postmenstrual age ≥38 weeks), resulting in 781 scans. Of these, 648 were term-born (scan age 41.5 ± 1.7 weeks, 297 female) and 133 were preterm-born (scan age: 41.3 ± 1.7 weeks, 59 female). Among the BIBS cohort, 44 had a known family history of autism, 22 had a known family history of ADHD, and 10 had a mother with a mood disorder. Structural T2w MRI was acquired with a TSE sequence: TR = 12000 ms; TE = 156 ms; SENSE factors: 2.27 (axial) and 2.66 (sagittal); in-plane resolution = 0.8 × 0.8 mm, slice thickness: 1.6 mm; slice overlap = 0.8 mm.

### Image pre-processing

#### HCP

Adult data were obtained from the HCP and were processed using the HCP minimal preprocessing pipeline^68^, which includes Freesurfer-based cortical surface reconstruction^71^. This pipeline performs tissue segmentation, extraction of white and pial surfaces, inflation, and spherical projection. Sulcal depth maps and mean curvature maps were derived from the reconstructed white surfaces, which summarise coarse-scale and fine-scale cortical folding correspondingly, and cortical labels were obtained from the Desikan-Killiany atlas^72^ with 34 gyri-based regions. All native surfaces were resampled to an ico-6 mesh (with a resolution of 40,962 vertices) for further analysis. Functional data were obtained from the HCP task-fMRI dataset, which is provided in the HCP grayordinate space aligned using MSMAll registration. In the HCP pipeline, the timeseries first underwent motion correction and distortion correction; then the voxels within the cortical ribbon were mapped to the cortical surface, transformed to the 32k_FS_LR mesh with surface registration, and smoothed within the surface with a 2mm FWHM Gaussian kernel. The task contrasts were estimated within each run, then each subject, using general linear model (GLM) analyses implemented in FEAT^73,74^. For the purpose of this study, the MSMAll alignment was inverted to recover subject-specific functional maps in native spherical space.

#### dHCP and BIBS

Neonatal surfaces were extracted using a recently published bespoke deep learning-based pipeline^75^, trained on the outputs from the original dHCP pipeline^76^. This pipeline adapts the FreeSurfer pipeline to the very different MRI properties of neonatal scans, which have inverted tissue contrast and relatively low resolution. The surface inflation and feature extraction in dHCP deep learning is a direct reimplementation of Freesurfer^71^. Visual quality control (QC) of the surface output from the learning-based pipeline was improved in terms of anatomical and topological correctness^75^. We use neonatal spheres, sulcal depth, mean curvature, and cortical labels from the DRAW-EM regional atlas^77–79^, which differs from the Desikan-Killiany atlas, but ensures accurate segmentation of the neonatal brain structures that is appropriate to the different contrast of neonatal MRI and shape of neonatal cortex. For more details about the deep learning-based neonatal pipeline, see Supplementary Methods S1.1.

### Hierarchical MSM registration (MSM-HT)

#### Overview

MSM-HT leverages the complementary benefits of two separate tools for cortical surface registration: MSM^36–38^ and its learning-based implementation (Deep-Discrete spherical Registration, DDR)^35,80^. Both methods solve the same objective: deforming an input (or moving) mesh until its features (e.g., sulci and gyri) better overlap with those of a reference surface whilst penalising extreme (biologically implausible) deformations; however, while MSM offers theoretical guarantees over the smoothness and invertibility of its deformation fields, DDR offers fast approximate solutions that are better suited to performing pairwise alignment between all cortical hemispheres, in large cohorts. For this reason, we used DDR to coarsely align large numbers of pairs of hemispheres and MSM to generate the final templates. For more details on each of these methods, see^35–37,80–82^.

**Fig. 7:**
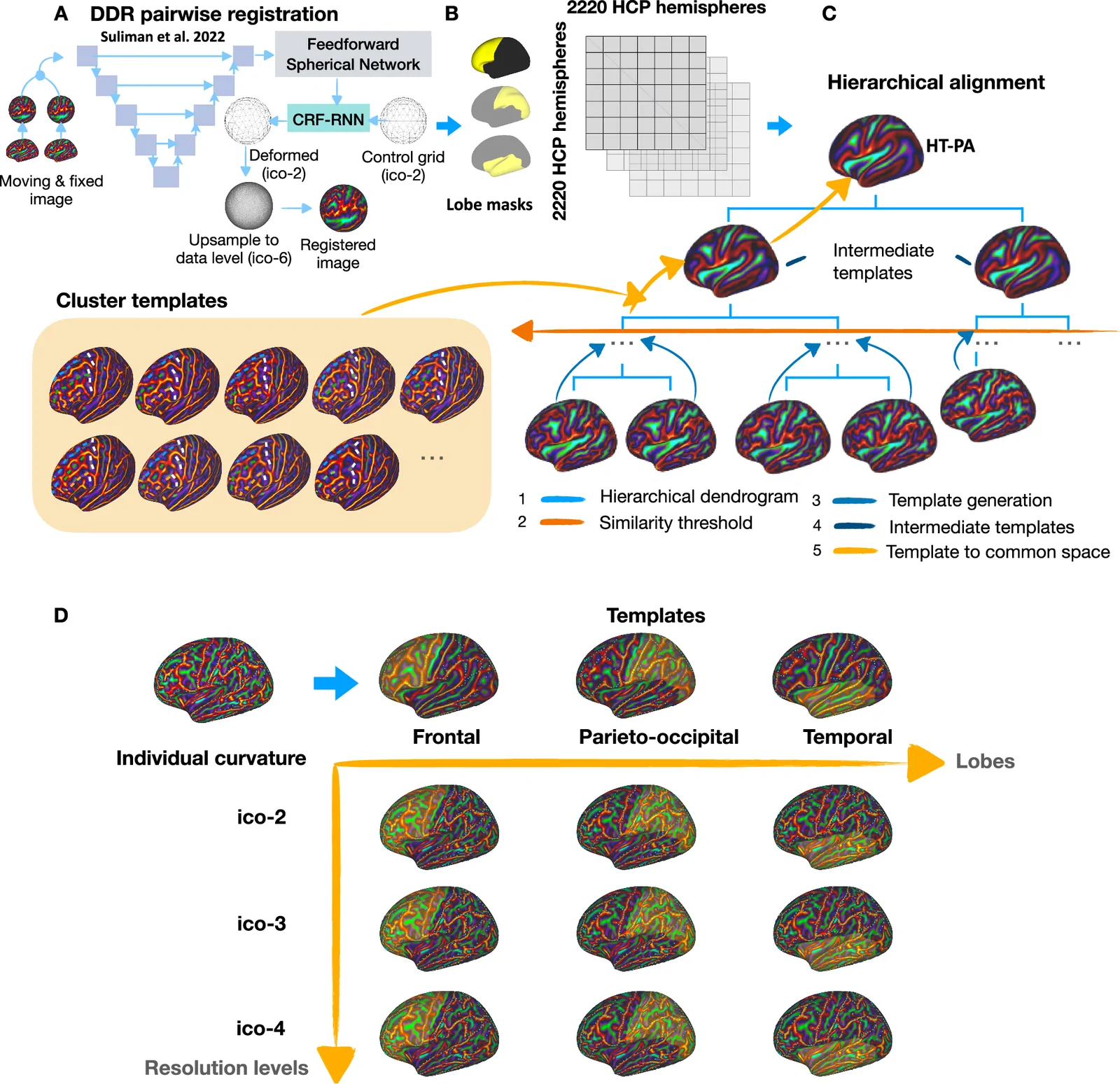
Overview of multimodal surface matching with hierarchical templates (MSM-HT). **A** After mirror-flipping all right hemispheres (into rigid alignment with left hemispheres), all pairs of sulcal depth maps were co-registered using DDR. **B** Once sulcal depth maps are coarsely aligned, the overlap of cortical folding patterns, for each pair of hemispheres, is separately assessed for each of the frontal, parieto-occipital, and temporal lobes—using a combination of Dice overlap and cross-correlation. **C** Hierarchical alignment in steps. (1) Agglomerative hierarchical clustering is applied to each of the resulting similarity matrices, (2) The hierarchy is thresholded at the similarity score that returns thirty clusters for each lobe, (3) MSM is then used to co-register curvature maps of individuals within each cluster to generate a template for each cluster—that summarises the shape of folds within that cluster, (4) Intermediate templates were generated at each node of the dendrogram, by aligning pairs of templates until all images were merged into Hierarchical Templates Population Average (HT-PA) at the top. (5) Finally, all templates are further registered through the hierarchical path to a common space defined by the HT-PA. **D** Combining the registration across three lobes. Mappings are combined across lobes by iterating alignment: first to frontal lobe templates, then parieto-occipital, and finally temporal. This process is repeated through 3 resolution levels of the MSM registration from coarse to fine.

The resulting framework of MSM-HT is shown in Fig. 7. Starting with the null hypothesis that there is no asymmetry of the cerebral cortex, we first mirror-flip all right hemispheres (into rigid alignment with left hemispheres) using Workbench Command; then co-register all pairs of sulcal depth maps using DDR. Once sulcal depth maps are coarsely aligned, the overlap of cortical folding patterns for each pair of hemispheres may be separately assessed for each of the frontal, parieto-occipital, and temporal lobes - using a combination of Dice overlap and cross-correlation (CC). The definition of these lobar regions is shown in Supplementary Fig. S13. Agglomerative hierarchical clustering is then applied to each of the resulting similarity matrices, and the hierarchy is thresholded at the similarity score that returns thirty clusters for each lobe. MSM is then used to co-register curvature maps of individuals within each cluster to generate a template for each cluster that summarises the shape of folds within that cluster. Finally, all templates are further registered through the hierarchical path to a common space.

#### Optimising pairwise alignment with DDR

DDR is a deep learning-based cortical surface registration algorithm that consists of a rigid-body rotation network and a non-rigid deep-discrete deformation network. Both networks take the form of a U-Net surface convolutional neural network (CNN) operating on the spherical mesh domain. The rotation network learns to rigidly align input surfaces to a single population-average template, while the non-linear deformation network learns a spherical displacement field that aligns an input (moving) sulcal depth map with a target (reference) sulcal depth map. The displacement field is parameterised on a control grid defined on a lower-resolution icosphere than the input feature maps. In this work, the control grid is set to ico-2 (162 vertices), while input and reference feature maps are represented on the ico-6 mesh (40,962 vertices). In the original DDR framework^35^, the reference map corresponds to the HCP population-average MSMsulc template; in the present study, however, the deformation network was retrained to perform pairwise alignment between individual subjects. The network was trained using a combination of mean squared error (MSE) loss to optimise the rotation network and a combination of MSE and CC loss functions. Once trained, the DDR model was applied to compute pairwise alignments between all hemispheres in the dataset. The resulting deformations were used to evaluate the similarity of cortical folding patterns across subjects. Further details on the training procedure and implementation of DDR are provided in the Supplementary Methods S1.2 and in ref. ^35^.

#### Clustering of cortical folding patterns

Following DDR alignment between all pairs of hemispheres, the similarity of cortical folds was assessed from a combination of CC of the entire sulcal depth map and Dice overlap of the gyral crown. Since Dice is typically used as a measure of segmentation overlap, we first thresholded the sulcal depth at the 30th percentile (to retain only gyri) and then binarised the result to form a segmentation mask. These similarity metrics were chosen because CC can be seen as being sensitive to the similarity of the general pattern of features. In contrast, Dice is more sensitive to the location of the sulci and gyri. The final similarity score was computed as the average of the CC and the Dice. This resulted in a similarity matrix *S* for each lobe. Individual hemispheres could then be grouped using agglomerative hierarchical clustering. Agglomerative hierarchical clustering was implemented using the Python library scikit-learn (version 1.0.2), with complete linkage and distance defined as 1 − *S*. This establishes a dendrogram which encodes the distance between different surfaces based on the similarity matrix (see Supplementary Fig. S10 and S11). Initially, each surface stands as its own cluster. Clusters are iteratively merged in ascending order of inter-cluster distance until a hierarchical path interconnects all data points. We then threshold each dendrogram at the level of 30 clusters (clusters containing fewer than five hemispheres were excluded for the downstream analyses, leaving *n *= 2190 of 2220 hemispheres), where 30 was chosen as it was felt that this represented a manageable number of templates—with sufficient detail to compare against^62^—without underpowering individual cluster analyses. The resulting cluster assignments define groups of hemispheres with similar folding patterns, which are subsequently used to generate folding templates using the MSM-based hierarchical registration framework described below. Please see Supplementary Methods S1.3 for a detailed explanation of the agglomerative hierarchical clustering, the choices of clustering parameters, and the evaluations of alternative clustering methods.

#### Template generation

Once cluster membership was defined, templates were generated using a coarse-to-fine alignment procedure implemented with MSM. This optimises warps over a series of control-point grids of increasing resolution, from ico-2 to ico-4 (2562 vertices), first performing highly regularised alignment of major folds before refining the alignment of finer-scale details. Warps are optimised through discrete optimisation, which balances a data similarity (feature overlap) measure with a regularisation term that penalises non-biologically-plausible deformations^37^. Data similarity is implemented using CC, while regularisation is through the hyperelastic strain of the deformation field. The balance between these terms is controlled by a tunable hyper-parameter *λ*. In this work, we use a recent implementation of MSM^38^, which provides improved computational efficiency while preserving the optimisation framework of the classic MSM^36,37^. We used version v1.0 of the newMSM, with the regularisation parameter *λ* set to 0.1. Full parameter configuration files for all MSM runs are available in the public repository (https://github.com/Yrong-Guo/Hierarchical-surface-registration/tree/main/msm_config). To optimise the templates’ sharpness, newMSM was iterated over multiple rounds, with the first round driven by sulcal depth maps and subsequent rounds driven by curvature. This follows the standard protocol for template generation used by^83,84^.

#### Hierarchical alignment to population-average space

While the previous stage is sufficient to co-align hemispheres within each cluster, it presents no guarantees that cortical maps align between clusters. Thus, to enable comparison of features across all clusters, we first generate intermediate templates: following the MSM-HT process up to the top of the dendrogram, by aligning pairs of templates until all images are merged into a full population-average (HT-PA) at the top.

Next, individual hemispheres are brought into HT-PA space, by incrementally aligning each example through the template hierarchy. Since templates are defined separately for each lobe, this requires masking of the cortical feature maps to ensure that only folds from the lobe in question are used to drive alignment to each template. Then, mappings are combined across lobes by iterating alignment: first to frontal lobe templates, then parieto-occipital and finally temporal. Since regularisation can cause deformation at the margins of the lobar masks, potentially affecting neighbouring regions, its impact is mitigated through iterating template alignments for each of the 3 resolution levels of the MSM registration (Fig. 7D). While this process may seem elaborate, it is necessarily complicated due to the vast heterogeneity of folding patterns and the limited co-occurrence of patterns across lobes. This iterative approach prevents the registration of one lobe from adversely influencing other brain regions, maintaining the integrity of the lobar-specific alignments.

### Lobar folding asymmetry

Lobar asymmetry was assessed by calculating the proportion of left hemisphere examples used to generate each template. Referred to as the Left Hemisphere Rate (LHR), this returned a value between 0 and 1, where 1 indicates that all the hemispheres in a cluster are left hemispheres. Clusters were categorised as leftward- or rightward-asymmetric based on whether their LHR was greater than 0.5. We used the two-sided Mann-Whitney U Test to determine the significance of the difference between each pair of lobes. P-values were FDR-corrected for multiple comparisons (Benjamini-Hochberg), with significance assessed at *q* < 0.05. On observation of significantly greater asymmetry of the temporal lobe, we characterised the differences through manual labelling and quantification of the rate of branches and interruptions in each template. We also estimated the total surface area of this region (defined by Desikan-Killiany atlas), normalised by the total ipsilateral surface area. The significance of this difference was evaluated using a two-sample t-test.

### Generalisation to developmental cortical folding

For investigating cortical folding variants in neonates, we replicated the MSM-HT framework (described in Hierarchical MSM registration (MSM-HT)) in the dHCP and BIBS cohorts. Since neonatal cortical feature maps display very different imaging properties, the DDR deformation network was retrained on the dHCP dataset, utilising the same architecture and optimisation procedures with the same parameters as the HCP. Lobar masks were generated from the DRAW-EM neonatal atlas^77–79^. MSM-HT was then run and evaluated in exactly the same way as for the HCP-YA data. Finally, neonatal and adult templates were paired to assess the similarity of each pair in the HT-PA space (assessed from cross-correlation).

### Heritability of the cortical folding motifs

To assess the genetic contribution to cortical folding motifs, we performed twin modelling using the HCP twin cohort. For each motif, similarity between an individual hemisphere and the motif template was quantified as the CC between the subject’s sulcal depth map and the motif template map. Motifs represented by fewer than 50 hemispheres were excluded to ensure stable estimation. Twin analyses were conducted using structural equation modelling implemented in the OpenMx package (version 2.22.9) in R 4.5.1. We fitted bivariate ACE models to jointly model left and right hemispheres, partitioning phenotypic variance into additive genetic (A), shared environmental (C), and unique environmental (E) components and estimating the genetic correlation between hemispheres. Nested AE models were compared with the full ACE model using likelihood-ratio tests and Akaike information criterion (AIC); when shared environmental effects did not improve model fit, the AE model was retained. From the final model, we report heritability estimates (*h*^2^) for left and right hemispheres and the genetic correlation (*r*_*g*_).

### Folding-function relationship

We hypothesised that improved alignment of cortical folds would lead to improved alignment of functional activations, given the partial correspondence between functional regions and cortical folding. Under this assumption, individuals within the same folding cluster would be expected to exhibit more similar cortical areal topographies, as estimated from the HCP multimodal cortical areal parcellations (HCP_MMP1.0). To test this, the deformation fields derived from MSM-HT and conventional single-template registration (MSM-P) were used to project tfMRI data and individual HCP_MMP1.0 parcellations into a common template space. MSM-P corresponds to alignment to the standard MSMSulc template used in the HCP pipelines (available at: https://github.com/Washington-University/HCPpipelines/tree/master/global/templates/standard_mesh_atlases). For tfMRI, we used specific contrasts from the Language (Story, Math), Working Memory (N-back tasks), Social Cognition (Theory of Mind), and Motor (movement of left/right hand, left/right foot, tongue) tasks. These contrasts were chosen for their well-documented spatial activations. The group-average tfMRI activation was calculated using a GLM-based fMRI analysis with mixed effects FLAMEO^74^. Cluster mass was used to measure the strength and spatial concentration of significantly activated vertices (as defined in ref. ^37^), where higher values indicate better overlap of tfMRI activations across the population under consideration. The area distortion of both MSM-HT and MSM-P was controlled to be equivalent (Supplementary Methods S1.3), in order to exclude the possibility that the strength of the shape-driven surface registration might influence functional overlap. The significance of the difference between MSM-HT and MSM-P was evaluated using two-sample t-tests, followed by an FDR of 0.05 for multiple comparisons. Additional analyses quantifying folding-function coupling, including within/between-cluster similarity comparisons and Mantel correlation tests between folding and functional similarity matrices, are reported in Supplementary Results S2.3.

Individual subject HCP_MMP1.0 parcellations were downloaded from the BALSA database. These define cortical areas from a combination of multi-modal areal features, including myelin maps, cortical thickness, tfMRI contrast maps, resting-state fMRI functional connectivity (FC) maps, and continuous, topographically organised gradients of resting-state fMRI functional connectivity^44^. Thus, these represent a more comprehensive measure of cortical organisation than tfMRI alone. Overlap of areal features across individuals after alignment was assessed by the areal uncertainty of each vertex: calculated as one minus the highest proportion of individuals sharing the same label at that location. To ensure that the areal uncertainty was not inflated by large cluster sizes, we calculated it both on the original clusters and after breaking the clusters into comparable sizes based on the dendrogram.

### Reporting summary

Further information on research design is available in the Nature Portfolio Reporting Summary linked to this article.

## Supplementary information

Supplementary Information

Description of Additional Supplementary Files

Supplementary Data 1

Supplementary Data 2

Supplementary Data 3

Supplementary Data 4

Supplementary Data 5

Supplementary Data 6

Supplementary Data 7

Reporting Summary

Transparent Peer Review file

## Source data

Source Data

## Data Availability

The adult data used in this study are obtained from the Human Connectome Project Young Adult release (S1200). Preprocessed surfaces were obtained from the HCP ConnectomeDB repository (https://db.humanconnectome.org) under the HCP Open Access Data Use Terms, which require registration and acceptance of a data use agreement prior to download. The neonatal data used in this study (T2w images and DRAW-EM labels) are available from the Developing Human Connectome Project (dHCP) fourth release available through https://biomedia.github.io/dHCP-release-notes/, which requires registration and acceptance of the dHCP open-access data use agreement. The BIBS data used in this study are distinct from the dHCP release and are not deposited in the NDA. These data cannot be shared openly, as participating families did not consent to open data sharing. Access is restricted to the BIBS study team and approved collaborators, governed by the study’s ethical approval (London - Dulwich Research Ethics Committee, reference 12/LO/2017); inquiries should be directed to the BIBS study team (bibs@kcl.ac.uk and grainne.mcalonan@kcl.ac.uk). After quality control and preprocessing, the final datasets included 1110 adult subjects from HCP-YA, 693 neonatal subjects from dHCP, and 88 subjects from BIBS. MSM-HT motifs can be freely downloaded from https://balsa.wustl.edu/study/ljVNl. The MSM-HT deformation spheres are publicly available at Zenodo (DOI: 10.5281/zenodo.20507642). We have satisfied the Nature Machine learning reporting checklist, which is provided with the submission. Source data are provided with this paper.

## Code availability

Code for MSM-HT, trained DDR pairwise registration models, and the analysis scripts used in this study are publicly available at https://github.com/Yrong-Guo/Hierarchical-surface-registration. The DDR registration repository is available at https://github.com/mohamedasuliman/DDR. Surface registration was performed using the newMSM implementation (v1.0), available from the FSL repository https://fsl.fmrib.ox.ac.uk/fsl/docs/registration/newmsm/index.html. The dHCP Deep Learning-based Neonatal Pipeline is available at https://github.com/m-qiang/dhcp-dl-neonatal.
