# Supplementary material for: Motifs of brain cortical folding from birth to adulthood
Source: Nat Commun. 2026 Sep 3;17:10482. doi: 10.1038/s41467-026-77314-5 (PMC13635187; doi:10.1038/s41467-026-77314-5)
Supplement: Supplementary file 1 [file 41467_2026_77314_MOESM10_ESM.pdf]

Corresponding author(s): Dr Yourong Guo, Dr Emma C. Robinson

Last updated by author(s): 29/04/2026

Reporting Summary

Nature Portfolio wishes to improve the reproducibility of the work that we publish. This form provides structure for consistency and transparency in reporting. For further information on Nature Portfolio policies, see our Editorial Policies and the Editorial Policy Checklist.

Statistics

For all statistical analyses, confirm that the following items are present in the figure legend, table legend, main text, or Methods section.

n/a	Confirmed	
☐	☒	The exact sample size (*n*) for each experimental group/condition, given as a discrete number and unit of measurement
☐	☒	A statement on whether measurements were taken from distinct samples or whether the same sample was measured repeatedly
☐	☒	The statistical test(s) used AND whether they are one- or two-sided<br>*Only common tests should be described solely by name; describe more complex techniques in the Methods section.*
☐	☒	A description of all covariates tested
☐	☒	A description of any assumptions or corrections, such as tests of normality and adjustment for multiple comparisons
☐	☒	A full description of the statistical parameters including central tendency (e.g. means) or other basic estimates (e.g. regression coefficient) AND variation (e.g. standard deviation) or associated estimates of uncertainty (e.g. confidence intervals)
☐	☒	For null hypothesis testing, the test statistic (e.g. *F*, *t*, *r*) with confidence intervals, effect sizes, degrees of freedom and *P* value noted<br>*Give P values as exact values whenever suitable.*
☒	☐	For Bayesian analysis, information on the choice of priors and Markov chain Monte Carlo settings
☐	☒	For hierarchical and complex designs, identification of the appropriate level for tests and full reporting of outcomes
☐	☒	Estimates of effect sizes (e.g. Cohen's *d*, Pearson's *r*), indicating how they were calculated

Our web collection on statistics for biologists contains articles on many of the points above.

Software and code

Policy information about availability of computer code

Data collection	No software was used for data collection for this study.
Data analysis	This study utilised the following open software and code:<br>Code for MSM-HT, trained DDR pairwise registration models, and the analysis scripts used in this study are publicly available at https://github.com/Yrong-Guo/Hierarchical-surface-registration. Connectome Workbench version 1.5 is available at https://www.humanconnectome.org/software/connectome-workbench; The DDR registration repository is available at https://github.com/mohamedasuliman/DDR. Surface registration was performed using the newMSM implementation (v1.0), available from the FSL repository https://fsl.fmrib.ox.ac.uk/fsl/docs/registration/newmsm/index.html. The dHCP Deep Learning-based Neonatal Pipeline is available at https://github.com/m-qiang/dhcp-dl-neonatal. HCPpipline level3 analysis was run by FSL version 6.0.3. Twin analyses were conducted using structural equation modelling implemented in the OpenMx package (version 2.22.9) in R 4.5.1.

For manuscripts utilizing custom algorithms or software that are central to the research but not yet described in published literature, software must be made available to editors and reviewers. We strongly encourage code deposition in a community repository (e.g. GitHub). See the Nature Portfolio guidelines for submitting code & software for further information.

Data

Policy information about availability of data

All manuscripts must include a data availability statement. This statement should provide the following information, where applicable:

- Accession codes, unique identifiers, or web links for publicly available datasets
- A description of any restrictions on data availability
- For clinical datasets or third party data, please ensure that the statement adheres to our policy

The adult data used in this study are obtained from the Human Connectome Project Young Adult release (S1200). Preprocessed surfaces were obtained from the HCP ConnectomeDB repository (https://db.humanconnectome.org) under the HCP Open Access Data Use Terms, which require registration and acceptance of a data use agreement prior to download. The neonatal data used in this study (T2w images and DRAW-EM labels) are available from the Developing Human Connectome Project (dHCP) fourth release available through https://biomedia.github.io/dHCP-release-notes/, which requires registration and acceptance of the dHCP open-access data use agreement. The BIBS data used in this study are distinct from the dHCP release and are not deposited in the NDA. These data cannot be shared openly, as participating families did not consent to open data sharing. Access is restricted to the BIBS study team and approved collaborators, governed by the study's ethical approval (London – Dulwich Research Ethics Committee, reference 12/LO/2017); enquiries should be directed to the BIBS study team bibs@kcl.ac.uk and grainne.mcalonan@kcl.ac.uk. After quality control and preprocessing, the final datasets included 1110 adult subjects from HCP-YA, 693 neonatal subjects from dHCP, and 87 subjects from BIBS. MSM-HT motifs can be freely downloaded from https://balsa.wustl.edu/study/ljVNl.
The MSM-HT deformation spheres are publicly available at Zenodo (DOI: 10.5281/zenodo.20507642).

Research involving human participants, their data, or biological material

Policy information about studies with human participants or human data. See also policy information about sex, gender (identity/presentation), and sexual orientation and race, ethnicity and racism.

Reporting on sex and gender	Developing Human Connectome Project (dHCP) and Brain Imaging in Babies (BIBS): 781 neonates were used, of which 648 were term-born (scan age 41.5 ± 1.7 weeks, 297 biologically female); 133 were preterm-born (scan age: 41.3 ± 1.7 weeks, 59 biologically female).<br>Human Connectome Project - Young Adult (HCP-YA): 1110 healthy young adults (605 biological females), who were scanned at 28.8±3.7 years.<br>Sex was not considered in the study design, as the primary objective was to characterise population-level variability in cortical folding across development. The HCP and dHCP datasets include both male and female participants, and our analysis focused on group-level motifs rather than sex-specific differences.
Reporting on race, ethnicity, or other socially relevant groupings	Race and ethnicity were not considered in the study design. The primary goal of the study was to characterise the anatomical variability across development, which is independenct of social factors.
Population characteristics	Developing Human Connectome Project (dHCP) and Brain Imaging in Babies (BIBS): 781 neonates were used, of which 648 were term-born (scan age 41.5 ± 1.7 weeks, 297 biologically female); 133 were preterm-born (scan age: 41.3 ± 1.7 weeks, 59 biologically female).<br>Human Connectome Project - Young Adult (HCP-YA): 1110 healthy young adults (605 biological females), who were scanned at 28.8±3.7 years.
Recruitment	Neonates: Healthy term and preterm neonates were recruited as part of the Developing Human Connectome Project, and BIBS recruited neonates with and without familial history of neurodevelopmental and/or psychiatric conditions such as autism, attention deficit hyperactivity disorder, or mood disorder. They were recruited with the maternity and neonatal units in St. Thomas Hospital, London, UK. All neonates needed to be clinically stable in order to be scanned, which was mostly relevant for preterm neonates. Consequently, preterm neonates tended to be born and scanned later (many of the preterm neonates recruited were moderate-to-late preterm).<br>HCP-YA: These participants were recruited from ~300 families of twins and their non-twin siblings, provided written informed consent prior to imaging and were scanned at Washington University in St. Louis between 2012 and 2015. The inclusion criteria for the HCP-YA was very strict - importantly, preterm birth was an important exclusion criterion.
Ethics oversight	Neonates: dHCP was approved by the London - Riverside Research Ethics Committee of the Health Research Agency (REC:14/LO/1169)<br>HCP-YA: This study was approved by the internal review board of Washington University in St. Louis (IRB #201204036)

Note that full information on the approval of the study protocol must also be provided in the manuscript.

Field-specific reporting

Please select the one below that is the best fit for your research. If you are not sure, read the appropriate sections before making your selection.

☒ Life sciences ☐ Behavioural & social sciences ☐ Ecological, evolutionary & environmental sciences

For a reference copy of the document with all sections, see nature.com/documents/nr-reporting-summary-flat.pdf

Life sciences study design

All studies must disclose on these points even when the disclosure is negative.

Sample size	Neonates (dHCP and BIBS): The sample size is determined by the number of neonates with structural scan acquired at term-equivalent age. Participant were included if no incidental findings were reported; incidental findings considered unlikely to affect the analysis significance were permissible. A total of 781 neonates were included in the study.<br>HCP-YA: Imaging data from 1110 subjects were used, corresponding to all subjects with available structural imaging data in ConnectomeDB. No priori sample size calculations were performed. For each cohort, , all available subjects meeting quality control criteria were included, thereby maximising the the statistic power. The resulting sample sizes are large and consistent with prior cortical surface studies.
Data exclusions	dHCP and BIBS: Data were excluded if any participant had incidental findings with clinical significance e.g. destructive white matter lesions. The could impact surface reconstruction, thus obscuring cortical folding pattern.
Replication	The principal finding – the common cortical folding pattern and the leftward asymmetry was established in in the HCP-YA, and independently replicated in a separate cohort (dHCP+ BIBS). These are independent samples. The study is cross-sectional, with no within subject longitudinal tracking. The findings are additionally consistent with cortical folding patterns and temporal-lobe asymmetry previously reported in postmortem and in vivo imaging studies. All replication attempts were successful.
Randomization	No interventions or treatments were allocated in this study, so randomisation was not required.
Blinding	No interventions or treatments were allocated in this study, so blinding was not required.

Reporting for specific materials, systems and methods

We require information from authors about some types of materials, experimental systems and methods used in many studies. Here, indicate whether each material, system or method listed is relevant to your study. If you are not sure if a list item applies to your research, read the appropriate section before selecting a response.

Materials & experimental systems

n/a	Involved in the study
☒	☐ Antibodies
☒	☐ Eukaryotic cell lines
☒	☐ Palaeontology and archaeology
☒	☐ Animals and other organisms
☒	☐ Clinical data
☒	☐ Dual use research of concern
☒	☐ Plants

Methods

n/a	Involved in the study
☒	☐ ChIP-seq
☒	☐ Flow cytometry
☐	☒ MRI-based neuroimaging

Plants

Seed stocks	*Report on the source of all seed stocks or other plant material used. If applicable, state the seed stock centre and catalogue number. If plant specimens were collected from the field, describe the collection location, date and sampling procedures.*
Novel plant genotypes	*Describe the methods by which all novel plant genotypes were produced. This includes those generated by transgenic approaches, gene editing, chemical/radiation-based mutagenesis and hybridization. For transgenic lines, describe the transformation method, the number of independent lines analyzed and the generation upon which experiments were performed. For gene-edited lines, describe the editor used, the endogenous sequence targeted for editing, the targeting guide RNA sequence (if applicable) and how the editor was applied.*
Authentication	*Describe any authentication procedures for each seed stock used or novel genotype generated. Describe any experiments used to assess the effect of a mutation and, where applicable, how potential secondary effects (e.g. second site T-DNA insertions, mosiacism, off-target gene editing) were examined.*

Magnetic resonance imaging

Experimental design

Design type	cross-sectional design<br>dHCP and BIBS: Only Structural MRI images were analysed.<br>HCP-YA: Structural and task-based functional MRI were analysed.
Design specifications	dHCP and BIBS: All data were acquired in a single scan session<br>HCP-YA: All images were acquired over two sessions

Behavioral performance measures	No behavioural measures were analysed as part of this study

Acquisition

Imaging type(s)	Structrual, task-based functional
Field strength	3 Tesla
Sequence & imaging parameters	HCP-YA: T1w scans were acquired with a 3D MPRAGE sequence (TR=2400 ms, TE=2.14 ms, TI=1000ms, voxel size=0.7 mm isotropic); whereas, T2w scans were acquired with a 3D T2-SPACE sequence (TR=3200, TE=565, voxel size=0.7 mm isotropic). For functional acquisition, resting-state fMRI was acquired with a Gradient-echo EPI sequence (TR=720ms, TE=33.1ms, flip angle=52 degrees, multiband factor 8, 60 minutes for 4 runs, voxel size=2mm isotropic); tfMRI was acquired with the same sequence parameters as resting-state fMRI (7 tasks including Language, Working Memory, Motor, Social Cognition, Gambling, Emotion Processing, Relational Processing, 48 minutes total).<br>dHCP and BIBS: Structural T2w MRI was acquired with a TSE sequence: TR=12000 ms; TE=156 ms; SENSE factors: 2.27 axial and 2.66 sagittal; in-plane resolution 0.8 X 0.8, slice thickness: 1.6 mm, and slice overlap: 0.8 mm.
Area of acquisition	Whole brain

Diffusion MRI ☐ Used ☒ Not used

Preprocessing

Preprocessing software	HCP-YA: Preprocessing performed with HCP minimal preprocessing pipelines (Glasser et al., 2013).<br>dHCP and BIBS: Preprocessing performed with the dHCP neonatal structural pipeline (Makropoulos et al. 2018), then go through a deep learning-based neonatal surface pipeline in (Ma et al. 2024), available at https://github.com/m-qiang/dhcp-dl-neonatal
Normalization	All cortical surface data from HCP-YA and dHCP/BIBS are registered using the Hierarchical surface registration (MSM-HT) describe in the current study. New templates were created in MSM-HT.<br>To compare with the conventional image registration pipeline, subjects from HCP-YA are also registered to a single MSMSulc template.<br>The registration/normalisation process utilised a new version of multimodal surface matching (newMSM), which is a freely available software that performs non-linear surface-based registration using higher order smoothness constraints.
Normalization template	HCP-YA: HCP MSMSulc space<br>dHCP/BIBS: dhcpSym40 space<br>Both adults and neonates underwent MSM-HT, which create new templates for distinct folding clusters.
Noise and artifact removal	No additional artifact removal beyond standard preprocessing was performed. HCP and dHCP pipelines included bias field correction, motion correction.
Volume censoring	N/A

Statistical modeling & inference

Model type and settings	Mann–Whitney U tests were used to compare the degree of lobar asymmetry between lobes<br>Cross-correlation was used to assess the similarity of folding templates across neonates and adults.<br>Chi-square tests were used to assess the association between twin type (MZ vs DZ) and cluster membership, with FDR correction (q=0.05) applied for multiple tests. Odds Ratios were calculated to quantify effect size<br>GLM-based mass univariate model was used for group-level task-fMRI analysis, implemented using FLAMEO (FMRIB's Local Analysis of Mixed Effects) in FSL. This included mixed-effects modeling across subjects for task contrasts (e.g., language, motor, working memory)<br>Two-sample t-tests were used to compare cluster mass metrics between registration methods and tasks
Effect(s) tested	Lobar asymmetry in folding patterns (Left Hemisphere Rate)<br>Similarity of cortical folding patterns between neonates and adults<br>Association between twin zygosity and folding motif similarity<br>Activation for specific task contrasts across individuals<br>Functional alignment quality between registration methods (MSM-HT vs MSM-P)

Specify type of analysis: ☒ Whole brain ☐ ROI-based ☐ Both

Statistic type for inference<br>(See Eklund et al. 2016)	Mann–Whitney U test, Chi-square test, Two-sample t-test, Pearson correlation, GLM-based tfMRI group analysis using FLAMEO (for group average maps)
Correction	FDR correction (q=0.05) for multiple comparisons in asymmetry and heritability analyses; Bonferroni correction for lobar comparisons

Models & analysis

n/a	Involved in the study
☒	☐ Functional and/or effective connectivity
☒	☐ Graph analysis
☒	☐ Multivariate modeling or predictive analysis
