# Supplementary material for: Motifs of brain cortical folding from birth to adulthood
Source: Nat Commun. 2026 Sep 3;17:10482. doi: 10.1038/s41467-026-77314-5 (PMC13635187; doi:10.1038/s41467-026-77314-5)
Supplement: Supplementary file 2 [file 41467_2026_77314_MOESM11_ESM.pdf]

nature portfolio

Peer Review File

Motifs of brain cortical folding from birth to adulthood

Corresponding Author: Dr Yourong Guo

This file contains all reviewer reports in order by version, followed by all author rebuttals in order by version.

Version 0:

Reviewer comments:

Reviewer #1

(Remarks to the Author)
Guo et al. present a new hierarchical surface-registration framework (MSM-HT) to cluster cortical folding patterns into "motifs" and examine their asymmetry, heritability, and relation to function across development. The idea of identifying common folding variants is interesting and could aid neuroanatomical phenotyping. However, several claims require further validation or clarification. Below I address the key points and suggest improvements.

Novelty and validity of MSM-HT: The authors' MSM-HT pipeline combines a deep learning–based surface registration (Deep Discrete Registration, DDR) with agglomerative clustering and MSM-based template generation for each cluster. Many components are adaptations of existing methods: for example, DDR was recently introduced and shown to match classical registration in alignment quality, and MSM (with higher-order smoothness) is well established for cortical alignment. The novelty here lies in applying a hierarchical, cluster-driven registration to capture lobe-wise folding variants. This is a promising approach, but the manuscript should better justify how it differs from prior groupwise frameworks (e.g. other multi-atlas or multi-scale registration methods). It would strengthen the work to demonstrate that MSM-HT yields improved alignment or insight relative to simpler strategies (for example, aligning to a single template). Quantitative validation of registration accuracy (e.g. average feature overlap or landmark correspondence) is not shown; providing such metrics (perhaps by comparing MSM-HT to standard MSM or FreeSurfer registration) would support its validity. In sum, MSM-HT appears technically sound, but the authors should clarify its incremental novelty and empirically validate each stage.

Robustness of motif identification: The clustering of aligned hemispheres into folding "motifs" is central to the claims. It is critical to assess cluster stability and appropriateness. The authors fix the number of clusters (30 per lobe) based on a dendrogram threshold, but the choice seems arbitrary. I would ask if metrics like silhouette scores or cross-validation were considered to justify the cluster count. It would strengthen confidence if the motifs were reproducible across different subsamples or clustering parameters. For example, performing the clustering on random half-samples and checking if similar cluster centers emerge would indicate robustness. Additionally, folding motifs may be sensitive to overall brain size or global morphology: have the authors controlled for differences in hemisphere surface area or volume before clustering? Normalizing sulcal depth or curvature features by global scale (as has been done in gyrification studies) would ensure that clusters reflect genuine pattern differences rather than size. Without such controls, one could not rule out that larger hemispheres disproportionately contribute to certain clusters. Finally, existing literature on sulcal/pit patterns could be cited to contextualize these motifs.

Asymmetry claims (temporal lobe): The authors report strong left–right asymmetry of temporal-lobe folding motifs in both adults and neonates. However, many temporal asymmetries are already known. For instance, the planum temporale typically shows a leftward expansion, and the superior temporal sulcus (STS) tends to be deeper on the right. It is unclear whether the reported "leftward-asymmetric templates" correspond to known landmarks (e.g. left-dominant planum or right-dominant STS). The authors should explicitly compare their asymmetry findings to established effects: e.g. Leroy et al. (2015) showed a characteristic deeper right STS in humans, and Baldeweg et al. (2013) note the pronounced leftward planum temporale asymmetry. Without this context, it is hard to judge novelty. Moreover, statistical evidence should be clearly provided: rather than stating "significant asymmetry," the authors should report effect sizes (e.g. Cohen's d or asymmetry index) and correct for multiple testing across motifs. It would also help to visualize asymmetry (e.g. hemisphere difference maps) in a supplement. An alternative analysis is to flip right-hemisphere maps and rerun clustering under the null of symmetry (as done in Guo 2023); this could directly test whether motif distributions deviate from symmetry.

Structure–function decoupling in association cortex: The authors observe that higher-order (association) regions show a decoupling of folding motif presence from functional network affiliation. This finding is consistent with prior reports that structure–function coupling decreases across a cortical hierarchy. For example, Dong et al. (2024) demonstrated a progressive decoupling from primary to transmodal networks, and Mueller et al. (2013) noted higher anatomical variability in association areas. Thus, the result is plausible. However, it should be presented carefully: a "decoupling" may simply reflect the well-known fact that heteromodal networks exhibit large individual variability in both anatomy and function. If possible, the authors should quantify the coupling (e.g. correlation between motif similarity and functional similarity) and report it in all lobes, rather than only stating a qualitative trend. They should also consider whether the measure of "functional affiliation" (e.g. resting-state network label) is optimal; alternative atlases or continuous gradients might be tested for robustness.

Data processing and alignment pipelines: The manuscript should provide sufficient detail for replication. Key steps (surface extraction, feature generation, registration parameters) must be fully described. For example, it is unclear how surfaces from the different cohorts (HCP adults, dHCP, BIBS neonates) were preprocessed and whether any adjustments (e.g. infant-specific surface refinement) were applied. The DDR training procedure (e.g. network architecture, training data, number of epochs) is only cursorily mentioned – these details should be in Methods or Supplement. Clustering parameters (linkage criteria, distance metric) and MSM regularization settings also need specification. If any code was used, explicit versioning (e.g. FSL/MSM version) should be given. The authors should verify that these pipelines yield consistent results under slight variations (e.g. random initialization), and report any post-hoc QC (e.g. how many hemispheres failed alignment).
Code availability and dataset documentation: The authors mention use of public datasets (HCP, dHCP, BIBS), and they should state the exact data release versions and subject counts analyzed (with relevant accession IDs or DOIs). The manuscript should include a "Code availability" statement with a stable repository link, and similarly a "Data availability" statement detailing how to access the used imaging data (beyond the general project names).
Machine learning reporting checklist: Since the study uses deep learning (DDR) and clustering, the authors should complete the Nature machine learning checklist to ensure all relevant items are addressed (e.g. training/validation split, hyperparameter selection, random seed control). The current manuscript gives no information on how DDR was trained (e.g. was there a held-out set?) or whether results are averaged across multiple runs. Any use of learning-based methods also calls for transparency about hardware and computational resources. I recommend the authors explicitly cite that they have satisfied the checklist and consider including a summary table (or citing the completed checklist PDF) to reassure reviewers that methodological rigor is met.

To further bolster the study, I suggest the following feasible analyses or supplements:
• Surface area normalization: As noted above, recompute folding features after normalizing by total hemisphere area or mean curvature. This will test whether motif differences simply scale with brain size. A supplemental figure could show motif frequencies vs. hemisphere area to rule out confounding.
• Effect sizes for asymmetry: Report Cohen's d or asymmetry indices (with 95% CIs) for the key left–right comparisons of motif prevalence. A table of asymmetry effect sizes per motif and a forest plot could be added to supplement.
• Stability of clustering: Compute a cluster stability metric (e.g. normalized mutual information) by re-running the clustering on bootstrap resamples of subjects. A supplementary plot of clustering consistency vs. number of clusters would inform on robustness.
• Additional figures/tables: A full dendrogram (or hierarchical tree) for each lobe's clustering could be provided as a supplementary figure, to show the distance thresholds used. A table listing each motif with descriptive statistics (mean curvature, sulcal depth, area, asymmetry index) would be informative.
• Sex and age effects: If sample sizes permit, test whether motifs differ by sex or age (within HCP adult range). A quick ANOVA per motif (or regression on age) could reveal whether the reported effects hold after controlling for these factors.
• Genetic model: If possible, run a simple ACE twin model (using e.g. OpenMx or similar) to estimate h^2 for one or two representative motifs. Even a back-of-envelope figure could confirm that the MZ–DZ difference aligns with high genetic influence, as in prior studies
Several limitations and caveats should be acknowledged:
• Sample and population: The HCP sample (young adults) is relatively homogeneous (ethnicity, age) and twins come from one cohort. Findings may not generalize to other populations or age groups. Similarly, the neonate cohorts (dHCP, BIBS) have narrow age windows and may not represent full developmental variability. The paper should note these limits.
• Cross-sectional design: The study is not longitudinal, so "from birth to adulthood" is inferred by comparing different cohorts. Cohort effects or differences in data quality between neonates and adults could affect motif comparisons. This should be mentioned when discussing developmental consistency.
• Method complexity: The hierarchical registration and clustering pipeline is complex; small implementation choices might impact results. For instance, the order of lobe alignment (e.g. frontal first vs. temporal first) could hypothetically bias motif discovery. The authors should make clear what decisions were made and note them as potential sources of bias.
• Over-interpretation of function coupling: The phrase "folding-functional links" in the title suggests a causal or tight relationship, but the analysis is correlational. Given known genetic pleiotropy and many-to-many mapping between structure and function, the authors should avoid implying that folding motifs dictate functional networks. Instead, they might say motifs are associated with certain functional patterns, with residual variability in association cortex.
• Heritability nuances: As noted, "heritability" can be misconstrued. Unless a full genetic model is presented, it would be safer to say motifs show evidence of genetic influence (greater similarity in MZ twins) rather than report a numeric heritability. Also, environmental and measurement factors (e.g. image noise) will reduce apparent heritability.

By tempering conclusions with these points and clearly explaining methodological choices, the authors can present a

balanced, transparent study.
Minor points: The manuscript contains a few minor issues (typos, unclear figure labels, etc.) that should be corrected in revision. For brevity these are not enumerated here but can be provided separately if needed.
Conclusion: Guo et al. tackle an important problem of quantifying cortical folding variability and its genetic/functional correlates. The approach is creative, but the analysis requires stronger validation and clearer reporting to meet field standards of rigor and reproducibility. Addressing the points above – especially clarifying the novelty of MSM-HT, providing statistical detail for asymmetry and heritability claims, and ensuring all methods (and code) are fully documented – will greatly improve the manuscript. The suggested additional analyses and caution on interpretation will also enhance the credibility of the conclusions.

(Remarks on code availability)

Reviewer #2

(Remarks to the Author)
In the current work, Guo et al developed and applied a novel technique to investigate cortical folding in the human cortex, focussing on a young adult and a perinatal sample. In short, they observed different types of folding, with slight patterns of heritability and also asymmetry in temporal regions. Finally they explored whether individuals with similar folding patterns would have similar functional activations.

Overall this is very nice work and a relevant domain for exploration in terms of neurobiology and cognitive implications. I have some questions that may help the paper.

1. Interpretation of difference between neonates and adults is a bit speculative due to potential methodological causes of differences? To what extend are there also alterations or refinement in folding in the first 20 years of life?
2. For the functional analysis,
3. Why were insula and temporal lobe combined in one lobe in the analysis?
4. For the functional mapping, so the idea would be that people with similar folding features would also have similar functional activations?
5. Did authors also test for covariance, e.g. if there is a relation between being similar folding organization in temporal lobe for individual x and y with organization being similar in the frontal lobe? Or are the folding patterns independent per lobe?

(Remarks on code availability)

Version 1:

Reviewer comments:

Reviewer #1

(Remarks to the Author)
I am satisfied that the authors have adequately addressed the substantive reviewer concerns. In particular, they have clarified the methodological novelty of MSM-HT, added quantitative validation against single-template registration, performed clustering stability and robustness analyses, provided additional asymmetry effect-size reporting, expanded the functional coupling analyses, and improved the methodological, code, data availability, and limitations sections.

I do not have remaining substantive concerns and support acceptance of the manuscript. I would only recommend one minor editorial correction before final publication: the abstract should be aligned with the Results regarding neonatal temporal-lobe asymmetry, which is described as a trend that did not reach statistical significance. This wording can be softened without altering the overall conclusions.

(Remarks on code availability)
The authors have provided a public GitHub repository for the MSM-HT framework, including the core surface-registration code, trained DDR registration models, and analysis scripts used for the manuscript.

The README provides a useful overview of the workflow and dependencies, and the manuscript/reporting materials appropriately point readers to the repository.

The code is best described as research/HPC pipeline code rather than a fully packaged, turnkey software release. Some scripts appear to retain hard-coded paths, local compute assumptions, and fixed parameters that may require adaptation by external users. These issues do not, in my view, preclude acceptance, because the central methodological code and trained models are publicly available. However, before publication, I would encourage the authors to improve long-term

reproducibility by archiving a fixed release, adding an environment file or container specification, and providing a minimal example command or test dataset illustrating expected inputs and outputs.

Overall, the code availability is adequate for publication, with minor recommended improvements for portability and reproducibility.

Reviewer #2

(Remarks to the Author)
I thank the authors for their detailed replies, and have no further comments.

(Remarks on code availability)
I have visually inspected the code and it looks usable.

Open Access This Peer Review File is licensed under a Creative Commons Attribution 4.0 International License, which permits use, sharing, adaptation, distribution and reproduction in any medium or format, as long as you give appropriate credit to the original author(s) and the source, provide a link to the Creative Commons license, and indicate if changes were made.
In cases where reviewers are anonymous, credit should be given to 'Anonymous Referee' and the source.
The images or other third party material in this Peer Review File are included in the article's Creative Commons license, unless indicated otherwise in a credit line to the material. If material is not included in the article's Creative Commons license and your intended use is not permitted by statutory regulation or exceeds the permitted use, you will need to obtain permission directly from the copyright holder.
To view a copy of this license, visit https://creativecommons.org/licenses/by/4.0/

Revision for "Motifs of brain cortical folding from birth to adulthood: structural asymmetry and folding-functional links"

The response is organised in a point-by-point format. Reviewer comments are labelled sequentially in green (e.g. ***C1**, **C2***) followed by our responses in black (e.g. **R1, R2**). Corresponding changes in the manuscript are highlighted in pink and referenced by page and line numbers in the revised manuscript. We also provide a version of the manuscript with tracked changes, with the machine learning checklist for further information.

Summary

Reviewer 1

C1. Novelty and validity of MSM-HT

C2. Robustness of motif identification

C3. Asymmetry claims (temporal lobe)

C4. Structure–function decoupling in association cortex

C5. Data processing and alignment pipelines

C6. Code availability and dataset documentation

C7. Surface area normalization

C8. Effect sizes for asymmetry

C9. Stability of clustering

C10. Additional figures/tables

C11. Sex and age effects

C12. Genetic model

C13. Limitations and caveats

Reviewer 2

C14. Interpretation of difference between neonates and adults

C16. Insula and temporal lobe combined in the analysis

C17. Interpretation of the experiment of functional mapping

C18. Covariance of cortical folding across lobes

Reviewer Comments

Reviewer #1 (Remarks to the Author):

Guo et al. present a new hierarchical surface-registration framework (MSM-HT) to cluster cortical folding patterns into "motifs" and examine their asymmetry, heritability, and relation to function across development. The idea of identifying common folding variants is interesting and could aid neuroanatomical phenotyping. However, several claims require further validation or clarification. Below I address the key points and suggest improvements.

C1. Novelty and validity of MSM-HT: *The authors' MSM-HT pipeline combines a deep learning–based surface registration (Deep Discrete Registration, DDR) with agglomerative clustering and MSM-based template generation for each cluster. Many components are adaptations of existing methods: for example, DDR was recently introduced and shown to match classical registration in alignment quality, and MSM (with higher-order smoothness) is well established for cortical alignment. The novelty here lies in applying a hierarchical, cluster-driven registration to capture lobe-wise folding variants. This is a promising approach, but the manuscript should better justify how it differs from prior groupwise frameworks (e.g. other multi-atlas or multi-scale registration methods). It would strengthen the work to demonstrate that MSM-HT yields improved alignment or insight relative to simpler strategies (for example, aligning to a single template). Quantitative validation of registration accuracy (e.g. average feature overlap or landmark correspondence) is not shown; providing such metrics (perhaps by comparing MSM-HT to standard MSM or FreeSurfer registration) would support its validity. In sum, MSM-HT appears technically sound, but the authors should clarify its incremental novelty and empirically validate each stage.*

R1.1. We thank the reviewer for these helpful suggestions and agree that we could have done more to justify the benefits of MSM-HT relative to prior groupwise frameworks .

Previous work has proposed hierarchical or multi-atlas frameworks to account for anatomical variability, either by clustering images (Wang et al., 2010), or by selecting and fusing labels from similar atlases (Heckemann et al., 2006, Gousias et al., 2013).

In multi-altas segmentation, a relatively small set of manually labelled brains is registered to a target image, and labels are fused in a weighted manner, based on similarity. This method implicitly assumes that anatomical variability can be adequately represented by a limited number of fixed discrete templates. However, our result reveals a substantially richer and more structured variability in cortical folding patterns, particularly at the lobe level. This raises the question of whether a small fixed set of template brains is sufficient to represent the full space of folding variability. Therefore, a hierarchical, cluster-driven approach provide a data-driven representation of variability, allowing distinct folding motifs to be modelled explicitly rather than approximated by a limited template set.

Hierarchical groupwise registration (e.g., Wang et al., 2010) are conceptually related to our approach. But are typically implemented in the volumetric domain. While volumetric registration has proven effective for aligning global anatomical structures, it relies on intensity-based correspondences and volumetric diffeomorphic constraints regularised in Euclidean space. Such regularisation does not respect the intrinsic geometry of the cortical sheet, where distances are better characterised along the surface. As a result volumetric approaches often struggle to accurately align highly convoluted cortical surfaces. In contrast, our method used the surface-based approach, where registration is performed with respect to geodesic distances. This enables more precise alignment of folding features. Furthermore, by combining surface-based registration with hierarchical clustering, our framework explicitly models lobe-wise variability in folding patterns. The use of a deep learning-based pairwise registration also made this approach computationally feasible.

We add this context in the related text in the manuscript:

Pages 2-3, Lines 67-73

"A key assumption underlying human neuroimaging studies is that spatial normalisation can map individual brains to a single population-average space, in which common structures (e.g. sulci and gyri) overlap; this then allows for straightforward comparison and modelling of these features during downstream analyses. Since optimisation of these mappings is ill-posed, image registration algorithms are typically regularised with diffeomorphic constraints, which enforce smooth, invertible transforms that allow no folding or tearing of the deformation grid. This contradicts the body of evidence, described above, which shows that equivalent sulci can appear continuous in some individuals, but branched or split in others. As a result, enforcing globally consistent correspondence may obscure, rather than capture, the true diversity of folding patterns. To address this, previous approaches have employed strategies such as multi-atlas segmentation (Heckemann et al., 2006), or group-wise registration (Wang et al., 2010; Ying et al., 2014), in which a fixed number of templates may be defined to capture shape variability; however, these have typically been formulated in the volumetric domain, where diffeomorphic regularisation is applied in Euclidean space, and therefore do not fully respect the intrinsic geometry of the cortical sheet. Consequently, they may be insufficient to capture the scale and structured variability of cortical folding across individuals.

In this paper, we explore a hierarchical image registration framework (MSM-HT), which leverages learning-based surface-registration methods (Suliman et al., 2022) to perform fast diffeomorphic-alignment between all pairs of hemispheres, from a large cohort. We then assess similarity between cortical folding patterns by how easily they can be diffeomorphically aligned; this then allows common folding patterns to be clustered and co-aligned using multimodal surface matching (MSM) (Robinson et al., 2014; Robinson et al., 2018, Besenczi et al., 2024) (see Hierarchical MSM registration)"

R1.2. With respect to quantitative evaluation of how MSM-HT performs relative to alignment to a single population template (MSM-P), we sought to address this within the original manuscript through evaluation of the standard deviation (STD) of the cortical features (curvature) after alignment for representative folding motifs (**manuscript Figure 5**). These results showed reduced inter-subject uncertainty for MSM-HT, indicating that individuals within a cluster share more consistent folding patterns. As a result, MSM-HT produces sharper and more variable folding motifs (mean maps) than seen for MSM-P, particularly in regions with high folding variability (e.g., the middle frontal gyrus).

Following the reviewer's suggestion, we have now added a quantitative evaluation of alignment accuracy.

Supplementary Information Page 2, Lines 38-54

"To quantify how MSM-HT performs relative to alignment to a single population template (MSM-P), we compute, for each cluster, vertex-wise STD maps of sulcal depth across subjects after registration. To focus on regions that are most challenging to align, a reference variability map was constructed by taking the per-vertex maximum variability observed across MSM-HT and MSM-P, thereby capturing vertices that exhibit high residual variability under either registration. Vertices were then ranked according to this reference variability, and subsets corresponding to the top 70%, 60%, 50%, 40%, 30%, 20%, 10%, and 1% most variable vertices were analysed. Within each subset, the mean residual variability was calculated separately for MSM-HT and MSM-P, producing threshold–response curves describing how alignment precision changes when progressively focusing on highly variable cortical regions (Fig.S6, **Figure C1**). Across all clusters, MSM-HT consistently exhibits lower residual variability than MSM-P, with the largest improvements observed in the most variable regions. This quantitative result supports the qualitative

observations in the main paper Fig.5, and indicates that hierarchical cluster-specific registration improves the alignment of heterogeneous folding patterns compared with conventional single-template approaches.

Areal distortion for each vertex is defined as the average of the distortion for all triangles adjacent to the vertex p, $1/N_p \Sigma_i log_2(AD_i/AO_i)$ where AO_i and AD_i are the area of triangle i before and after distortion, and N_p is the number of triangle adjacent to vertex p. To ensure a fair comparison, we controlled for area distortion – adjusting regularisation terms (i.e. lambda, see main paper) until the 95th percentile of area distortions matched between MSM-HT and MSM-P. This constraint was applied consistently across all analyses comparing MSM-HT and MSM-P (The distribution of area distortion is shown in Fig.S7)."

In addition, we evaluated cluster coherence by comparing within-cluster and between-cluster curvature similarity (**Table C4, R4.1**). Within-cluster similarity is substantially higher than between-cluster similarity, further supporting that MSM-HT captures consistent motif-specific folding organisation.

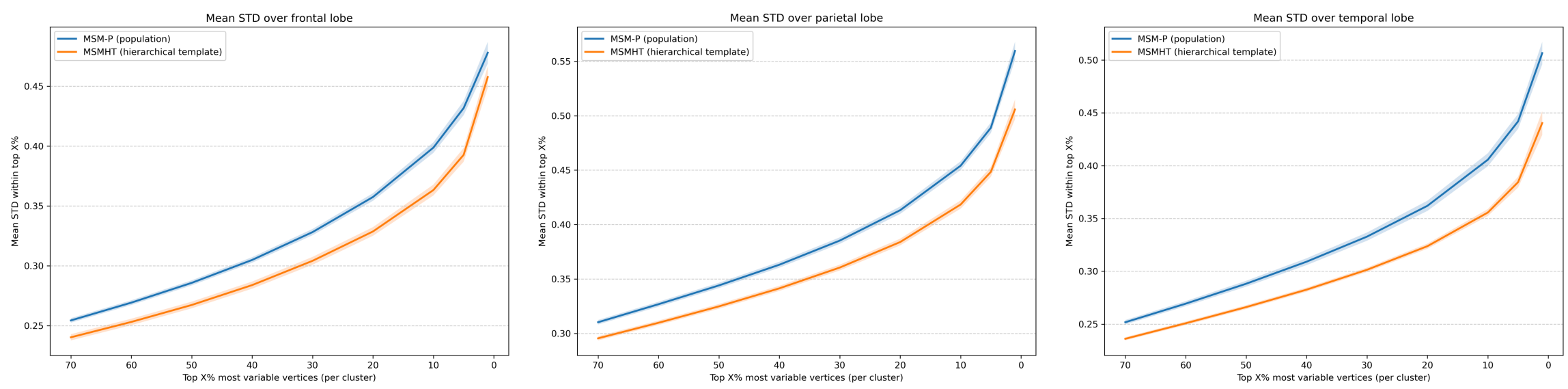

Figure C1 (Fig.S6)

↩ **Back to Summary**

C2.1. Robustness of motif identification: *The clustering of aligned hemispheres into folding "motifs" is central to the claims. It is critical to assess cluster stability and appropriateness. The authors fix the number of clusters (30 per lobe) based on a dendrogram threshold, but the choice seems arbitrary. I would ask if metrics like silhouette scores or cross-validation were considered to justify the cluster count. It would strengthen confidence if the motifs were reproducible across different subsamples or clustering parameters. For example, performing the clustering on random half-samples and checking if similar cluster centers emerge would indicate robustness.*

R2.1. We thank the reviewer for the insightful suggestion to evaluate clustering stability. To assess the robustness of motif identification and the choice of cluster number, we performed a bootstrap-based stability analysis. This also addresses **Comment C9**.

For each lobe, hierarchical clustering was performed using the same similarity matrix and complete linkage parameters described in the manuscript. The resulting dendrogram was then evaluated across a range of cluster numbers (K = 5–40). To assess stability, we repeatedly subsampled 90% of hemispheres without replacement and recomputed the clustering using identical parameters. For each subsample, cluster assignment was computed with those obtained from the full dataset, using only the hemispheres present in both sets. Agreement was quantified using the adjusted mutual information (AMI), which accounts for chance agreement and is invariant to label permutation. This procedure was repeated 100 times for each value of K, yielding a mean and standard deviation of AMI.

Figure C2 left shows clustering consistency as a function of the number of clusters. Across lobes, clustering stability increased gradually with the number of clusters. This indicates that optimising for stability would lead to a trivial solution where each sample forms its own cluster.

To further assess over-fragmentation, we quantified the number of clusters containing very few hemispheres (<5) (**Figure C2 right**). At larger K values, the number of such small clusters increased rapidly, particularly beyond K ≈ 25-30, indicating fragmentation into low-population clusters. Taken together, these results reveal a trade-off between clustering stability and fragmentation. We therefore selected K=30 as a representative operating point that balances reproducibility and interpretability while limiting over-fragmentation.

More fundamentally, while surface registration aims to map all brains into a common coordinate system, perfect alignment of cortical folding patterns within a single diffeomorphic space is limited by topological variability (e.g. sulcal branching or interruptions), which cannot be resolved using shape information alone. Clustering therefore provides an intermediate representation that captures dominant modes of folding variation prior to alignment.

In this framework, the number of clusters determines how much distinct folding variability are treated as separately alignable under diffeomorphic constraints. Increasing K improves within-cluster alignment by separating unalignable folding patterns, but at higher K begins to partition continuous variation, leading to over-fragmentation.

Future work may further extend the MSM-HT framework by using functional information to guide alignment, or by modelling folding variation in a continuous latent space (e.g. Guillon et al, 2024 and Gaudin et al, 2024). These experiments are added into **Supplementary Methods – S1.3 Justification of clustering approach (Supplementary Information Page 3, Lines 78-104)**

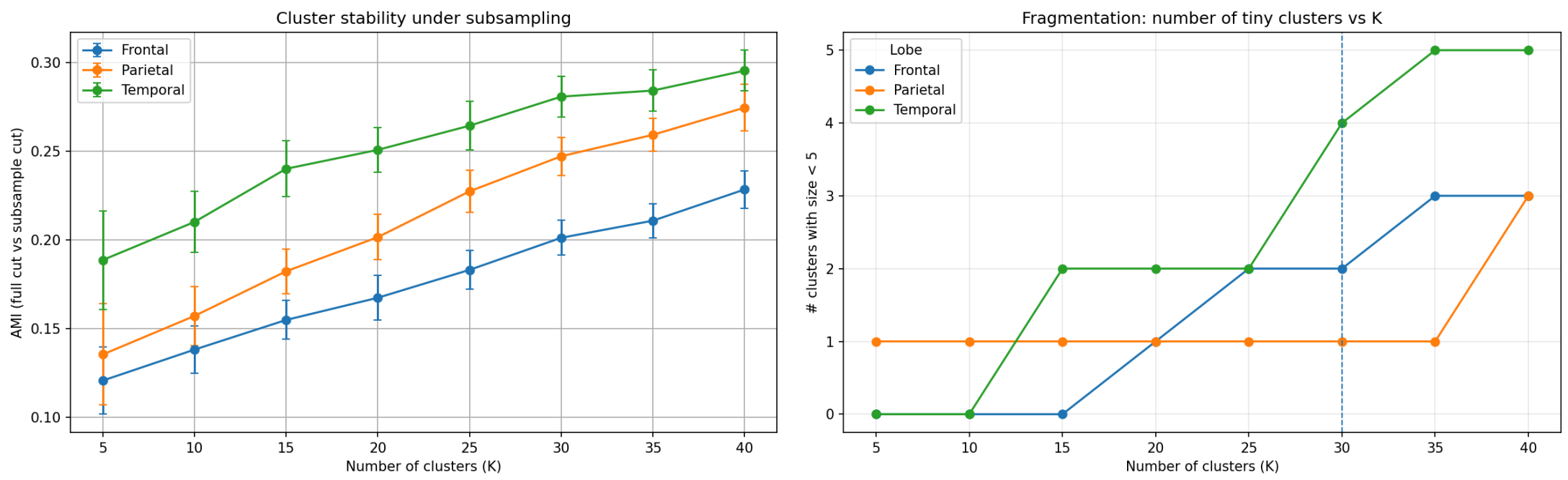

Figure C2. Clustering consistency vs. the number of clusters.

C2.2. *Additionally, folding motifs may be sensitive to overall brain size or global morphology: have the authors controlled for differences in hemisphere surface area or volume before clustering? Normalizing sulcal depth or curvature features by global scale (as has been done in gyrification studies) would ensure that clusters reflect genuine pattern differences rather than size. Without such controls, one could not rule out that larger hemispheres disproportionately contribute to certain clusters.*

R2.2. We thank the reviewer for highlighting the potential influence of global brain size on folding-based clustering. Our response also addresses **Comment C7**.

To address the reviewer's concern, we quantified the relationship between cluster membership and lobar surface area via a Kruskal-Wallis test. Although the tests were statistically significant due to the large sample size, effect sizes were small overall ($\varepsilon^2 \leq 0.093$ in adults and $\varepsilon^2 \leq 0.064$ in neonates, see **Table C1**, **C3**), indicating that surface area explains only a limited proportion of variance in cluster membership. Moreover, surface area distributions show substantial overlap across motifs (**Figure C3.A**, **Figure C3.B**), suggesting that clustering is not primarily driven by global brain size differences.

It is important to note that surface area and folding complexity are inherently related due to coupling between cortical expansion and folding – where this is reflected by strong correlations between surface area and gyrification for each lobe ($r = 0.62$-0.67, $p<0.0001$). As such modest differences in surface area across motifs may reflect genuine variation in folding complexity rather than a confounding effect of brain size.

Consistent with that interpretation, effect sizes relating folding motifs to surface area were smaller in neonates than in adults, despite much larger variability in brain size during early development (**Table C3**). That brain size has even less of an effect here, suggests that motif identification reflects genuine folding variation rather than simple scaling with brain size.

The Kruskal–Wallis statistics and corresponding effect sizes are reported below.

For adults:

Table C1. Kruskal–Wallis tests assessing differences in lobar surface area across folding motif clusters. All tests were significant ($p < 0.001$); however, effect sizes (ε^2) were small, indicating that global surface area explains only a limited fraction of variance in cluster membership.

Lobe	Kruskal–Wallis H	p-value	Effect size (ε^2)
Frontal	232.68	< 0.001	0.093
Parietal	121.50	< 0.001	0.042
Temporal	185.57	< 0.001	0.072

Table C2. Kruskal–Wallis tests assessing differences in hemispheric brain volume across folding motif clusters. All tests were significant ($p < 0.001$); however, effect sizes (ε^2) were small, indicating that brain volumes explains only a limited fraction of variance in cluster membership.

Lobe	Kruskal–Wallis H	p-value	Effect size (ε^2)
Frontal	107.43	< 0.001	0.036
Parietal	104.02	< 0.001	0.034
Temporal	132.14	< 0.001	0.047

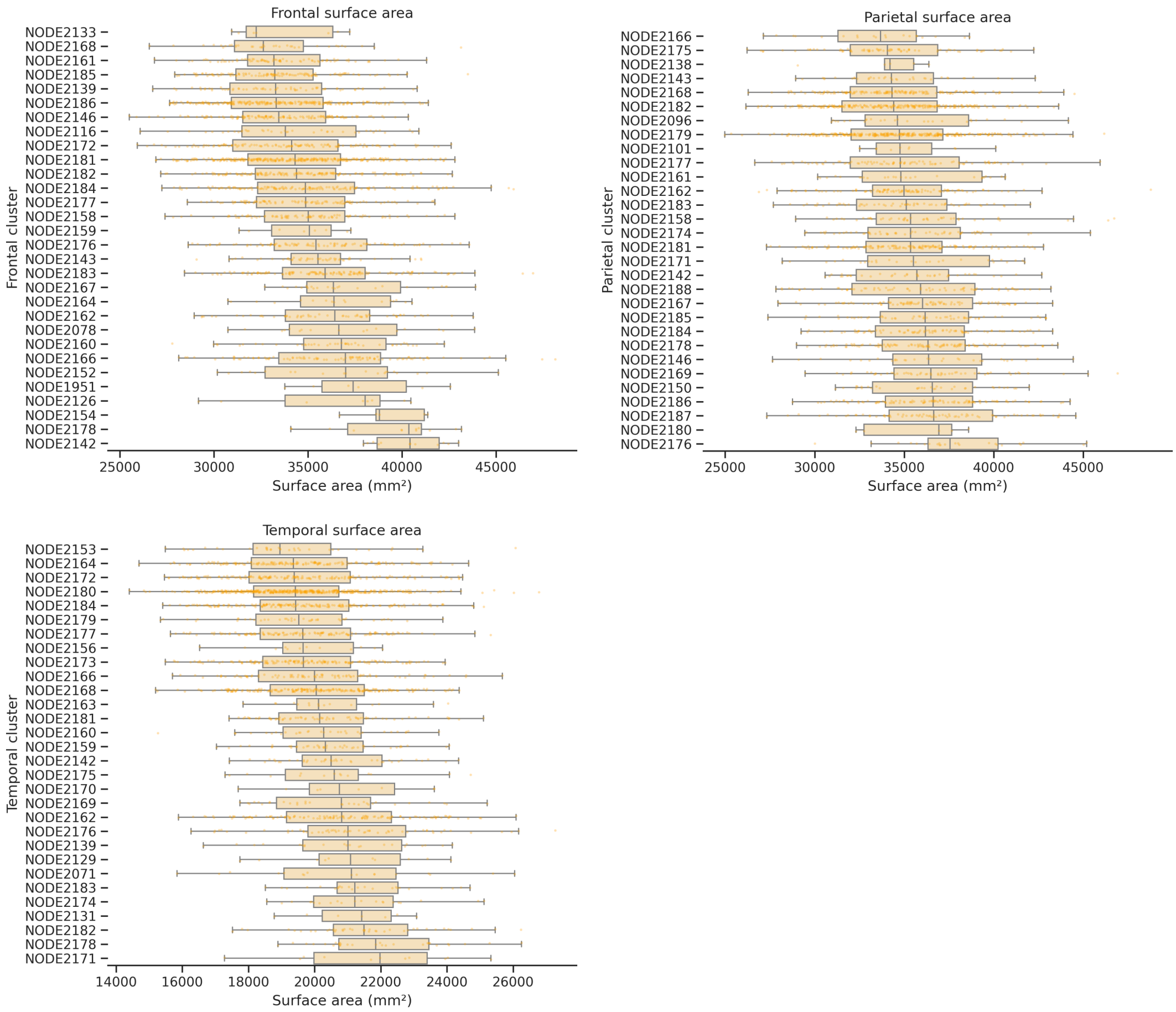

Figure C3.A Box plot for surface area distribution in each HCP-YA derived cluster. The order is arranged by median value.

For Neonates:

Table C3. Kruskal–Wallis tests assessing differences in lobar surface area across folding motif clusters. All tests were significant ($p < 0.001$); however, effect sizes (ε^2) were small, indicating that global surface area explains only a limited fraction of variance in cluster membership.

Lobe	Kruskal–Wallis H	p-value	Effect size (ε^2)
Frontal	87.33	< 0.001	0.038

Parietal	113.08	< 0.001	0.055
Temporal	126.43	< 0.001	0.064

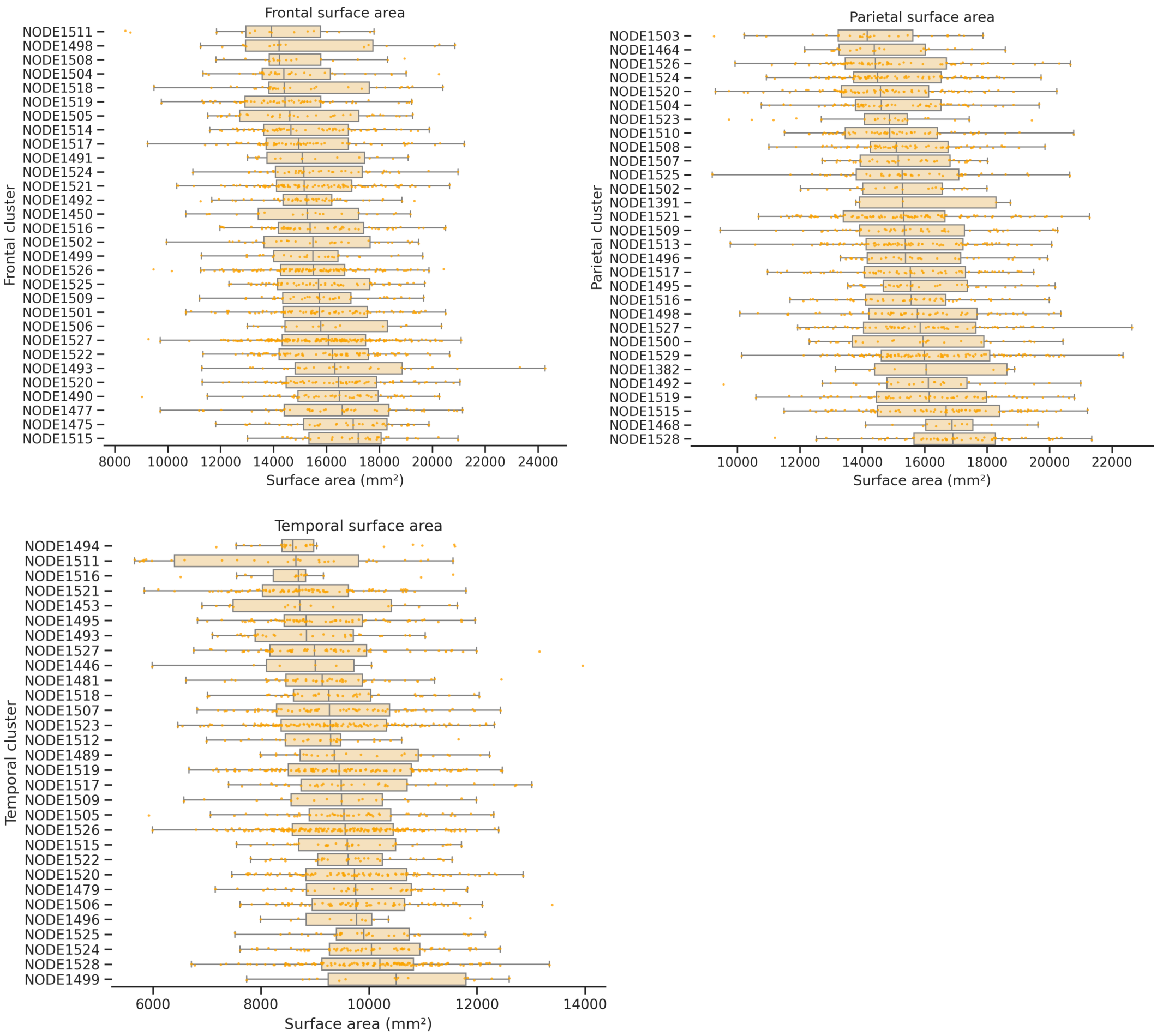

Figure C3.B Box plot for surface area distribution in each neonates derived cluster. The order is arranged by median value.

C2.3. *Finally, existing literature on sulcal/pit patterns could be cited to contextualize these motifs.*

Sulcal roots (Régis et al., 2005) and deep sulcal landmarks (Im et al., 2009; Im et al., 2019) are thought to represent early-forming cortical folding features that remain relatively stable across individuals throughout development. These sulcal roots/pits may be separated by plis de passage that interrupt major folds, and can be considered as fundamental building blocks of complex cortical folding patterns. In this context, the motifs identified in our study can be interpreted as higher-order configurations of these stable landmarks, capturing how these elements are arranged and combined into complex patterns across individuals.

We specifically expand our discussion in the following way:

Page 9, Lines 178-182

"Patterns of cortical folding result from the long process of structural and functional cortical development and offer a window into misdevelopment. Our study was motivated by a need to uncover a comprehensive baseline of "typical" cortical folding, to increase our sensitivity to detect morphological motifs of behaviour and disease. To achieve this, we parsed heterogeneous patterns of cortical folding into a discrete set of typical templates for each anatomical lobe. Previous studies have proposed that cortical folding patterns are organised around reproducible elementary units, including sulcal roots (Régis et al., 2005) and deep sulcal landmarks (Im et al., 2009, Im et al., 2019), which remain relatively stable across individuals despite substantial variability in the overall folding. In this context, our motifs can be interpreted as the higher-order configuration of these stable landmarks, capturing how these elements are arranged and combined into complex patterns across individuals. Our motifs aligned with variants manually defined by (Ono et al., 1990), and our results uncovered both common cortical variants (i.e. seen in at least 5% of the sample) and known, but rarer, variants (such as the single-segment PreCS in cluster F2126). This anatomical foundation provides a novel framework for exploring structural brain organisation at a larger scale."

↩ **Back to Summary**

__C3. Asymmetry claims (temporal lobe):__ The authors report strong left–right asymmetry of temporal-lobe folding motifs in both adults and neonates. However, many temporal asymmetries are already known. For instance, the planum temporale typically shows a leftward expansion, and the superior temporal sulcus (STS) tends to be deeper on the right. It is unclear whether the reported "leftward-asymmetric templates" correspond to known landmarks (e.g. left-dominant planum or right-dominant STS). The authors should explicitly compare their asymmetry findings to established effects: e.g. Leroy et al. (2015) showed a characteristic deeper right STS in humans, and Baldeweg et al. (2013) note the pronounced leftward planum temporale asymmetry. Without this context, it is hard to judge novelty. Moreover, statistical evidence should be clearly provided: rather than stating "significant asymmetry," the authors should report effect sizes (e.g. Cohen's d or asymmetry index) and correct for multiple testing across motifs. It would also help to visualize asymmetry (e.g. hemisphere difference maps) in a supplement. An alternative analysis is to flip right-hemisphere maps and rerun clustering under the null of symmetry (as done in Guo 2023); this could directly test whether motif distributions deviate from symmetry.

R3. We thank the reviewer for this helpful suggestion regarding the interpretation and statistical reporting of temporal-lobe asymmetry. This Response also addresses **Comment C8**.

First regarding the methodological point, we have followed the same approach as described in Guo et al., 2023. In that work, the right hemispheres were mirror-flipped to the left and rotated to the same template space as left hemispheres before clustering. This tests against the null hypothesis that the hemispheres are perfectly symmetrical.

Second, following the reviewers' suggestion, we now provide the explicit statistical evidence of asymmetry. We calculated an Asymmetry index (AI) of motif prevalence, defined as (L-R)/(L+R), where L and R denote the number of left and right hemispheres assigned to each cluster. The AI ranged from -1 (rightward) to 1 (leftward). False discovery rate (FDR) correction applied across motifs. Because the AI is derived from discrete hemisphere counts, 95% confidence intervals were computed using the Wilson score interval. As requested, asymmetry indices and their confidence intervals are summarised in **Table C6** **(Supplementry Table S4),** and a forest plot summarising motif level asymmetry effects in **Figure C4**. Together with the **main Figure 2**, these analyses highlight a stronger leftward asymmetry in temporal folding motifs compared with other cortical regions.

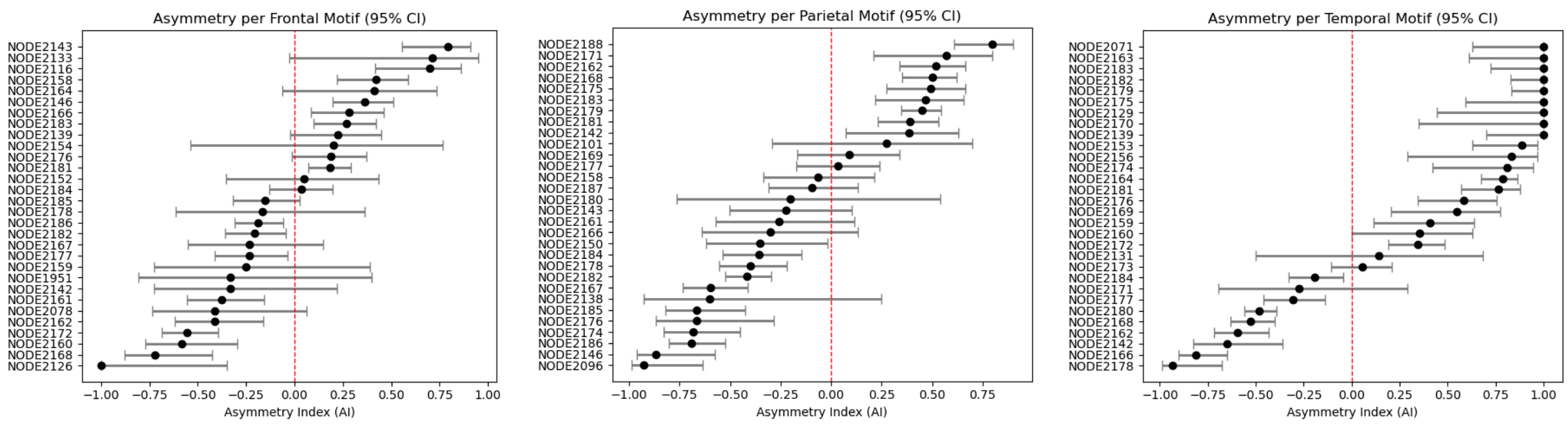

Figure C4

Third, we agree that our finding should be explicitly contextualised within the established literature on temporal lobe asymmetry. Importantly, our results are consistent with previous studies (Leroy et al., 2015; Pahs et al., 2013; Geschwind et al., 1968; Le Guen et al., 2018; Im et al., 2009), but also provide a novel contribution by extending beyond prior findings derived from scalar measures (e.g. sulcal depth, volume, number of interruptions) to the spatial organisation of cortical folding motifs. Such scalar measurements describe local geometric properties but do not capture how folds are arranged across the cortical surface. This limitation is partially due to the reliance on

alignment to a single population average template, which assumes pointwise correspondence across subjects, and can obscure variability in folding configurations, particularly in regions where sulcal patterns do not align consistently.

In contrast, our approach characterises the cortical folding variability with a set of representative motifs, which captures how folding patterns are organised across individuals and how these configurations differ between hemispheres. This provides a more comprehensive and interpretable representation of structural asymmetry, and offers a framework for integrating other modalities to better understand the mechanisms behind these asymmetries.

We have now clarified this comparison in result and discussion in the revised manuscript.

Page 12, lines 195-209

"Cortical asymmetry has long been recognised, particularly in the planum temporale, which is typically larger in the left hemisphere and linked to language processing (Geschwind et al., 1968; Pahs et al., 2013). More recent work has identified asymmetries in the STS, including a deeper right STS and more frequent interruptions in the left, suggesting genetically constrained features related to language networks (Leroy et al., 2015; Le Guen et al.,2018; Le Guen et al., 2020). Beyond previous studies that characterised asymmetry using scalar measures (e.g. sulcal depth, regional size) following alignment to a single population-average template (Leroy et al., 2015; Pahs et al., 2013), or reported greater interruptions and sulcal pits in the left STS (Le Guen et al.,2018; Im et al., 2009), our approach captures asymmetry in the spatial organisation of cortical folding patterns. Such scalar summaries describe local geometric properties but do not capture how folds are arranged across the cortical surface, particularly in regions with inconsistent sulcal alignment. In contrast, our clustering-based framework characterises asymmetry at the level of folding motif organisation. Using this approach, we observed greater folding variability in the left temporal lobe, particularly in the STS, where leftward-asymmetric variants exhibit more branching and interruptions by *plis de passage*. This asymmetry is evident in both neonates and adults and extends previous findings by showing how such differences manifest in the organisation and prevalence of distinct folding motifs. The increased structural complexity on the left may provide a structural basis for the lateralisation of language processing, as supported by Bodin et al., 2021, who found dense bundles of U-shaped fibres related to the positions of the *plis de passage*. In line with this, we discovered significantly higher normalised temporal lobe surface areas in the left hemisphere compared to the right in adult data, suggesting more structural capacity related to language processing."

↩ **Back to Summary**

C4. Structure–function decoupling in association cortex: *The authors observe that higher-order (association) regions show a decoupling of folding motif presence from functional network affiliation. This finding is consistent with prior reports that structure–function coupling decreases across a cortical hierarchy. For example, Dong et al. (2024) demonstrated a progressive decoupling from primary to transmodal networks, and Mueller et al. (2013) noted higher anatomical variability in association areas. Thus, the result is plausible.*
However, it should be presented carefully: a "decoupling" may simply reflect the well-known fact that heteromodal networks exhibit large individual variability in both anatomy and function. If possible, the authors should quantify the coupling (e.g. correlation between motif similarity and functional similarity) and report it in all lobes, rather than only stating a qualitative trend. They should also consider whether the measure of "functional affiliation" (e.g. resting-state network label) is optimal; alternative atlases or continuous gradients might be tested for robustness.

R4.1. We thank the reviewer for this insightful suggestion. We agree that apparent "decoupling" between cortical folding and function may reflect the well-known observation that heteromodal regions exhibit substantial inter-individual variability in both anatomy and functional organisation. To address this issue more directly, we have now performed additional quantitative analyses to evaluate structure-function coupling.

First, we compared the within- and between-cluster structural and functional similarity across subjects. Folding similarity was defined as the Pearson correlation between curvature maps of each pair of subjects within the lobe, and functional similarity as the Pearson correlation between concatenated task-fMRI contrast maps for each pair of subjects within the same lobe. Within-cluster folding similarity is substantially larger than the between-cluster similarities across all regions (frontal: Cohen's d = 1.54; parietal: d = 1.67; temporal: d = 1.41, **Table C4**, **Figure C5** left), confirming that the MSM-HT clusters capture strongly coherent folding patterns. In contrast, functional similarity showed only minimal within-cluster increases (frontal: d = 0.054; parietal:d = 0.06; temporal: Cohen's d = 0.09, **Table C4**, **Figure C5** right), indicating limited functional differentiation between folding motifs. As shown in **Figure C5**, functional similarity exhibits a broad distribution across subject pairs, indicating heterogeneous inter-subject similarity. However, this variation is not structured to folding-defined clusters, as evidenced by the minimal separation between within- and between-cluster distributions.

Table C4. Mean Pearson correlation coefficient within and between clusters for folding and function

**Lobe**	**Folding**			**Function**		
	Within	Between	Cohen's d	Within	Between	Cohen's d
Frontal	0.494	0.415	1.54	0.227	0.224	0.054
Parietal	0.461	0.378	1.67	0.388	0.384	0.060
Temporal	0.539	0.454	1.41	0.302	0.295	0.091

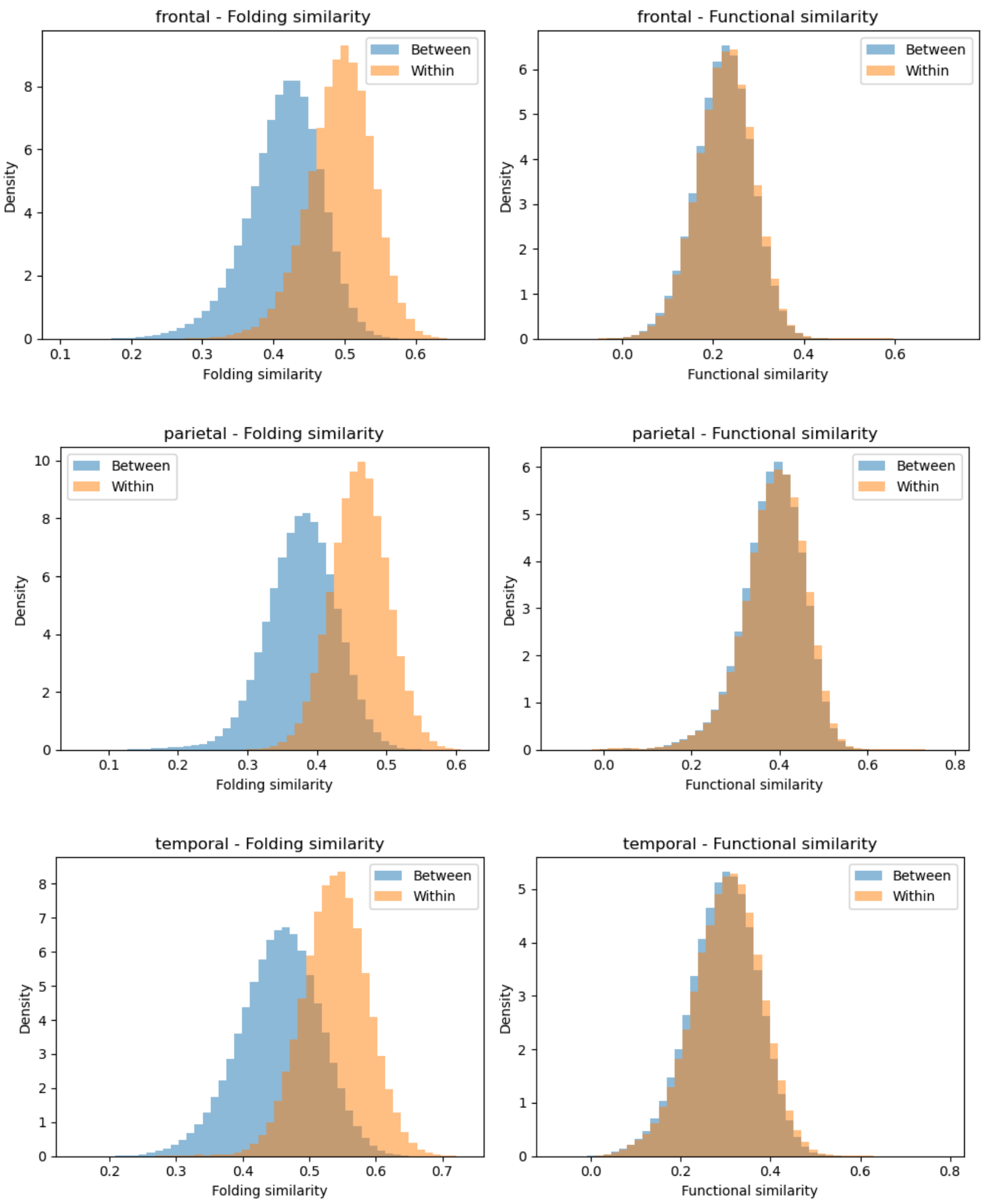

Figure C5. Distribution of Pearson correlation coefficients within and between clusters for folding and function.

Second, following the reviewer's suggestion, we quantified continuous structure-function coupling by computing the Spearman correlation between the upper-triangular entries of the subject-subject folding and function similarity matrices for each lobe. Statistical significance was assessed using a permutation-based Mantel test (5000 permutations), in which subject labels were randomly permuted (simultaneously shuffling rows and columns of the functional similarity matrix) to generate a null distribution of coupling values. Across lobes, the coupling coefficient ranged from ρ=0.068 to 0.104 (all permutations p<0.001), corresponding to less than ~1% variance explained (**Table C5**). Importantly, as demonstrated above, this weak coupling is unlikely to reflect a lack of inter-subject variability in functional organisation. Although statistically significant due to large sample size, these small effect sizes indicate that folding similarity accounts for only a limited proportion of variation in functional similarity.

Table C5. Coupling coefficient of structure and function

Lobe	ρ (Spearman)	R^2 (%)	p (Mantel)
Frontal	0.094	0.89	<0.001
Parietal	0.104	1.08	<0.001
Temporal	0.068	0.46	<0.001

Together, these analyses suggest that while folding motifs capture robust structural organisation, their correspondence with functional similarity is weak. This supports our interpretation that cortical folding motifs and functional network organisation are only loosely coupled, particularly in higher-order cortical regions.

These analyses have been added to the **Supplementary Results S2.3 (Page 5, Lines 162-179)** and summarised in **Supplementary Tables S2, S3**. We reference them in the **Revised manuscript:**

Page 18, Lines 436-437

"… The significance of the difference between MSM-HT and MSM-P were evaluated using two-sample t-tests, followed by FDR of 0.05 for multiple comparisons. Additional analyses quantifying folding-function coupling, including within/between-cluster similarity comparisons and Mantel correlation tests between folding and functional similarity matrices, are reported in Supplementary Results S2.3."

Page 9, lines 163-166

"This could be explained by the fact that the placement of architectonic areas is not constrained by morphological landmarks (sulci and gyri) (21). Quantitative analyses confirmed that folding motifs show stronger structural coherence within clusters (Cohen's d = 1.41-1.67 across lobes), whereas functional similarity shows minimal differentiation between clusters across lobes (Cohen's d = 0.05-0.09; Supplementary Table S3). The coupling coefficient between structural and functional similarities ranged from $\rho = 0.068$ to 0.104 (all permutations $p < 0.001$; Supplementary Table S2)."

In the **Discussion Section**, we change the following text:

Page 12, lines 226-232, we add:

In these areas, folding is much more irregular, and their correspondence with functional organisation is much weaker. Supplementary analyses further quantified this relationship, showing that the correlation between folding similarity and functional similarity across individuals is small ($\rho = 0.068$–0.104 across lobes), indicating that folding patterns explain only a limited proportion of inter-individual variability in functional organisation. One possible interpretation for these results is that cortical folding aligns more with primary functional regions because early-forming folds are under stronger genetic control, whereas later-forming folds in association cortex are influenced by more variable biomechanical and environmental factors. In line with previous heritability research in HCP (4), our study found a stable genetic influence within lobes in the adult brain, indicated by MZ twins being significantly more likely to fall into the same folding clusters than the DZ twins.

R4.2. We appreciate the reviewer's suggestion regarding the definition of functional affiliation. In the revised manuscript, we evaluated the folding–function relationship using multiple complementary functional measures. First, we examined task-based fMRI activations from the HCP dataset, including 31 contrasts spanning language, motor, working memory and social cognition tasks. Task activations are commonly considered more spatially specific markers of functional areas than resting-state networks.

Second, we analysed the HCP multimodal cortical parcellation (HCP-MMP1.0), which integrates information from multiple modalities including task-fMRI, resting-state functional connectivity, cortical myelination (T1w/T2w), cortical thickness, and connectivity gradients (Glasser et al,

2016). This parcellation therefore provides a more comprehensive representation of cortical functional organisation than any single modality.

Across both analyses, improved folding alignment using MSM-HT did not translate into improved functional alignment, supporting the conclusion that cortical folding motifs do not strongly constrain functional organisation in higher-order association cortex.

In the **Discussion Section**, we add:

Page 13, lines 261-263

"The functional measurements used in this study, including tfMRI contrasts and HCP_MMP1.0 parcellations, may not fully capture the complexity of functional organisation. Future work incorporating complementary representations, such as continuous functional gradients, can avoid using discrete network boundaries."

↩ **Back to Summary**

C5. Data processing and alignment pipelines: *The manuscript should provide sufficient detail for replication. Key steps (surface extraction, feature generation, registration parameters) must be fully described.*
For example, it is unclear how surfaces from the different cohorts (HCP adults, dHCP, BIBS neonates) were preprocessed and whether any adjustments (e.g. infant-specific surface refinement) were applied.
The DDR training procedure (e.g. network architecture, training data, number of epochs) is only cursorily mentioned – these details should be in Methods or Supplement.
Clustering parameters (linkage criteria, distance metric) and MSM regularization settings also need specification.
If any code was used, explicit versioning (e.g. FSL/MSM version) should be given.
The authors should verify that these pipelines yield consistent results under slight variations (e.g. random initialization), and report any post-hoc QC (e.g. how many hemispheres failed alignment).

R5. We thank the reviewer for this helpful suggestion regarding reproducibility and methodological clarity. In the revised manuscript we have expanded the Methods section and Supplementary Methods to provide a more complete description of the preprocessing, registration, and clustering pipelines used in this study. The Code and processing pipelines, and images such as folding motifs are available online (see **Comment C6**)

Specifically, we clarified the preprocessing pipelines for both the HCP-YA and neonatal datasets, including surface extraction, feature generation, and coordinate spaces used for analysis. We also added additional details on the DDR training procedure, clustering parameters (distance metric, linkage criterion, and software version), and MSM template generation settings (regularisation parameters and control-point resolutions).

These details has been added to following sections of the revised manuscript and are highlighted in pink:

- **Image Pre-processing (Methods, Page 14)**
- **Optimising pairwise alignment with DDR (Methods, Page 15)**
- **Clustering of cortical folding patterns (Methods, Pages 15-17)**
- **Template generation (Methods, Page 17)**
- **Supplementary Methods on Training and validation of DDR (Supplementary Information S1.2, Page 1)**
- **Supplementary Methods on Justification of clustering approach (Supplementary Information S1.3, Pages 2-3)**

The registration and clustering pipeline used in this study is largely deterministic and does not rely on stochastic initialisation. DDR inference and MSM registration produce identical outputs for repeated runs given the same inputs and parameters, and hierarchical clustering is also deterministic. We additionally performed post-hoc quality control of the registrations and found that all hemispheres completed DDR and MSM alignment successfully. No subjects were excluded due to registration failure; clusters containing fewer than five hemispheres were excluded only to avoid unstable template generation.

↩ **Back to Summary**

C6. Code availability and dataset documentation: *The authors mention use of public datasets (HCP, dHCP, BIBS), and they should state the exact data release versions and subject counts analyzed (with relevant accession IDs or DOIs). The manuscript should include a "Code availability" statement with a stable repository link, and similarly a "Data availability" statement detailing how to access the used imaging data (beyond the general project names).*
Machine learning reporting checklist: Since the study uses deep learning (DDR) and clustering, the authors should complete the Nature machine learning checklist to ensure all relevant items are addressed (e.g. training/validation split, hyperparameter selection, random seed control). The current manuscript gives no information on how DDR was trained (e.g. was there a held-out set?) or whether results are averaged across multiple runs. Any use of learning-based methods also calls for transparency about hardware and computational resources. I recommend the authors explicitly cite that they have satisfied the checklist and consider including a summary table (or citing the completed checklist PDF) to reassure reviewers that methodological rigor is met.

R6. We thank the reviewers for the helpful suggestion regarding code and data availability. In the revised manuscript have now expand this section and separated it into **Data availability** and **Code availability**. The revised text now specifies the exact versions of each data release used and reports the final subject counts included in the analyses. Access instructions for both the public datasets (HCP-YA and dHCP) and the restricted BIBS dataset are now provided. See **revised manuscript Page 19, lines 446-458**.

We also have added stable repository links for analysis code and template resources in the Code availability section. See **Page 19, lines 459-465**.

Specifically, Code and processing pipelines are available at
https://github.com/Yrong-Guo/Hierarchical-surface-registration
Image templates such as folding motifs are displayed and can be downloaded at
https://balsa.wustl.edu/study/ljVNl.
The MSM-HT deformation spheres are available at Zenodo
https://doi.org/10.5281/zenodo.19692916.

Regarding methodological transparency, we have completed the **Nature Machine Learning Checklist**, which is now included as a **supplementary document** and cited in the revised manuscript. See **revised manuscript Page 19, lines 457-458.**

↩ **Back to Summary**

To further bolster the study, I suggest the following feasible analyses or supplements:

C7. Surface area normalization: *As noted above, recompute folding features after normalizing by total hemisphere area or mean curvature. This will test whether motif differences simply scale with brain size. A supplemental figure could show motif frequencies vs. hemisphere area to rule out confounding.*

R7. This comment has been addressed under **Comment C2.2**.

C8. Effect sizes for asymmetry: *Report Cohen's d or asymmetry indices (with 95% CIs) for the key left–right comparisons of motif prevalence. A table of asymmetry effect sizes per motif and a forest plot could be added to supplement.*

R8. This has been addressed in **Comment C3**.

C9. Stability of clustering: *Compute a cluster stability metric (e.g. normalized mutual information) by re-running the clustering on bootstrap resamples of subjects. A supplementary plot of clustering consistency vs. number of clusters would inform on robustness.*

R9. We Thank the reviewer for this practical suggestion regarding the clustering stability and the choice of number of clusters. We performed a bootstrap-based stability analysis. Please check **Comment C2.1** for detailed analysis.

↩ **Back to Summary**

C10. Additional figures/tables: *A full dendrogram (or hierarchical tree) for each lobe's clustering could be provided as a supplementary figure, to show the distance thresholds used. A table listing each motif with descriptive statistics (mean curvature, sulcal depth, area, asymmetry index) would be informative.*

R10. In response to this comment, I have added dendrograms illustrating the hierarchical clustering structure up to the 30-cluster level for both the adult and neonatal datasets (**Figure C6**). These dendrograms are shown alongside the corresponding folding motifs, making it clear which motifs emerge from neighbouring branches of the hierarchy and how the major divisions of the clustering relate to one another (please see the scene files on BALSA (https://balsa.wustl.edu/study/ljVNl). **Table C6** shows the mean curvature and sulcal depth (positive and negative), mean surface area, and asymmetry index. **Table C6** is also included in the **supplementary Fig.S4 (Pages 21-23)**.

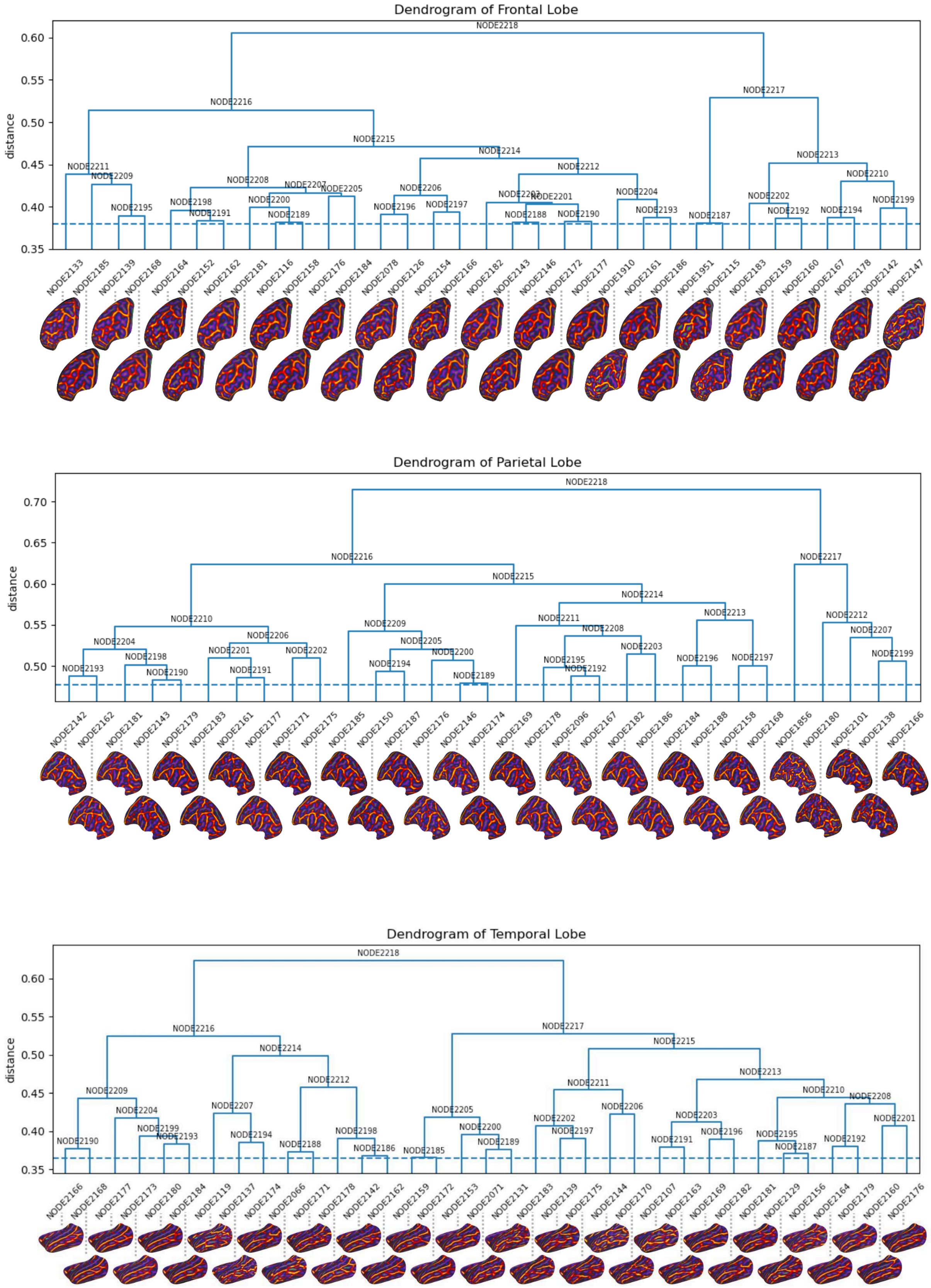
Dendrogram of Frontal Lobe
distance
0.60
0.55
0.50
0.45
0.40
0.35
NODE2218
NODE2216
NODE2217
NODE2215
NODE2214
NODE2213
NODE2212
NODE2211
NODE2209
NODE2210
NODE2208
NODE2207
NODE2205
NODE2206
NODE2204
NODE2203
NODE2201
NODE2202
NODE2198
NODE2200
NODE2195
NODE2196
NODE2197
NODE2199
NODE2191
NODE2189
NODE2188
NODE2190
NODE2193
NODE2187
NODE2192
NODE2194
NODE2133 NODE2185 NODE2139 NODE2168 NODE2164 NODE2152 NODE2162 NODE2181 NODE2116 NODE2158 NODE2176 NODE2184 NODE2078 NODE2126 NODE2154 NODE2166 NODE2182 NODE2143 NODE2146 NODE2172 NODE2177 NODE1910 NODE2161 NODE2186 NODE1951 NODE2115 NODE2183 NODE2159 NODE2160 NODE2167 NODE2178 NODE2142 NODE2147
Dendrogram of Parietal Lobe
distance
0.70
0.65
0.60
0.55
0.50
NODE2218
NODE2216
NODE2217
NODE2215
NODE2214
NODE2210
NODE2211
NODE2213
NODE2212
NODE2209
NODE2208
NODE2207
NODE2204
NODE2206
NODE2205
NODE2203
NODE2201
NODE2202
NODE2200
NODE2193
NODE2198
NODE2190
NODE2191
NODE2194
NODE2189
NODE2195
NODE2192
NODE2196
NODE2197
NODE2199
NODE2142 NODE2162 NODE2181 NODE2143 NODE2179 NODE2183 NODE2161 NODE2177 NODE2171 NODE2175 NODE2185 NODE2150 NODE2187 NODE2176 NODE2146 NODE2174 NODE2169 NODE2178 NODE2096 NODE2167 NODE2182 NODE2186 NODE2184 NODE2188 NODE2158 NODE2168 NODE1856 NODE2180 NODE2101 NODE2138 NODE2166
Dendrogram of Temporal Lobe
distance
0.60
0.55
0.50
0.45
0.40
0.35
NODE2218
NODE2216
NODE2217
NODE2214
NODE2215
NODE2212
NODE2213
NODE2209
NODE2211
NODE2210
NODE2208
NODE2204
NODE2207
NODE2206
NODE2205
NODE2199
NODE2193
NODE2194
NODE2198
NODE2200
NODE2202
NODE2197
NODE2203
NODE2196
NODE2201
NODE2190
NODE2188
NODE2186
NODE2185
NODE2189
NODE2191
NODE2195
NODE2187
NODE2192
NODE2166 NODE2168 NODE2177 NODE2173 NODE2180 NODE2184 NODE2119 NODE2137 NODE2174 NODE2066 NODE2171 NODE2178 NODE2142 NODE2162 NODE2159 NODE2172 NODE2153 NODE2071 NODE2131 NODE2183 NODE2139 NODE2175 NODE2144 NODE2170 NODE2107 NODE2163 NODE2169 NODE2182 NODE2181 NODE2129 NODE2156 NODE2164 NODE2179 NODE2160 NODE2176

Figure C6

Table C6. Descriptive statistics for each motif

Lobe	Motif ID	Cluster size	Mean positive curvature	Mean negative curvature	Mean positive sulcal depth	Mean negative sulcal depth	Mean surface area	Asymmetry index
Frontal	NODE1951	6	0.15	-0.108	0.393	-0.458	37918.656	-0.333
Frontal	NODE2078	17	0.153	-0.108	0.394	-0.477	36902.441	-0.412
Frontal	NODE2116	40	0.152	-0.108	0.388	-0.472	34216.727	0.7
Frontal	NODE2126	8	0.153	-0.109	0.395	-0.469	36193.789	-1
Frontal	NODE2133	7	0.154	-0.108	0.394	-0.462	33781.459	0.714
Frontal	NODE2139	62	0.152	-0.108	0.391	-0.472	33417.712	0.226
Frontal	NODE2142	12	0.153	-0.108	0.386	-0.472	40277.335	-0.333
Frontal	NODE2143	48	0.152	-0.106	0.391	-0.478	35457.976	0.792
Frontal	NODE2146	129	0.152	-0.108	0.392	-0.474	33466.495	0.364
Frontal	NODE2152	21	0.155	-0.109	0.382	-0.471	36577.052	0.048
Frontal	NODE2154	5	0.155	-0.108	0.377	-0.464	39328.895	0.2
Frontal	NODE2158	90	0.153	-0.108	0.39	-0.472	34895.44	0.422
Frontal	NODE2159	8	0.155	-0.109	0.393	-0.465	34639.381	-0.25
Frontal	NODE2160	43	0.153	-0.109	0.39	-0.471	36284.392	-0.581
Frontal	NODE2161	80	0.152	-0.109	0.39	-0.467	33526.301	-0.375
Frontal	NODE2162	58	0.153	-0.108	0.391	-0.469	36037.588	-0.414
Frontal	NODE2164	17	0.153	-0.109	0.391	-0.469	36336.74	0.412
Frontal	NODE2166	95	0.153	-0.108	0.388	-0.471	36537.589	0.284
Frontal	NODE2167	26	0.154	-0.11	0.384	-0.465	37352.981	-0.231
Frontal	NODE2168	36	0.152	-0.108	0.389	-0.464	33232.439	-0.722
Frontal	NODE2172	122	0.153	-0.109	0.392	-0.464	33967.406	-0.557
Frontal	NODE2176	96	0.153	-0.108	0.39	-0.472	35674.152	0.188
Frontal	NODE2177	99	0.153	-0.108	0.391	-0.47	34806.53	-0.232
Frontal	NODE2178	12	0.15	-0.108	0.387	-0.468	39300.119	-0.167
Frontal	NODE2181	294	0.152	-0.108	0.391	-0.474	34310.359	0.184
Frontal	NODE2182	146	0.152	-0.108	0.391	-0.472	34499.602	-0.205
Frontal	NODE2183	134	0.153	-0.109	0.39	-0.473	36141.168	0.269
Frontal	NODE2184	143	0.152	-0.108	0.39	-0.47	35104.672	0.035
Frontal	NODE2185	120	0.153	-0.108	0.391	-0.467	33497.302	-0.15
Frontal	NODE2186	236	0.153	-0.108	0.389	-0.468	33471.926	-0.186

Parietal	NODE2096	27	0.159	-0.109	0.407	-0.509	35502.956	-0.926
Parietal	NODE2101	11	0.16	-0.11	0.403	-0.508	35202.931	0.273
Parietal	NODE2138	5	0.158	-0.111	0.411	-0.52	33812.546	-0.6
Parietal	NODE2142	39	0.159	-0.108	0.409	-0.511	35442.113	0.385
Parietal	NODE2143	36	0.16	-0.109	0.41	-0.505	34654.012	-0.222
Parietal	NODE2146	30	0.159	-0.11	0.407	-0.506	36457.11	-0.867
Parietal	NODE2150	34	0.158	-0.11	0.41	-0.509	36426.193	-0.353
Parietal	NODE2158	47	0.159	-0.109	0.408	-0.514	36139.296	-0.064
Parietal	NODE2161	27	0.16	-0.108	0.406	-0.508	35452.486	-0.259
Parietal	NODE2162	104	0.159	-0.109	0.408	-0.507	34917.016	0.519
Parietal	NODE2166	20	0.161	-0.11	0.411	-0.514	33273.503	-0.3
Parietal	NODE2167	94	0.159	-0.109	0.409	-0.509	36139.636	-0.596
Parietal	NODE2168	156	0.158	-0.109	0.407	-0.506	34523.326	0.5
Parietal	NODE2169	55	0.159	-0.109	0.408	-0.504	36863.658	0.091
Parietal	NODE2171	28	0.16	-0.109	0.405	-0.505	35663.34	0.571
Parietal	NODE2174	57	0.158	-0.108	0.412	-0.514	35477.042	-0.684
Parietal	NODE2175	75	0.16	-0.11	0.408	-0.506	34185.237	0.493
Parietal	NODE2176	24	0.158	-0.108	0.412	-0.519	38151.767	-0.667
Parietal	NODE2177	87	0.16	-0.109	0.409	-0.508	34908.263	0.034
Parietal	NODE2178	110	0.158	-0.108	0.409	-0.51	36291.744	-0.4
Parietal	NODE2179	317	0.159	-0.109	0.408	-0.509	34818.465	0.451
Parietal	NODE2180	5	0.158	-0.112	0.405	-0.506	35650.664	-0.2
Parietal	NODE2181	141	0.159	-0.109	0.406	-0.507	35196.182	0.39
Parietal	NODE2182	244	0.159	-0.109	0.409	-0.511	34352.975	-0.418
Parietal	NODE2183	60	0.159	-0.11	0.409	-0.502	34921.427	0.467
Parietal	NODE2184	84	0.158	-0.109	0.41	-0.506	36194.021	-0.357
Parietal	NODE2185	54	0.159	-0.109	0.413	-0.515	36213.634	-0.667
Parietal	NODE2186	103	0.158	-0.109	0.412	-0.511	36513.442	-0.689
Parietal	NODE2187	75	0.158	-0.109	0.41	-0.512	36909.589	-0.093
Parietal	NODE2188	69	0.159	-0.109	0.405	-0.503	35557.953	0.797
Temporal	NODE2071	17	0.147	-0.098	0.385	-0.541	20841.019	1
Temporal	NODE2129	10	0.146	-0.099	0.366	-0.519	21081.184	1
Temporal	NODE2131	7	0.145	-0.101	0.39	-0.54	21197.046	0.143

Temporal	NODE2139	22	0.149	-0.1	0.382	-0.535	20905.679	1
Temporal	NODE2142	40	0.145	-0.098	0.394	-0.525	20706.321	-0.65
Temporal	NODE2153	35	0.149	-0.1	0.392	-0.547	19265.919	0.886
Temporal	NODE2156	12	0.147	-0.1	0.387	-0.535	19778.717	0.833
Temporal	NODE2159	44	0.146	-0.098	0.396	-0.54	20489.587	0.409
Temporal	NODE2160	31	0.148	-0.099	0.387	-0.528	20269.169	0.355
Temporal	NODE2162	123	0.146	-0.098	0.391	-0.525	20807.521	-0.593
Temporal	NODE2163	16	0.148	-0.1	0.381	-0.535	20461.406	1
Temporal	NODE2164	161	0.147	-0.099	0.391	-0.542	19570.467	0.789
Temporal	NODE2166	85	0.146	-0.097	0.392	-0.521	20013.846	-0.812
Temporal	NODE2168	211	0.145	-0.098	0.393	-0.525	20081.262	-0.526
Temporal	NODE2169	31	0.146	-0.099	0.39	-0.53	20575.338	0.548
Temporal	NODE2170	8	0.151	-0.099	0.38	-0.538	20956.388	1
Temporal	NODE2171	11	0.146	-0.098	0.387	-0.519	21761.02	-0.273
Temporal	NODE2172	156	0.147	-0.099	0.39	-0.535	19589.283	0.346
Temporal	NODE2173	150	0.146	-0.098	0.393	-0.533	19754.839	0.053
Temporal	NODE2174	21	0.147	-0.099	0.379	-0.526	21338.497	0.81
Temporal	NODE2175	15	0.151	-0.101	0.376	-0.531	20404.051	1
Temporal	NODE2176	58	0.146	-0.099	0.388	-0.534	21233.078	0.586
Temporal	NODE2177	130	0.146	-0.098	0.394	-0.531	19766.891	-0.308
Temporal	NODE2178	31	0.146	-0.098	0.388	-0.513	21981.212	-0.935
Temporal	NODE2179	42	0.15	-0.1	0.387	-0.543	19500.004	1
Temporal	NODE2180	424	0.146	-0.098	0.394	-0.532	19529.27	-0.481
Temporal	NODE2181	68	0.148	-0.099	0.39	-0.54	20363.395	0.765
Temporal	NODE2182	41	0.147	-0.1	0.396	-0.546	21558.057	1
Temporal	NODE2183	24	0.146	-0.1	0.378	-0.531	21533.46	1
Temporal	NODE2184	178	0.147	-0.098	0.395	-0.529	19641.251	-0.191

↩ Back to Summary

C11. Sex and age effects: *If sample sizes permit, test whether motifs differ by sex or age (within HCP adult range). A quick ANOVA per motif (or regression on age) could reveal whether the reported effects hold after controlling for these factors.*

R11. We tested for potential demographic confounds by examining age and sex effects on HCP motif membership. No significant age differences were observed across clusters within the HCP-YA cohort. A global χ^2 test revealed a statistically significant association between sex and cluster membership; however, effect sizes were small to moderate (Cramér's V = 0.18–0.23), indicating that sex explains only a minor proportion of variance and does not drive the clustering structure.

↩ **Back to Summary**

C12. Genetic model: *If possible, run a simple ACE twin model (using e.g. OpenMx or similar) to estimate h² for one or two representative motifs. Even a back-of-envelope figure could confirm that the MZ–DZ difference aligns with high genetic influence, as in prior studies*

R12. We thank the reviewer for this comment and the ACE twin models would largely strengthen the argument for the genetic influence of cortical folding motifs. To gain adequate statistical power, instead of using the binary variable – twins presence/absence in one cluster – we used the continuous correlation score to measure the similarity of each subject's two hemispheres to the folding motif. Folding motifs with more than 50 hemispheres are included in the analysis. Bivariate ACE models were initially fitted and compared against nested bivariate AE models using likelihood-ratio tests and AIC; AE was retained when shared environmental effects did not improve model fit. Given the absence of evidence for shared environment effects, we report AE estimates of heritability (h^2) separately for left and right hemispheres. We additionally report the genetic correlation between hemispheres (R_g^{LR}) from the bivariate model.

Across lobes, heritability estimates for left and right hemisphere similarity-to-template measures were moderate in magnitude (Mean ± SE: frontal lobe left: 0.478±0.049, right: 0.326±0.034; parietal lobe left: 0.348±0.094, right: 0.419±0.05; temporal lobe left: 0.495±0.034, right: 0.44±0.043), with only small hemispheric differences. Although heritability estimates were computed separately for left and right hemispheres, these do not imply independent genetic influences, as genetic correlations between hemispheres were high. The genetic correlation is highest in frontal clusters (R_g^{LR} close to 1), intermediate in parietal lobe (R_g^{LR}=0.778–0.999), and lowest in temporal lobe (R_g^{LR}=0.434–0.719), indicating the genetic influence is almost completely shared in frontal cortex, and least shared in temporal cortex. These results demonstrate that cortical folding motifs are moderately heritable, and have shared genetic influences, rather than independent hemispheric effects.

We now add this analysis in the **revised manuscript (results Page 5, Lines 109-119; methods Page 18, Lines 410-419)**, and moved the original analysis to the **Supplementary Results (S2.2; Table S1).**

Table C7. AE model estimates of heritability (h^2) for left and right hemisphere similarity-to-template measures, and the genetic correlation between hemispheres (rgLR), across representative cortical folding clusters.

Lobe	Motif	h_L^2	h_R^2	R_g^{LR}
Frontal	NODE2139	0.477	0.284	1
Frontal	NODE2146	0.533	0.344	1
Frontal	NODE2158	0.487	0.358	1
Frontal	NODE2161	0.468	0.305	1
Frontal	NODE2162	0.342	0.257	1
Frontal	NODE2166	0.447	0.288	1
Frontal	NODE2172	0.487	0.338	1
Frontal	NODE2176	0.508	0.383	1
Frontal	NODE2177	0.472	0.33	1

Lobe	Motif	h_L^2	h_R^2	R_g^{LR}
Frontal	NODE2181	0.496	0.316	1
Frontal	NODE2182	0.534	0.354	1
Frontal	NODE2183	0.473	0.331	1
Frontal	NODE2184	0.441	0.296	1
Frontal	NODE2185	0.532	0.353	1
Frontal	NODE2186	0.529	0.348	1
Parietal	NODE2162	0.323	0.48	0.895
Parietal	NODE2167	0.428	0.365	0.91
Parietal	NODE2168	0.315	0.452	0.902
Parietal	NODE2169	0.207	0.375	1
Parietal	NODE2174	0.504	0.444	0.778
Parietal	NODE2175	0.486	0.497	0.835
Parietal	NODE2177	0.341	0.452	0.961
Parietal	NODE2178	0.286	0.385	0.875
Parietal	NODE2179	0.393	0.463	0.835
Parietal	NODE2181	0.312	0.438	0.828
Parietal	NODE2182	0.48	0.483	0.85
Parietal	NODE2183	0.281	0.396	0.953
Parietal	NODE2184	0.223	0.364	0.908
Parietal	NODE2185	0.461	0.35	0.999
Parietal	NODE2186	0.283	0.341	0.969
Parietal	NODE2187	0.336	0.437	0.824
Parietal	NODE2188	0.261	0.404	0.933
Temporal	NODE2162	0.465	0.402	0.61
Temporal	NODE2164	0.539	0.494	0.549
Temporal	NODE2166	0.454	0.398	0.719
Temporal	NODE2168	0.492	0.43	0.669
Temporal	NODE2172	0.53	0.457	0.574
Temporal	NODE2173	0.534	0.392	0.647
Temporal	NODE2176	0.448	0.426	0.479
Temporal	NODE2177	0.467	0.489	0.619

Lobe	Motif	h^2_L	h^2_R	R^{LR}_g
Temporal	NODE2180	0.53	0.461	0.645
Temporal	NODE2181	0.485	0.522	0.434
Temporal	NODE2184	0.502	0.428	0.606

↩ **Back to Summary**

C13. Several limitations and caveats should be acknowledged:

R13. We thank the reviewer for these helpful suggestions. We have revised the manuscript to clarify these limitations and added the relevant discussion in the sections indicated below.

• Sample and population: The HCP sample (young adults) is relatively homogeneous (ethnicity, age), and twins come from one cohort. Findings may not generalize to other populations or age groups. Similarly, the neonate cohorts (dHCP, BIBS) have narrow age windows and may not represent full developmental variability. The paper should note these limits.

We have now acknowledged the homogeneity of the datasets as a limitation in the Discussion:
Discussion Section (Page 13, Lines 263-265)
"Finally, both cohort characteristics and analytical choices may influence the generalisability of these findings. The HCP-YA is relatively homogeneous in ethnicity and age, while neonatal cohorts (dHCP, BIBS) have narrow age ranges."

Discussion Section (Page 13, Lines 266-270)
"In this regard, dHCP is now being followed longitudinally into childhood, providing an important opportunity to directly examine the developmental evolution of cortical folding motifs. Future work should expand MSM-HT to more diverse and longitudinal datasets, and integrate multimodal data, including functional connectivity and genetic information, to better characterise developmental trajectories and improve the identification of robust biomarkers."

• Cross-sectional design: The study is not longitudinal, so "from birth to adulthood" is inferred by comparing different cohorts. Cohort effects or differences in data quality between neonates and adults could affect motif comparisons. This should be mentioned when discussing developmental consistency.

We have now clarified this point when discussing developmental consistency:
Discussion Section (Page 13, Lines 256-259)
"Developmental comparisons between neonates and adults also require careful interpretation, as these analyses are cross-sectional. Observed differences in motif incidence may reflect both biological maturation and methodological factors, including the lower spatial resolution relative to the brain scale, which reduces the detectability of finer sulcal branches and interruptions."

• Method complexity: The hierarchical registration and clustering pipeline is complex; small implementation choices might impact results. For instance, the order of lobe alignment (e.g. frontal first vs. temporal first) could hypothetically bias motif discovery. The authors should make clear what decisions were made and note them as potential sources of bias.

We have now acknowledged this methodological limitation in the Discussion:
Discussion Section (Page 13, Lines 263-266)
"Finally, both cohort characteristics and analytical choices may influence the generalisability of these findings. The HCP-YA is relatively homogeneous in ethnicity and age, while neonatal cohorts (dHCP, BIBS) have narrow age ranges. In addition, the hierarchical registration and clustering pipeline involves design choices, such as the order of lobe alignment, that may affect the resulting motifs."

• Over-interpretation of function coupling: The phrase "folding-functional links" in the title suggests a causal or tight relationship, but the analysis is correlational. Given known genetic pleiotropy and many-to-many mapping between structure and function, the authors should avoid implying that folding motifs dictate functional networks.

Instead, they might say motifs are associated with certain functional patterns, with residual variability in association cortex.

We have revised the relevant text in the Discussion to clarify that the relationship between folding patterns and functional organisation is associative rather than causal.
Discussion Section (Page 12, Lines 226-232)
"In these areas, folding is much more irregular, and their correspondence with functional organisation is much weaker. Supplementary analyses further quantified this relationship, showing that the correlation between folding similarity and functional similarity across individuals is small ($\rho = 0.068$–0.104 across lobes), indicating that folding patterns explain only a limited proportion of inter-individual variability in functional organisation. One possible interpretation for these results is that cortical folding aligns more with primary functional regions because early-forming folds are under stronger genetic control, whereas later-forming folds in association cortex are influenced by more variable biomechanical and environmental factors."

Discussion Section (Page 13, Lines 249-251)
"Together, these findings suggest that cortical folding motifs reflect developmental constraints that interact with genetic, biomechanical, and environmental factors, rather than deterministically defining functional organisation."

- ***Heritability nuances: As noted, "heritability" can be misconstrued. Unless a full genetic model is presented, it would be safer to say motifs show evidence of genetic influence (greater similarity in MZ twins) rather than report a numeric heritability. Also, environmental and measurement factors (e.g. image noise) will reduce apparent heritability.***

As described in our response to **Comment C12**, we have now added twin modelling using ACE/AE models to estimate heritability.
Result Section – Cortical folding motifs are heritable (Page 4, Lines 121-127).

We further clarified the interpretation of these results in the Discussion:
Discussion Section (Pages 12-13, Lines 234-236):
"In line with previous heritability research in HCP (Schmitt et al., 2021), our study found a stable genetic influence within lobes in the adult brain, indicated by MZ twins being significantly more likely to fall into the same folding clusters than the DZ twins. The ACE/AE models also exhibit moderate heritability across lobes. Indeed, cortical areas are influenced by genetic patterning that contributes to the early organisation of the functional regions, where early cortical folds are formed related to them (Rakic, 1988; Régis et al., 2005; Budday et al., 2015)**"**

By tempering conclusions with these points and clearly explaining methodological choices, the authors can present a balanced, transparent study.
Minor points: The manuscript contains a few minor issues (typos, unclear figure labels, etc.) that should be corrected in revision. For brevity these are not enumerated here but can be provided separately if needed.

Conclusion: *Guo et al. tackle an important problem of quantifying cortical folding variability and its genetic/functional correlates. The approach is creative, but the analysis requires stronger validation and clearer reporting to meet field standards of rigor and reproducibility. Addressing the points above – especially clarifying the novelty of MSM-HT, providing statistical detail for asymmetry and heritability claims, and ensuring all methods (and code) are fully documented – will greatly improve the manuscript. The suggested additional analyses and caution on interpretation will also enhance the credibility of the conclusions.*

↩ **Back to Summary**

Reviewer #2 (Remarks to the Author):

In the current work, Guo et al developed and applied a novel technique to investigate cortical folding in the human cortex, focussing on a young adult and a perinatal sample. In short, they observed different types of folding, with slight patterns of heritability and also asymmetry in temporal regions. Finally they explored whether individuals with similar folding patterns would have similar functional activations.

Overall this is very nice work and a relevant domain for exploration in terms of neurobiology and cognitive implications. I have some questions that may help the paper.

C14. Interpretation of difference between neonates and adults is a bit speculative due to potential methodological causes of differences? To what extent are there also alterations or refinement in folding in the first 20 years of life?

R14. We thank the reviewer for this important point. We agree that differences in motif incidence between neonates and adults should be interpreted cautiously, as they may reflect both biological maturation and methodological factors.

While our cross-sectional design does not allow us to directly track longitudinal refinement of the reference motifs, by comparing our results to prior literature it is possible to build up a consistent developmental pattern. We know that major cortical folds are largely established prenatally and that their appearance at birth shows broad consistency with patterns observed in adults. By contrast, higher order cortical folding – particularly in association cortex – undergoes continuous refinement during the first year of life (Hill et al., 2010; Li et al., 2014). This refinement also influences curvature, surface area, and branching complexity, which would align with the observation of greater diversity of folding motifs in adults relative to neonates (Hill et al., 2021).

Methodological factors may also contribute to observed differences. To minimise the differences of the processing pipeline between adults and neonates, we used the deep learning-based surface pipeline for dHCP and BIBS data, which aside from tuning cortical segmentation to the specific contrast properties of neonatal (T2) images, applies the same surface inflation, spherical projection and feature extraction steps as the adult FreeSurfer pipeline (please see **Reviewer 1 Comment C5 and revised manuscript Page 14, lines 311-317**). However, since neonatal MRI has lower spatial resolution relative to brain scale, it is possible that the resulting partial volume effects (blurring of tissue boundaries) would impact detection of finer sulcal branches and interruptions. We have further acknowledged this in the revised manuscript Discussion **Page 13, Lines 257-259**.

Page 14, lines 311-317
"Neonatal surfaces were extracted using a recently published bespoke deep learning-based pipeline (Ma et al., 2024), trained on the outputs from the original dHCP pipeline (Makropoulos et al., 2018). This pipeline adapts the FreeSurfer pipeline to the very different MRI properties of neonatal scans, which have inverted tissue contrast and relatively low resolution. The surface inflation and feature extraction in dHCP deep learning is a direct reimplementation of Freesurfer (Fischl et al., 2012). Visual quality control (QC) of the surface output from the learning-based pipeline was improved in terms of anatomical and topological correctness (Ma et al., 2024). We use neonatal spheres, sulcal depth maps, mean curvature, and cortical labels from the DRAW-EM regional atlas (Gousias et al., 2010; Makropoulos et al., 2014; Makropoulos et al., 2016), which differs from the Desikan-Killiany atlas, but ensures accurate segmentation of the neonatal brain structures that is appropriate to the different contrast of neonatal MRI and shape of neonatal cortex. For more details about the deep learning-based neonatal pipeline, see Supplementary Methods S1.1."

Page 13, Lines 257-259.
"Observed differences in motif incidence may reflect both biological maturation and methodological factors, including the lower spatial resolution relative to the brain scale, which reduces the detectability of finer sulcal branches and interruptions."

Despite the relatively large sample sizes used in our study, it remains possible that some rare folding motifs are not fully captured in either cohort. Longitudinal datasets will therefore be essential to disentangle the development refinement from methodological effects. In this regard, dHCP will now be followed up into through childhood, providing an important opportunity to directly examine the developmental evolution of cortical folding motifs.

Page 13, Lines 264-270
"The HCP-YA is relatively homogeneous in ethnicity and age, while neonatal cohorts (dHCP, BIBS) have narrow age ranges. In addition, the hierarchical registration and clustering pipeline involves design choices, such as the order of lobe alignment, that may affect the resulting motifs. In this regard, dHCP is now being followed longitudinally into childhood, providing an important opportunity to directly examine the developmental evolution of cortical folding motifs. Future work should expand MSM-HT to more diverse and longitudinal datasets, and integrate multimodal data, including functional connectivity and genetic information, to better characterise developmental trajectories and improve the identification of robust biomarkers."

↩ **Back to Summary**

C15. For the functional analysis,
This comment was unfinished.

C16. Why were insula and temporal lobe combined in one lobe in the analysis?

R16. We thank the reviewer for raising this point. Inspection of the sulcal depth maps showed that the insular cortex exhibits relatively limited inter-individual variability compared with other cortical regions. To quantify this, we calculated the average pairwise cross-correlation similarity between sulcal depth maps of individual insular regions in MSM-P space, which was high (mean cross-correlation = 0.964). Because our clustering approach aims to identify folding motifs based on variability in sulcal patterns, analysing the insula as a separate region did not produce stable clusters. For this reason, the insula was included within the temporal lobe analysis, allowing it to be incorporated into nearby folding patterns without introducing an additional region with very limited variability.

To clarify this choice, we have added the following sentence to the Methods section:

Page 15, Lines 331-332
"Once sulcal depth maps are coarsely aligned, the overlap of cortical folding patterns for each pair of hemispheres may be separately assessed for each of the frontal, parieto-occipital, and temporal lobes – using a combination of Dice overlap and cross-correlation (CC). The definition of these lobar regions is shown in Supplementary Fig.S13."

Then in **Supplementary Information Page 18**:

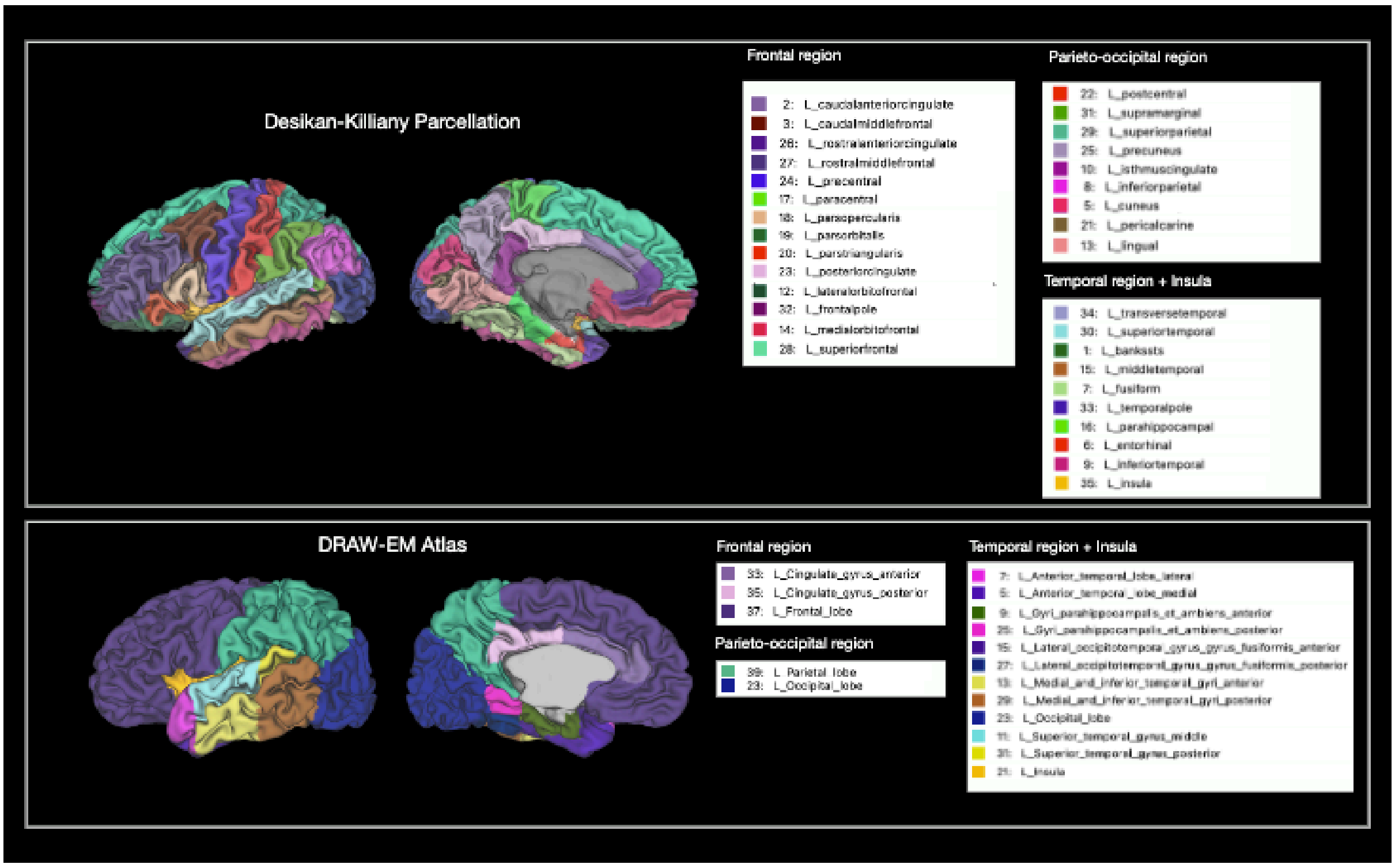

Fig. S13. Lobar regions for hierarchical clustering. Parcels included in the frontal, parieto-occipital, and temporal lobe regions are shown for adult (Desikan–Killiany) and neonatal (DRAW-EM) datasets, illustrating differences in atlas definitions across age groups. The insular cortex was grouped with the temporal lobe in the clustering analysis because sulcal depth patterns in the insula exhibited very limited inter-individual variability (mean pairwise cross-correlation = 0.964 in MSM-P space), which did not yield stable clusters when analysed separately

↩ Back to Summary

C17. For the functional mapping, so the idea would be that people with similar folding features would also have similar functional activations?

R17. Yes, this was the underlying hypothesis motivating the development of the methodological framework described in this paper but the results we found contradicted this assumption. We tested whether individuals belonging to the same folding motif cluster showed greater similarity in functional organisation compared to individuals from different clusters. This was evaluated using both task-fMRI activation maps and the multimodal HCP_MMP1.0 cortical parcellation. However, our results did not support this hypothesis: functional similarity was not consistently higher within folding clusters than between clusters (please also see further experiments conducted in response to **Reviewer 1** **R4.1**). These findings suggest that similar cortical folding configurations do not necessarily imply similar functional activation patterns, particularly in association cortices where functional organisation may be influenced by developmental and environmental factors.

↩ **Back to Summary**

C18. Did authors also test for covariance, e.g. if there is a relation between being similar folding organization in temporal lobe for individual x and y with organization being similar in the frontal lobe? Or are the folding patterns independent per lobe?

R18. We thank the reviewer for this insightful suggestions. In the original Supplementary Results, we did investigate this through assessing the co-occurrence of cortical folding patterns across lobes. We observed significant co-occurrences between 13 frontal-parietal, 46 parietal-temporal, and 1 frontal-temporal co-occurrences (p-corrected < 0.005). However, most of these showed small effect sizes ($\kappa < 0.2$), suggesting weak overall agreement. 14 pairs of co-occurrence achieved moderate to substantial agreement. However, these high-agreement pairs are primarily associated with larger cluster sizes and more common folding patterns, suggesting that folding co-occurrence is more likely in widely shared anatomical variants. This also indicated that the analysis may be underpowered for less frequent motifs, where a limited sample size within individual clusters could reduce sensitivity to detect meaningful co-occurrence.

We have since realised that our initial co-occurrence analysis suffered from the confound that certain clusters would contain many more subjects than others. Therefore, in the revised manuscript, we performed an additional hemisphere-level analysis that does not rely on motif labels. This analysis used only the sulcal depth and its pairwise similarities, and was therefore independent of the clustering structure. For each lobe, we computed a 2220 × 2220 hemisphere–hemisphere similarity matrix (the same matrices used for hierarchical clustering), which captured how similar each hemisphere's folding pattern was to every other hemisphere in the cohort. We then assessed cross-lobar coupling by correlating the upper-triangular elements of two such matrices (e.g. frontal and parieto-occipital). This correlation quantified whether the pattern of inter-individual variation in one lobe predicted the analogous pattern in another lobe.

To test whether the observed correlation could arise by chance, we used a permutation-based Mantel test ($N = 5000$). In each permutation, the hemisphere indices of one lobe's similarity matrix were randomly relabelled, such that the similarity between any two specific hemispheres remained unchanged, while correspondence between hemisphere identities across two lobes was destroyed. This generated a null distribution representing cross-lobar correlations expected in the absence of any shared mechanism. The observed correlation lay far outside this distribution (frontal vs. parieto-occipital: $r = 0.36$, $p < 0.0002$; frontal vs. temporal: $r = 0.23$, $p < 0.0002$; parieto-occipital vs. temporal: $r = 0.37$, $p < 0.0002$), demonstrating the cross-lobar relationships that are independent from motif cluster size. These results suggest weak but statistically reliable broad cross-lobar dependencies in folding patterns at the population level. Therefore we add them into the revised **Supplementary Result (Pages 4-5, Lines 106-151)**, highlighted in pink.

↩ **Back to Summary**

Revision for “Motifs of brain cortical folding from birth to adulthood: structural asymmetry and folding-functional links”

We thank both reviewers for their constructive evaluation and for supporting acceptance of the manuscript. We are grateful for the suggestions that improved the abstract's alignment with the Results and the long-term reproducibility of the released code. Below we respond to each remaining point in turn.

Reviewer Comments

Reviewer #1 (Remarks to the Author):

I am satisfied that the authors have adequately addressed the substantive reviewer concerns. In particular, they have clarified the methodological novelty of MSM-HT, added quantitative validation against single-template registration, performed clustering stability and robustness analyses, provided additional asymmetry effect-size reporting, expanded the functional coupling analyses, and improved the methodological, code, data availability, and limitations sections.

I do not have remaining substantive concerns and support acceptance of the manuscript. I would only recommend one minor editorial correction before final publication: the abstract should be aligned with the Results regarding neonatal temporal-lobe asymmetry, which is described as a trend that did not reach statistical significance. This wording can be softened without altering the overall conclusions.

We thank the reviewer for this detailed recommendation; we have changed the text (**Page 1, Lines 8-9**) in the abstract from

“Crucially, we highlight significant hemispheric asymmetry within the temporal lobe for both adults and neonates.” to

“Crucially, we highlight significant hemispheric asymmetry within the temporal lobe in adults, with a consistent trend in neonates.”

Reviewer #1 (Remarks on code availability):

The authors have provided a public GitHub repository for the MSM-HT framework, including the core surface-registration code, trained DDR registration models, and analysis scripts used for the manuscript.

The README provides a useful overview of the workflow and dependencies, and the manuscript/reporting materials appropriately point readers to the repository.

The code is best described as research/HPC pipeline code rather than a fully packaged, turnkey software release. Some scripts appear to retain hard-coded paths, local compute assumptions, and fixed parameters that may require adaptation by external users. These issues do not, in my view, preclude acceptance, because the central methodological code and trained models are publicly available. However, before publication, I would encourage the authors to improve long-term reproducibility by archiving a fixed release, adding an environment file or container specification, and providing a minimal example command or test dataset illustrating expected inputs and outputs.

Overall, the code availability is adequate for publication, with minor recommended improvements for portability and reproducibility.

We thank the reviewer for these constructive suggestions on reproducibility. In response, we have (i) archived a fixed, versioned release of the MSM-HT framework [GitHub release "Release accompanying the MSM-HT manuscript", https://github.com/Yrong-Guo/Hierarchical-surface-registration; Zenodo DOI: 10.5281/zenodo.20507642], and (ii) added an environment specification (py39_environment.yml) capturing the software dependencies.

Regarding a minimal runnable example: because the pipeline involves multiple stages each requiring quality control of the bash code and interactions with HPC clusters to produce meaningful output, a reduced end-to-end example risks being misleading rather than illustrative. Instead, we have ensured the README documents the workflow, dependencies, and expected inputs and outputs at each stage, so that external users can adapt the provided scripts and trained models to their own data. We believe this provides a practical and honest basis for reuse of the framework.

Reviewer #2 (Remarks to the Author):

I thank the authors for their detailed replies, and have no further comments.

Reviewer #2 (Remarks on code availability):

I have visually inspected the code and it looks usable.

We thank the reviewer for their constructive suggestions throughout the review process.
