# Supplementary material for: Motifs of brain cortical folding from birth to adulthood
Source: Nat Commun. 2026 Sep 3;17:10482. doi: 10.1038/s41467-026-77314-5 (PMC13635187; doi:10.1038/s41467-026-77314-5)
Supplement: Supplementary file 3 [file 41467_2026_77314_MOESM1_ESM.pdf]

S1 Supplementary Methods

S1.1 Deep learning-based neonatal pipeline All dHCP and BIBS data are processed using the dHCP deep learning-based neonatal pipeline, detailed in Ma et al. (1). In brief, T2w images were first bias-corrected with the N4 algorithm (2), then rigidly and affinely aligned to the 40-week neonatal volumetric template (3). A 3D CNN with a U-Net architecture was then used to predict a stationary velocity field that deforms the dHCP 40-week population-average surface template to the boundaries of the white and pial cortical boundaries of each subject's T2w MRI. Anatomical surfaces were then inflated using a GPU-parallelised implementation of Freesurfer's `mris_inflate` (4, 5). Finally, an unsupervised learning-based spherical mapping approach was used to generate spheres for each individual, by deforming the 40-week template sphere (generated from FreeSurfer's `mris_sphere`) to minimise metric distortions between the learnt sphere and each individual's white matter surface (in this way empirically matching the results that would be obtained from using Freesurfer). The resulting pipeline produces white, pial, inflated, and spherical surfaces for each subject, with corresponding metric files summarising cortical thickness, curvature and sulcal depth. Because the deep learning-based pipeline does not perform regional segmentation, anatomical lobes were obtained from the DRAW-EM atlas (6) integrated into the original dHCP pipeline (7). These labels were defined in volume space and subsequently projected onto the surface using ConnectomeDB `volume-label-to-surface-mapping` procedure.

S1.2 Training and validation of DDR The DDR algorithm was obtained from https://github.com/mohamedasuliman/DDR. Training was performed using the Adam optimiser with a learning rate of 10^{-4}. For the HCP-YA dataset, DDR was trained to align all pairs of hemispheres using five-fold cross-validation, which was used to assess model performance on unseen data. In each fold, 1,970,472 pairs were used for training and 492,618 pairs for testing. The network was trained for 51-69 epochs with a batch size of 1. Alignment accuracy in the test cohort was evaluated using mean square error (MSE) and cross-correlation (CC), with a five-fold average CC of 0.750. The trained model is available from https://github.com/Yrong-Guo/Hierarchical-surface-registration.

For neonatal data, DDR was trained using all hemispheres from the dHCP cohort (1,027,461 hemispheric training pairs) and generalisation was evaluated on an independent cohort of BIBS subjects (19,900 pairs). The image acquisition and cortical processing were identical for both cohorts. The network was trained for 15 epochs with a batch size of 1. Following alignment, the mean CC of sulcal depth maps was 0.717 ± 0.066 for dHCP and 0.720 ± 0.054 for BIBS, suggesting DDR performs consistently across training and testing datasets (Fig.S5; Cohen's $d = -0.044$; 95% CI $[0.058, 0.030]$; pairs are non-independent, so the CI reflects pair count rather than independent replicates). After validation, the trained DDR model was applied to all neonatal hemispheres (dHCP and BIBS) to compute pairwise alignments used for the clustering analysis.

S1.3 Evaluation of MSM-HT registration To validate the MSM-HT pipeline, we conducted several validation steps to benchmark its performance against conventional methods.

Validating registration performance We generate metric maps that convey the mean and standard deviation (STD) of the curvature for each vertex across all individuals within each cluster, aligned by MSM-HT and MSM-P registration. Paired examples were compared for the STD values with a Wilcoxon signed-rank test. The result shows that MSM-HT is visually sharper (see main paper Fig.5) and significantly reduces the variance across subjects within the clusters ($p < 0.001$). Furthermore, after combining the registration across different lobes, the STD values are still significantly lower within each cluster than MSM-P ($p < 0.001$).

To quantify how MSM-HT performs relative to alignment to a single population template (MSM-P), we compute, for each cluster, vertex-wise STD maps of sulcal depth across subjects after registration. To focus on regions that are most challenging to align, a reference variability map was constructed by taking the per-vertex maximum variability observed across MSM-HT and MSM-P, thereby capturing vertices that exhibit high residual variability under either registration. Vertices were then ranked

according to this reference variability, and subsets corresponding to the top 70%, 60%, 50%, 40%, 30%, 20%, 10%, and 1% most variable vertices were analysed. Within each subset, the mean residual variability was calculated separately for MSM-HT and MSM-P, producing threshold–response curves describing how alignment precision changes when progressively focusing on highly variable cortical regions (Fig.S6). Across all clusters, MSM-HT consistently exhibits lower residual variability than MSM-P, with the largest improvements observed in the most variable regions. This quantitative result supports the qualitative observations in the main paper Fig.5, and indicates that hierarchical cluster-specific registration improves the alignment of heterogeneous folding patterns compared with conventional single-template approaches.

Areal distortion for each vertex is defined as the average of the distortion for all triangles adjacent to the vertex p, $\frac{1}{N_p}\sum_i \log_2\left(\frac{\mathrm{AD}_i}{\mathrm{AO}_i}\right)$, where AO_i and AD_i are the area of triangle i before and after distortion, and N_p is the number of triangle adjacent to vertex p. To ensure a fair comparison, we controlled for area distortion – adjusting regularisation terms (i.e. lambda, see main paper) until the 95th percentile of area distortions matched between MSM-HT and MSM-P. This constraint was applied consistently across all analyses comparing MSM-HT and MSM-P (The distribution of area distortion is shown in Fig.S7).

Justification of clustering approach Many different clustering approaches exist, including spectral clustering (SP) and hierarchical affinity propagation (Hierarchical AP) (8, 9). To validate our choice of hierarchical clustering, we compared MSM-HT against these methods in terms of: a) the sharpness of the generated templates – validated from within template curvature STD); b) the diversity of the generated templates – assessed from the CC of different templates derived from the same method. This is compared against a baseline generated by randomly allocating individual hemispheres to 500 clusters (with cluster sizes scaled to match the distribution of the MSM-HT templates). The implementations of both SP and AP are built-in functions of `sklearn.cluster`, and hierarchical AP follows the detailed description in Duan et al. (9). In order to save on computation costs, this is only performed in the frontal lobe.

Fig.S8 shows that the STD map was found to be significantly lower in MSM-HT clusters, followed by SP, hierarchical AP, and random clusters, indicating less registration uncertainty within MSM-HT clusters ($p < 0.05$). The between-template similarity shown by CC for MSM-HT clusters is higher than for SP, hierarchical AP and random clusters, indicating more compact and well-defined clusters. Fig.S9 shows that MSM-HT generates visually sharper mean maps with more detail and greater variability than templates generated from SP, hierarchical AP, or random allocation. For example, single-segment precentral sulcus clusters such as cluster F2126 are only uncovered by MSM-HT, but only two or three segments were found in SP and hierarchical AP clusters. In MSM-HT, most of the clusters show more detailed tertiary folds branched from the major folds, but SP clusters are more blurry around the major folds, and worse in hierarchical AP clusters. An example of this is clusters F1951 and F2142 in MSM-HT compared with cluster SP18 in SP clusters and cluster HAP5 in hierarchical AP clusters. Random clusters, which serve as a baseline for comparison, exhibited the least defined patterns among all methods evaluated. These clusters generally lack details of segments or branching patterns and often resemble the overall population average.

In agglomerative hierarchical clustering, the distance between two clusters is determined from the maximum distance between individual data points contained within those clusters (known as complete linkage) to generate more compact clusters. In the proposed framework, we cut the dendrogram at the level of thirty cortical folding clusters, where clusters containing fewer than five hemispheres were excluded to avoid the generation of unstable templates (Figs.S10 and S11; 3 clusters in the frontal lobe, 1 in the parieto-occipital lobe, and 5 in the temporal lobe).

To assess the robustness of motif identification and the choice of cluster number, we performed a bootstrap-based stability analysis. For each lobe, hierarchical clustering was performed using the Python library `scikit-learn` (version 1.0.2), with complete linkage and distance defined as $1 - S$, where S denotes the pairwise similarity between hemispheres, computed from the CC and Dice overlap (the same similarity matrix and parameters as described in the manuscript). This establishes a dendrogram which encodes the distance between different surfaces based on the similarity matrix. The resulting dendrogram was then

evaluated across a range of cluster numbers (K = 5–40). To assess stability, we repeatedly subsampled 90% of hemispheres without replacement and recomputed the clustering using identical parameters. For each subsample, cluster assignments were compared with those from the full dataset, using only the hemispheres present in both sets. Agreement was quantified using the adjusted mutual information (AMI), which accounts for chance agreement and is invariant to label permutation. This procedure was repeated 100 times for each value of K, yielding a mean and standard deviation of AMI.

Fig.S12A shows clustering consistency as a function of the number of clusters. Across lobes, clustering stability increased gradually with the number of clusters. This indicates that optimising for stability would lead to a trivial solution where each sample forms its own cluster.

To further assess over-fragmentation, we quantified the number of clusters containing very few hemispheres (<5, Fig.S12B). At larger K values, the number of such small clusters increased rapidly, particularly beyond $K \approx 25 - 30$, indicating fragmentation into low-population clusters. Taken together, these results reveal a trade-off between clustering stability and fragmentation. We therefore selected K=30 as a representative operating point that balances reproducibility and interpretability while limiting over-fragmentation.

More fundamentally, while surface registration aims to map all brains into a common coordinate system, perfect alignment of cortical folding patterns within a single diffeomorphic space is limited by topological variability (e.g. sulcal branching or interruptions), which cannot be resolved using shape information alone. Clustering therefore provides an intermediate representation that captures dominant modes of folding variation prior to alignment. In this framework, the number of clusters determines how much distinct folding variability is treated as separately alignable under diffeomorphic constraints. Increasing K improves within-cluster alignment by separating unalignable folding patterns, but at higher K begins to partition continuous variation, leading to over-fragmentation. Future work may further extend the MSM-HT framework by using functional information to guide alignment, or by modelling folding variation in a continuous latent space (e.g. Guillon et al. (10) and Gaudin et al. (11)).

S2 Supplementary Results

S2.1 Co-occurrence analysis of cortical folding patterns across lobes Another question investigated is whether individuals who share the same variant of cortical folding in one lobe are more likely to share similar patterns in other lobes. To investigate whether cortical folding motifs co-occur across different lobes, we constructed a co-occurrence matrix for each pair of lobes. Each matrix entry represented the number of hemispheres sharing the same folding motif across both lobes (shown in Fig.S4A). To assess statistical significance, 10,000 null models were generated by randomly permuting the cluster memberships of the subjects, creating a null distribution of co-occurrence expected by chance. For each observed pair, a p-value was computed based on this distribution and corrected for multiple comparisons using the False Discovery Rate (FDR) method. To quantify the strength of co-occurrence, Cohen's Kappa (κ) for each folding motif pair was computed. Pairs with $\kappa > 0.4$ were considered to reflect moderate to substantial agreement.

Significant co-occurrences were observed between folding motifs across lobes. In total, 6 pairs between frontal and parieto-occipital motifs and 18 pairs between parieto-occipital and temporal motifs reached significance ($n = 2190$ hemispheres; p-corrected < 0.05; exact per-pair statistics in Supplementary Data; Fig.S4B). However, most showed small effect sizes (frontal and parieto-occipital: $\kappa = 0.068$–0.909 (median 0.105); parieto-occipital temporal: $\kappa = 0.035$–0.149 (median 0.102)), suggesting weak overall agreement. Two frontal and parieto-occipital pairs achieved $\kappa > 0.4$ ($\kappa = 0.909$ and 0.498; FDR-corrected $p = 0.022$ for both), each linking a pair of small observed motifs (cluster sizes 5–9). Thus cross-lobe folding co-occurrence is limited overall, consistent with folding being organised predominantly within lobes, with the exception of a few rare motif combinations that co-occur strongly. Because these rest on few hemispheres, although their effect sizes are large, they would need a larger sample to confirm (see Fig.S4C).

To address the concern that co-occurrence analysis may be biased by unequal cluster sizes, we performed an additional hemisphere-level analysis that does not rely on motif labels. This analysis used only the sulcal depth and its pairwise similarities, and was therefore independent of the clustering structure. For each lobe, we computed a 2220×2220 hemisphere–hemisphere similarity matrix (the same matrices used for hierarchical clustering), which captured how similar each hemisphere's folding pattern was to every other hemisphere in the cohort. We then assessed cross-lobar coupling by correlating the upper-triangular elements of two such matrices (e.g. frontal and parieto-occipital). This correlation quantified whether the pattern of inter-individual variation in one lobe predicted the analogous pattern in another lobe. To test whether the observed correlation could arise by chance, we used a permutation-based Mantel test ($N = 5000$). In each permutation, the hemisphere indices of one lobe's similarity matrix were randomly relabelled, such that the similarity between any two specific hemispheres remained unchanged, while correspondence between hemisphere identities across two lobes was destroyed. This generated a null distribution representing cross-lobar correlations expected in the absence of any shared mechanism.

Across all lobe pairs, the Mantel correlation between hemisphere-hemisphere similarity structures was significantly greater than expected under random permutation of hemisphere identities (frontal vs. parieto-occipital: $r = 0.361$; frontal vs. temporal: $r = 0.227$; parieto-occipital vs. temporal: $r = 0.366$, all $p = 1/5001 = 2.0 \times 10^{-4}$, the minimal attainable with $5{,}000$ permutations). These results suggest weak but statistically reliable broad cross-lobar dependencies in folding patterns at the population level.

Together, these findings indicate that cross-lobar co-occurrence of folding motifs is present but generally modest, with stronger effects primarily driven by common anatomical variants. Although genetic influences on cortical surface area are region-specific (12), the development of cortical regions is highly interconnected. Structural covariance networks, complemented with structural and functional connectivity, have offered valuable insights into how different functional regions co-develop, and have found significant covariance of cortical thickness and surface area (13–15). The present study extends the focus into cortical folding patterns, revealing a higher rate of co-occurrence between parieto-occipital and temporal folding motifs. This finding is consistent with the anatomical and functional interdependence of these lobes, potentially reflecting shared developmental influences or large-scale anatomical constraints (13), although the modest effect sizes suggest that such coordination is limited. However, it is important to consider the potential methodological bias related to the number of subjects in each cluster. While

the permutation tests were designed to mitigate this by shuffling memberships while preserving cluster sizes, this inherent bias cannot be fully ruled out. In complement to this, the permutation-based Mantel tests suggest the presence of cross-lobar dependencies in folding patterns at the population level. However, the source of this coupling is not isolated: it might arise from shared developmental influences, large-scale geometric continuity, or other global factors. Further investigation is still needed to determine whether the observed inter-lobar interactions reflect biologically meaningful relationships.

S2.2 Heritability analyses of the cortical folding motifs The role of genetics on higher-order folding was assessed by comparing the cluster membership of monozygotic (MZ) and dizygotic (DZ) twins, using only the HCP dataset. A Chi-square test was conducted to assess whether there is a significant association between the twin type and their likelihood of being in the same cluster. The p-value was corrected using an FDR of 0.05 for multiple comparisons between lobes and hemispheres. The Odds Ratio (OR) was also calculated to measure the magnitude (effect size) of the association. An OR > 1 suggests that MZ twins are more likely to present in the same folding cluster than DZ twins.

In all lobar subdivisions, motifs co-occurred more frequently in MZ siblings than in DZ. The OR was consistently larger than 1, indicating that MZ twins are more likely to be in the same cluster. (values: χ^2/corrected p/OR, left frontal: 3.94/0.047/2.16; right frontal: 7.14/0.014/2.99; left parieto-occipital: 10.45/0.004/2.97; right parieto-occipital: 6.75/0.014/2.63; left temporal: 4.51/0.040/2.39; right temporal: 10.45/0.004/2.97).

S2.3 Folding-function coupling We quantified continuous structure-function coupling by computing the Spearman correlation between the upper-triangular entries of the subject-subject folding and function similarity matrices for each lobe. Folding similarity was defined as the Pearson correlation between curvature maps of each pair of subjects within the lobe, and functional similarity as the Pearson correlation between concatenated task-fMRI contrast maps for each pair of subjects within the same lobe. Statistical significance was assessed using a permutation-based Mantel test (5000 permutations), in which subject labels were randomly permuted (simultaneously shuffling rows and columns of the functional similarity matrix) to generate a null distribution of coupling values. Across lobes, the coupling coefficient ranged from $\rho = 0.068$ to 0.104 (all permutations $p < 0.001$; Table S2). Although statistically significant due to large sample size, these correlations correspond to less than $\sim 1\%$ variance explained, suggesting that folding similarity accounts for only a limited proportion of variation in functional similarity.

To complement this continuous analysis, we next assessed structure–function relationships at the level of folding-defined clusters. We compared the within- and between-cluster structural and functional similarity across subjects. Within-cluster folding similarity is substantially larger than the between-cluster similarities across all regions (frontal: Cohen's $d = 1.54$; parieto-occipital: $d = 1.67$; temporal: $d = 1.41$; Table S3), confirming that the MSM-HT clusters capture strongly coherent folding patterns. In contrast, functional similarity showed only minimal within-cluster increases (frontal: Cohen's $d = 0.054$; parieto-occipital: $d = 0.06$; temporal: $d = 0.09$; Table S3), indicating limited functional differentiation between folding motifs. This variation is not strongly structured by folding-defined clusters, as evidenced by the minimal separation between within- and between-cluster distributions.

S3 Supplementary Figures

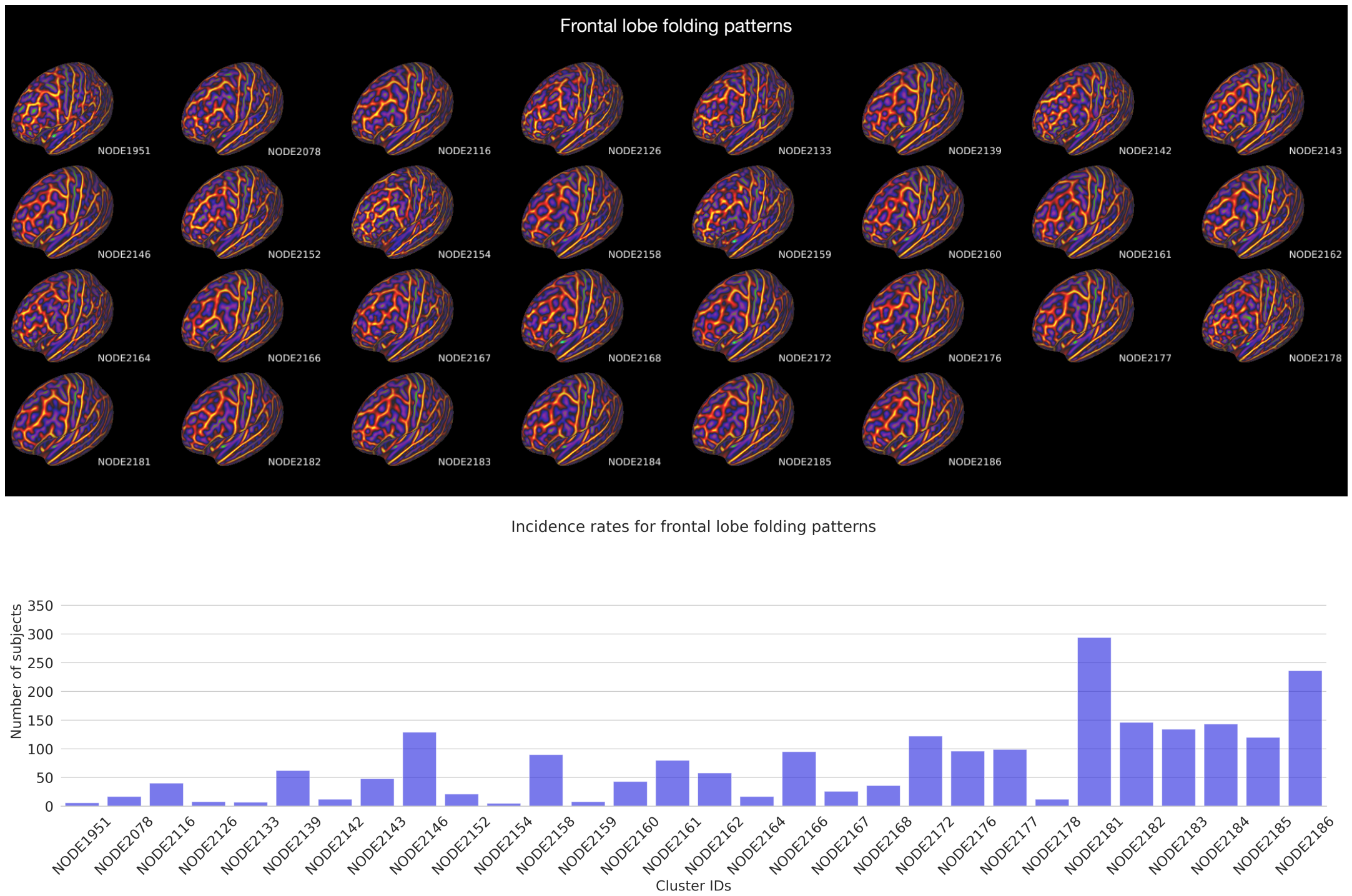

Fig. S1. Cortical folding patterns and incidence rates of the frontal lobe. The upper panel shows the thirty frontal lobe motifs; the lower panel shows the incidence rate of each motif within the HCP-YA dataset.

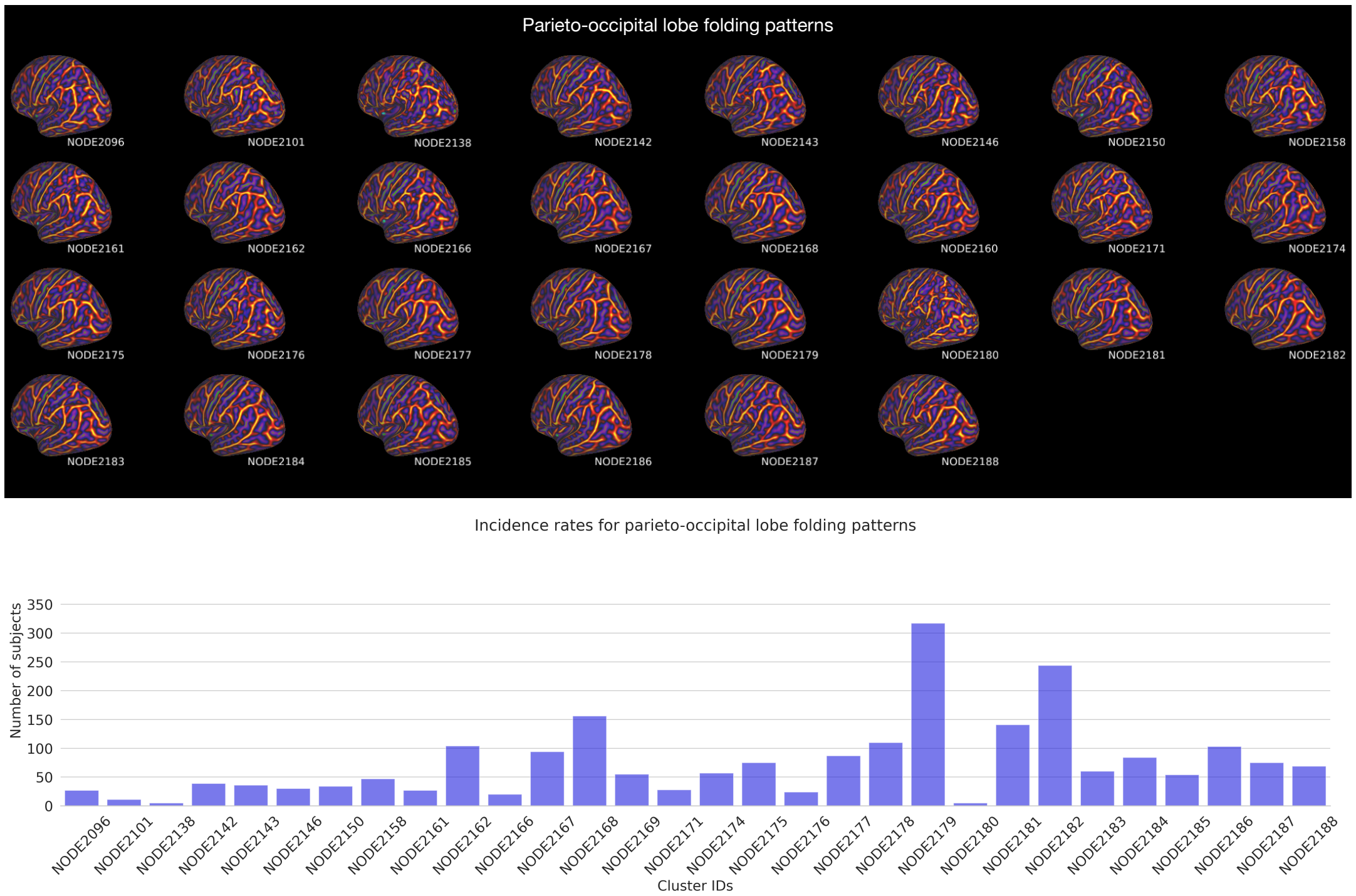

Fig. S2. Cortical folding patterns and incidence rates of the parieto-occipital lobe. The upper panel shows the thirty parieto-occipital lobe motifs; the lower panel shows the incidence rate of each motif within the HCP-YA dataset.

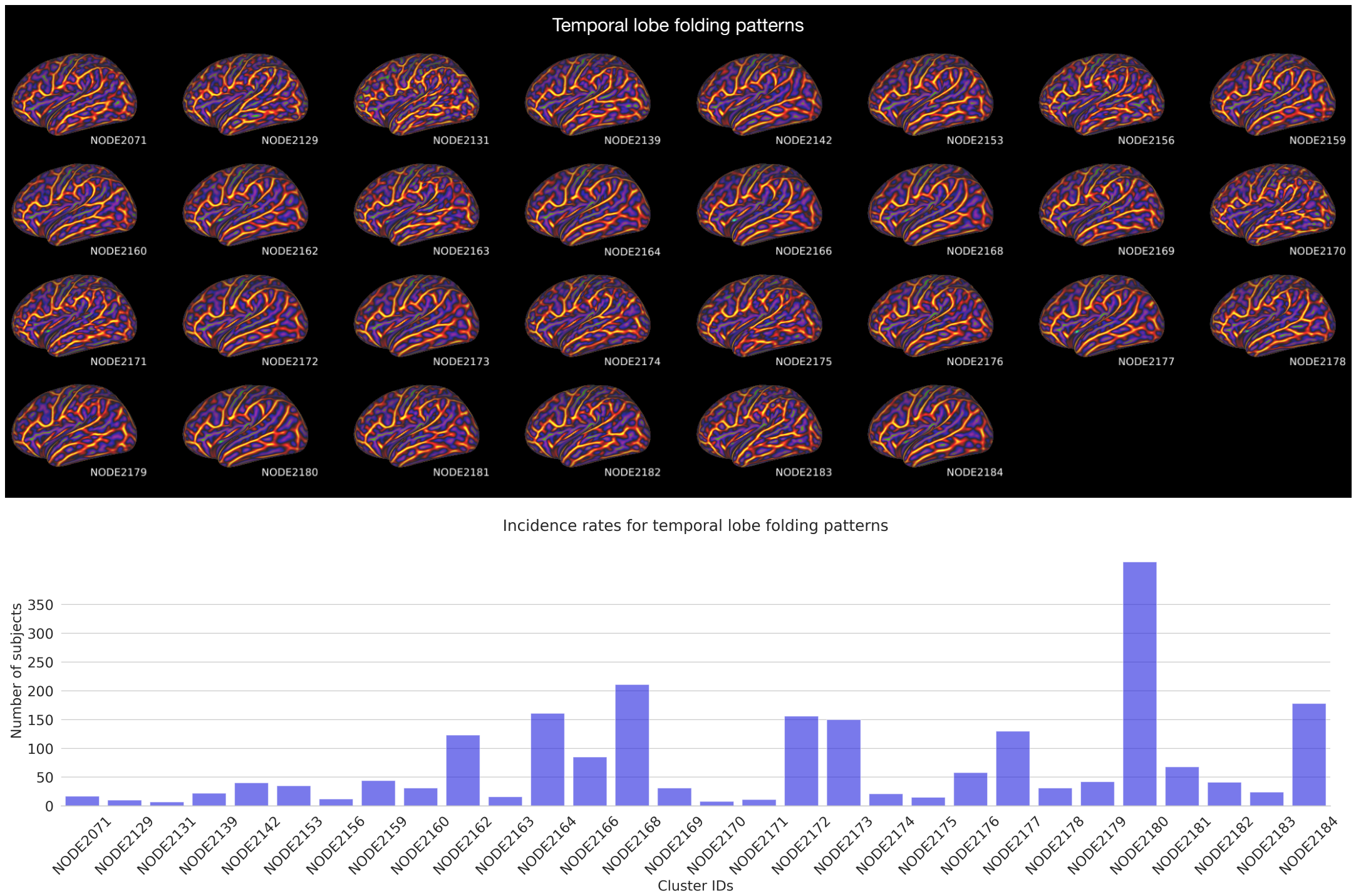

Fig. S3. Cortical folding patterns and incidence rates of the temporal lobe. The upper panel shows the thirty temporal lobe motifs; the lower panel shows the incidence rate of each motif within the HCP-YA dataset.

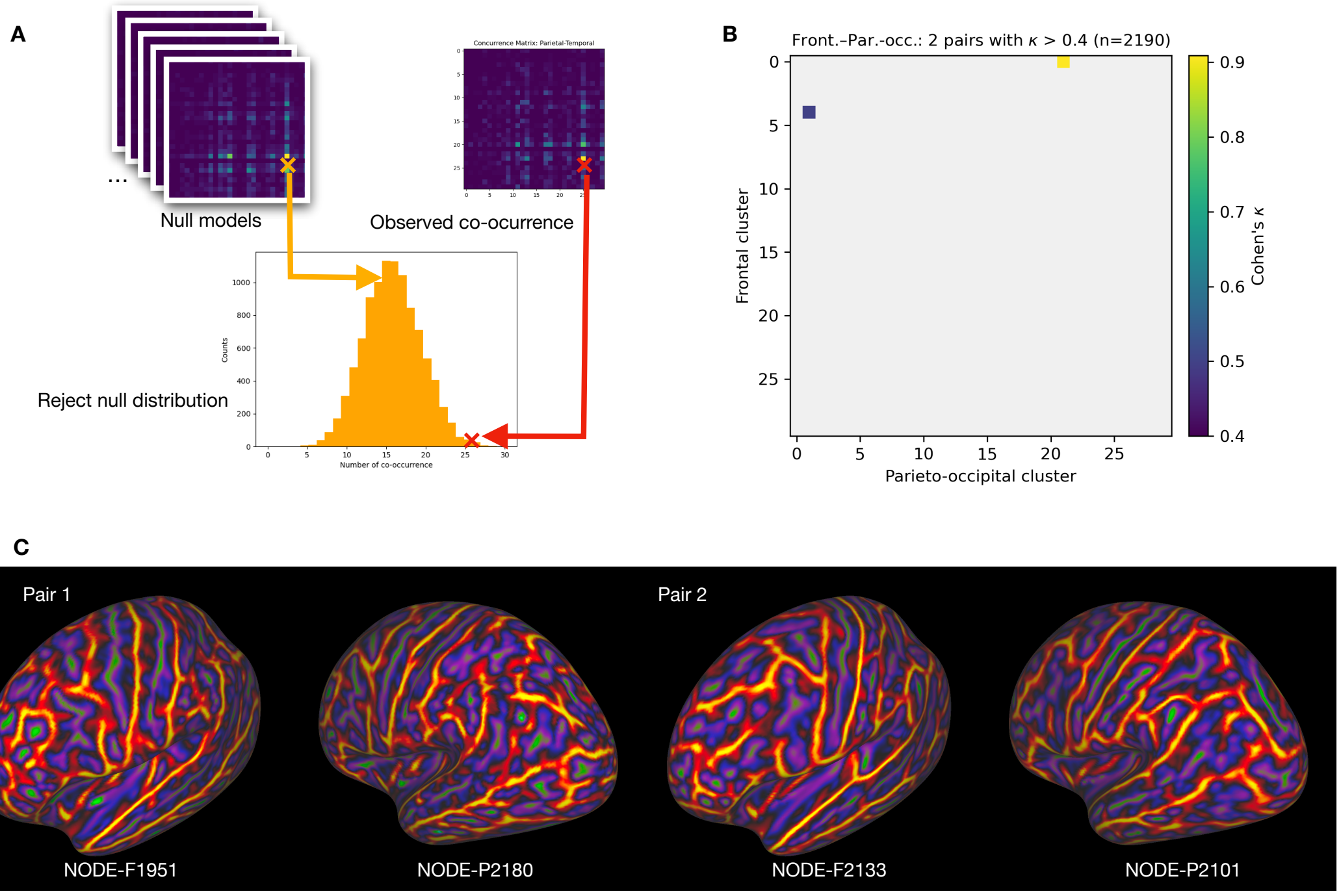

Fig. S4. Co-occurrence between lobes. **A.** Construction of null co-occurrence matrices by permutation of subjects across different clusters. 0.05 is set as a threshold to reject null models. **B.** Co-occurrence with Cohen's κ > 0.4. Colour encodes Cohen's κ (scale 0.4–0.9). **C.** The pairs of cortical folding patterns that significantly co-occur and exceed $\kappa > 0.4$. In total, 6 pairs between frontal and parieto-occipital motifs and 18 pairs between parieto-occipital and temporal motifs reached significance ($n = 2190$ hemispheres; p-corrected < 0.05; exact per-pair statistics in Supplementary Data; Fig.S4B). However, most showed small effect sizes (frontal and parieto-occipital: $\kappa = 0.068$–0.909 (median 0.105); parieto-occipital and temporal: $\kappa = 0.035$–0.149 (median 0.102)), suggesting weak overall agreement. Two pairs of co-occurrence between frontal and parieto-occipital regions achieved $\kappa > 0.4$ ($\kappa = 0.909$ and 0.498; FDR-corrected $p = 0.022$ for both), each linking a pair of small observed motifs (cluster sizes 5–9). Surfaces in C are coloured by curvature (as in Fig 1)

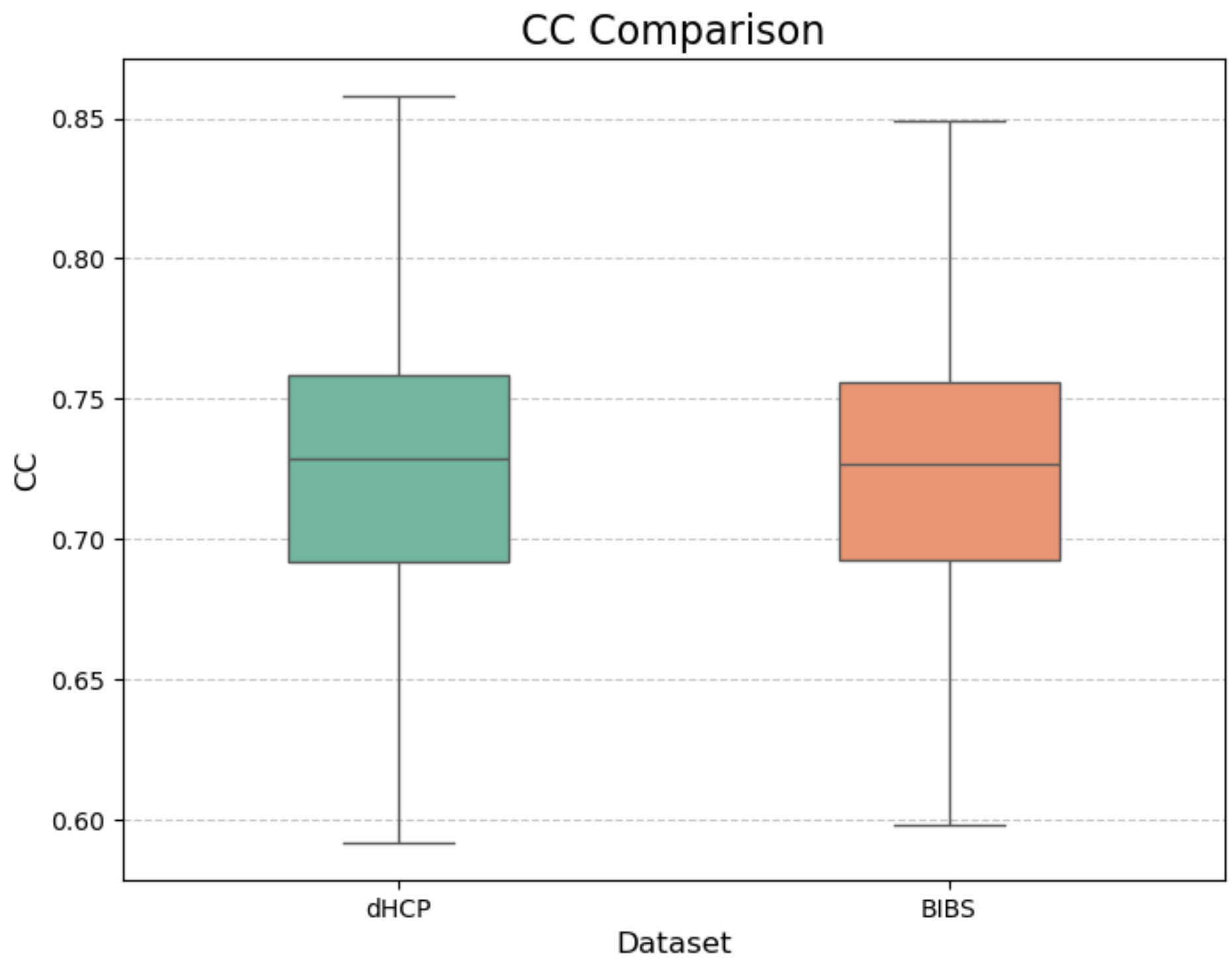

Fig. S5. Cross-correlation accuracy across training (dHCP) and testing (BIBS) datasets. Box shows the median (centre line) and 25th–75th percentiles; whiskers extend to the most extreme value within $1.5\times$ IQR (no outliers). Mean CC values are 0.717 ± 0.066 for dHCP (n = 1,027,461 hemisphere pairs) and 0.720 ± 0.054 for BIBS (n = 19,900 hemisphere pairs); $\pm$ values are SD. The effect size was negligible (Cohen's $d = -0.044$, 95% CI $[-0.058, -0.030]$); pairs are non-independent, so the CI reflects pair count rather than independent replicates. These values suggest DDR performs consistently across training and testing datasets. Source data are provided as a Supplementary Data file.

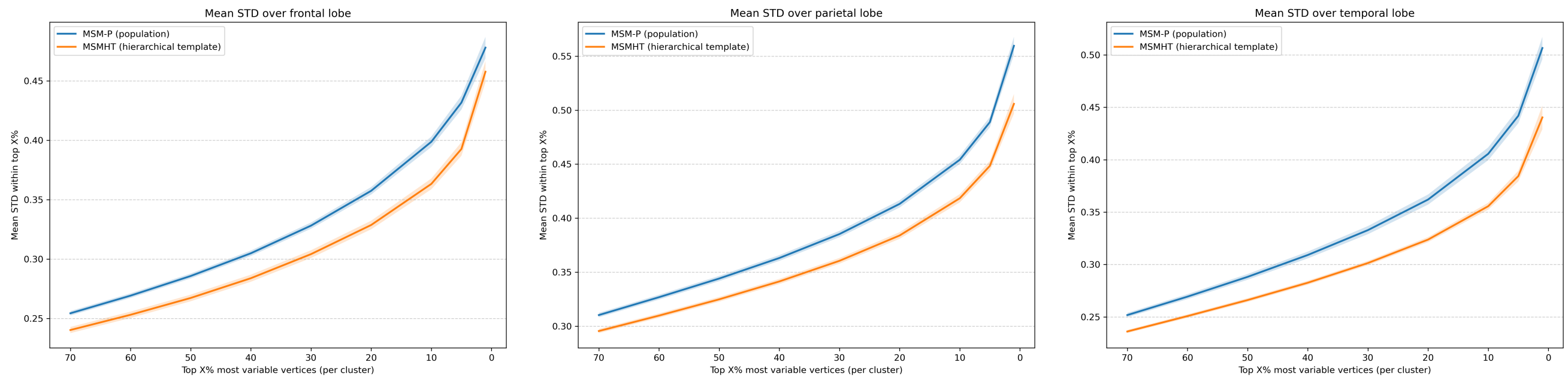

Fig. S6. Residual variability across progressively more variable cortical regions. A reference variability map was first constructed by taking the per-vertex maximum variability observed across MSM-HT and MSM-P. Vertices were then ranked according to this reference variability, and subsets corresponding to the top 70%, 60%, 50%, 40%, 30%, 20%, 10%, and 1% most variable vertices were analysed. Within each subset, the mean residual variability was calculated separately for MSM-HT and MSM-P, producing threshold–response curves describing how alignment precision changes when progressively focusing on highly variable cortical regions. Lines show the mean and shaded bands the $\pm$ standard error of the mean (SEM) across $n = 30$ clusters per lobe. Panels show the frontal (left), parieto-occipital (middle), and temporal (right) lobes. Source data are provided as a Supplementary Data file.

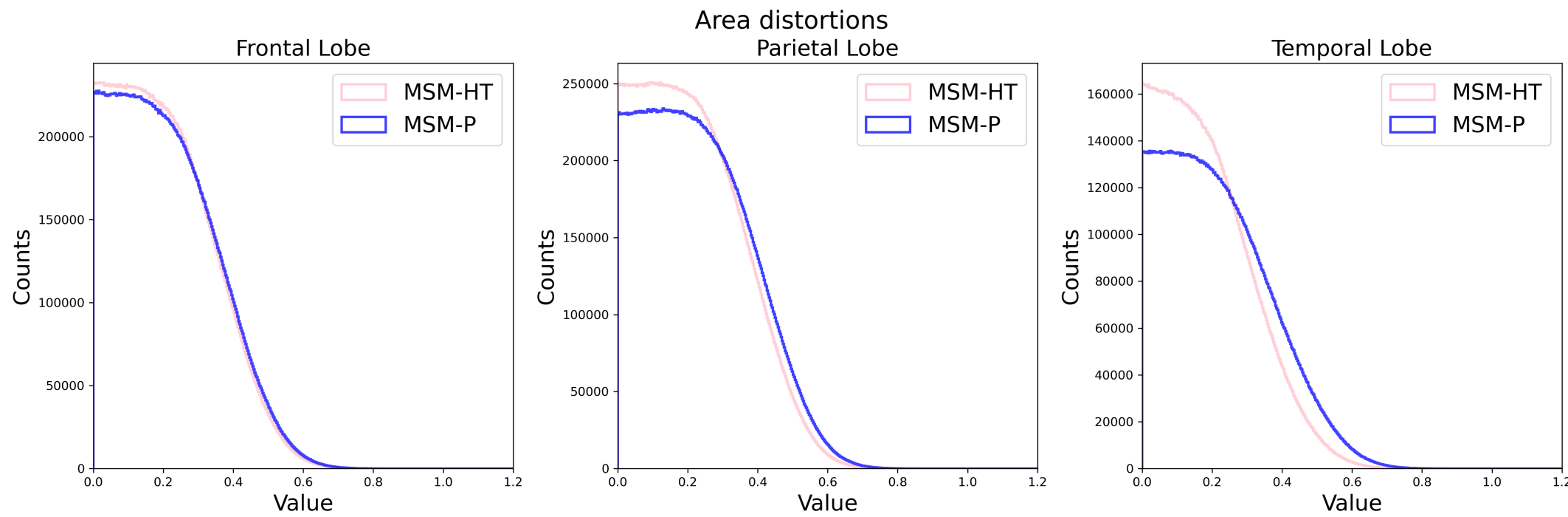

Fig. S7. Area distortion across cortical lobes. Area distortion was assessed for MSM-HT and MSM-P across the frontal, parieto-occipital, and temporal lobes ($n = 2220$ hemispheres, all vertices pooled per lobe). Panels show the frontal (left), parieto-occipital (middle), and temporal (right) lobes. Source data are provided as a Supplementary Data file.

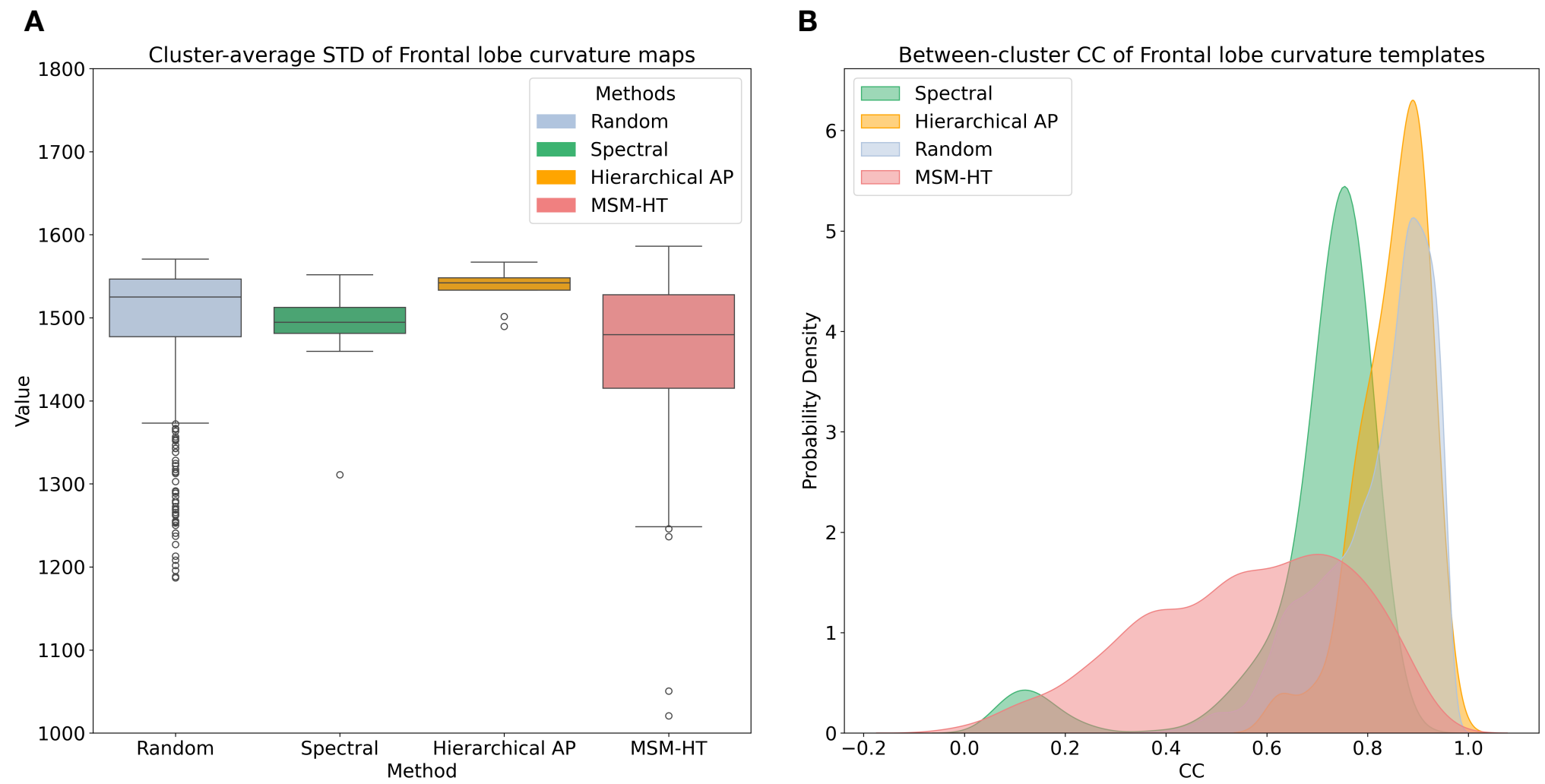

Fig. S8. Within- and between-cluster performance. **A.** Within-cluster STD and between-cluster CC were compared between four methods: Randomly generated clusters ($n = 500$ clusters), Spectral clustering (SP, $n = 30$ clusters), Hierarchical affinity propagation (hierarchical AP, $n = 9$ clusters), and MSM-HT ($n = 30$ clusters), for the frontal lobe. Boxes show the median, 25th–75th percentiles, and whiskers to $1.5\times$ IQR; outliers are shown as points. Within-cluster STD was significantly lower for MSM-HT than for each of the other three methods (one-sided two-sample t-tests against MSM-HT, Benjamini–Hochberg FDR-corrected across the three: Random, $t(528) = 3.69$, $p = 3.7 \times 10^{-4}$; SP, $t(58) = 2.03$, $p = 0.024$; Hierarchical AP, $t(37) = 2.08$, $p = 0.024$), indicating lower residual registration variability within MSM-HT clusters. **B.** Between-cluster template similarity (cross-correlation, CC) for each method; the higher, more concentrated CC distribution for MSM-HT indicates more distinct, well-separated cluster templates. Source data are provided as a Supplementary Data file.

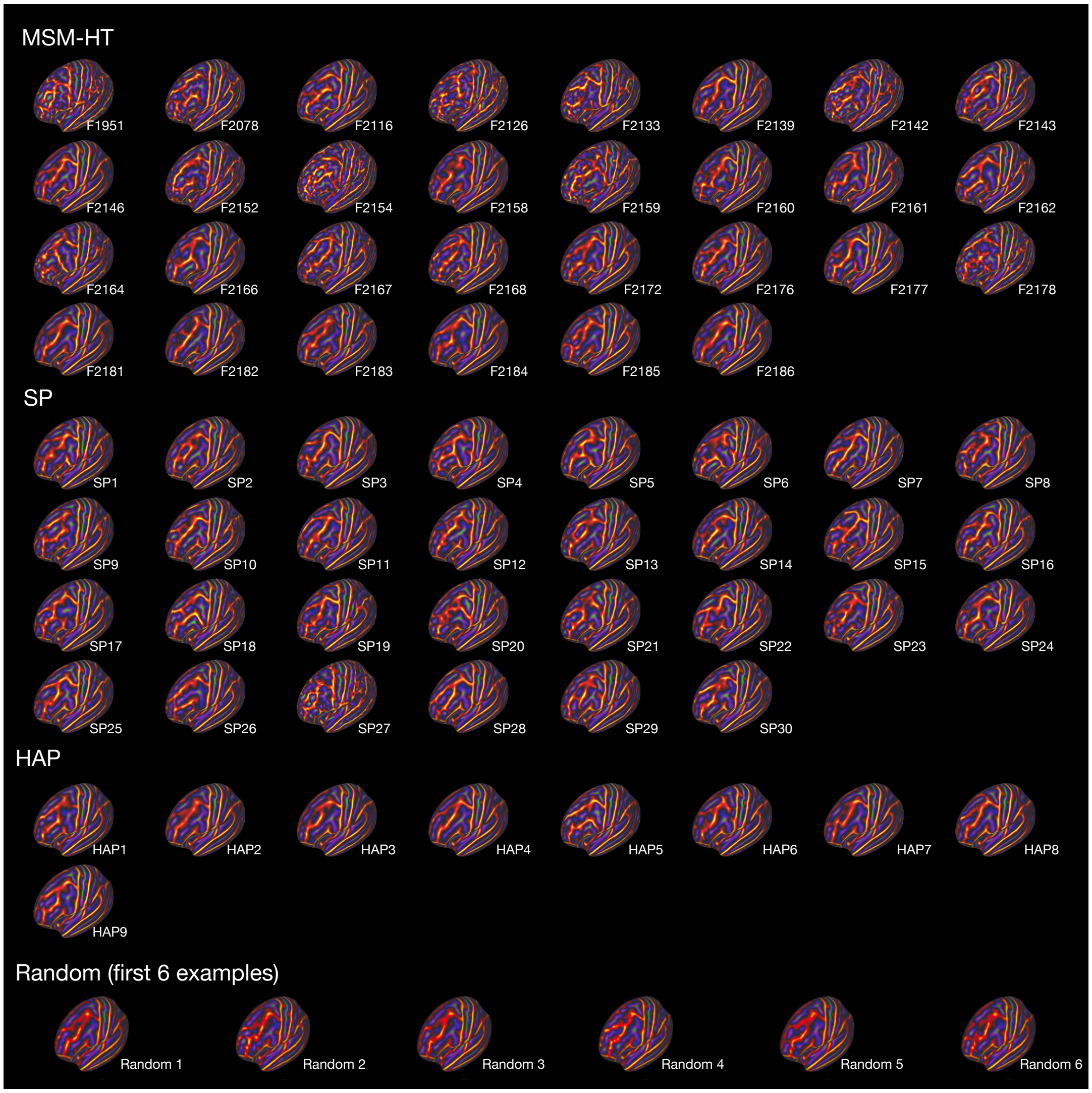

Fig. S9. Visual comparison of cortical folding templates generated by different clustering methods. Templates generated by MSM-HT, SP, hierarchical AP (HAP), and random allocation are visually compared. MSM-HT generates visually sharper mean maps with more detail and greater variability than templates generated from SP, hierarchical AP, or random allocation. For example, single-segment precentral sulcus clusters such as cluster F2126 are only uncovered by MSM-HT, but only two or three segments were found in SP and hierarchical AP clusters. In MSM-HT, most of the clusters show more detailed tertiary folds branched from the major folds, but SP clusters are more blurry around the major folds, and worse in hierarchical AP clusters. An example of this is clusters F1951 and F2142 in MSM-HT against cluster SP18 in SP clusters and cluster HAP5 in hierarchical AP clusters. Random clusters, which serve as a baseline for comparison, exhibited the least defined patterns among all methods evaluated. These clusters generally lack details of segments or branching patterns and often resemble the overall population average.

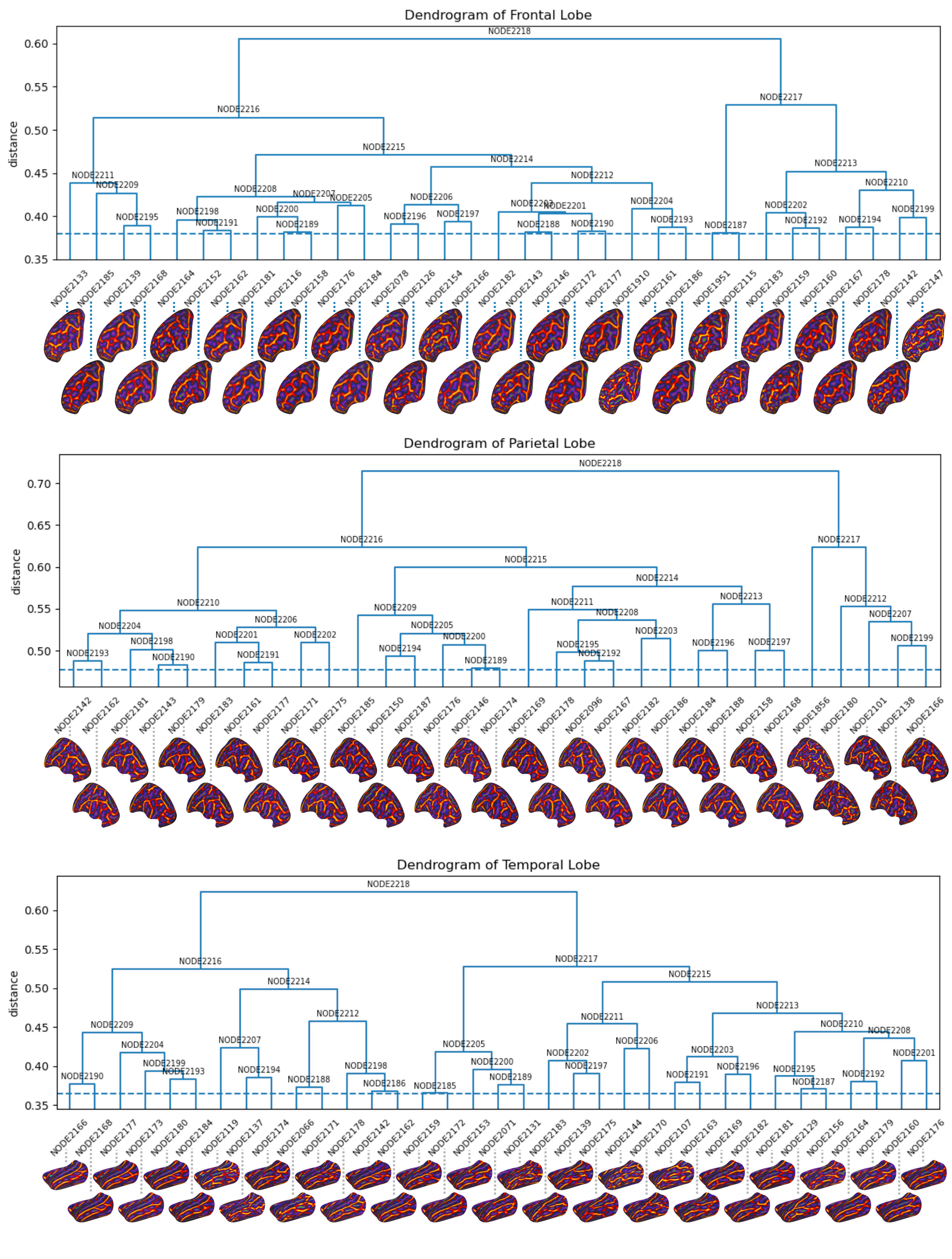

Fig. S10. Hierarchical clustering dendrogram in the HCP-YA dataset. Top panel is the frontal lobe, middle panel the parieto-occipital lobe, and the bottom panel the temporal lobe. The dendrograms are shown up to the 30-cluster level. Source data are provided as a Supplementary Data file.

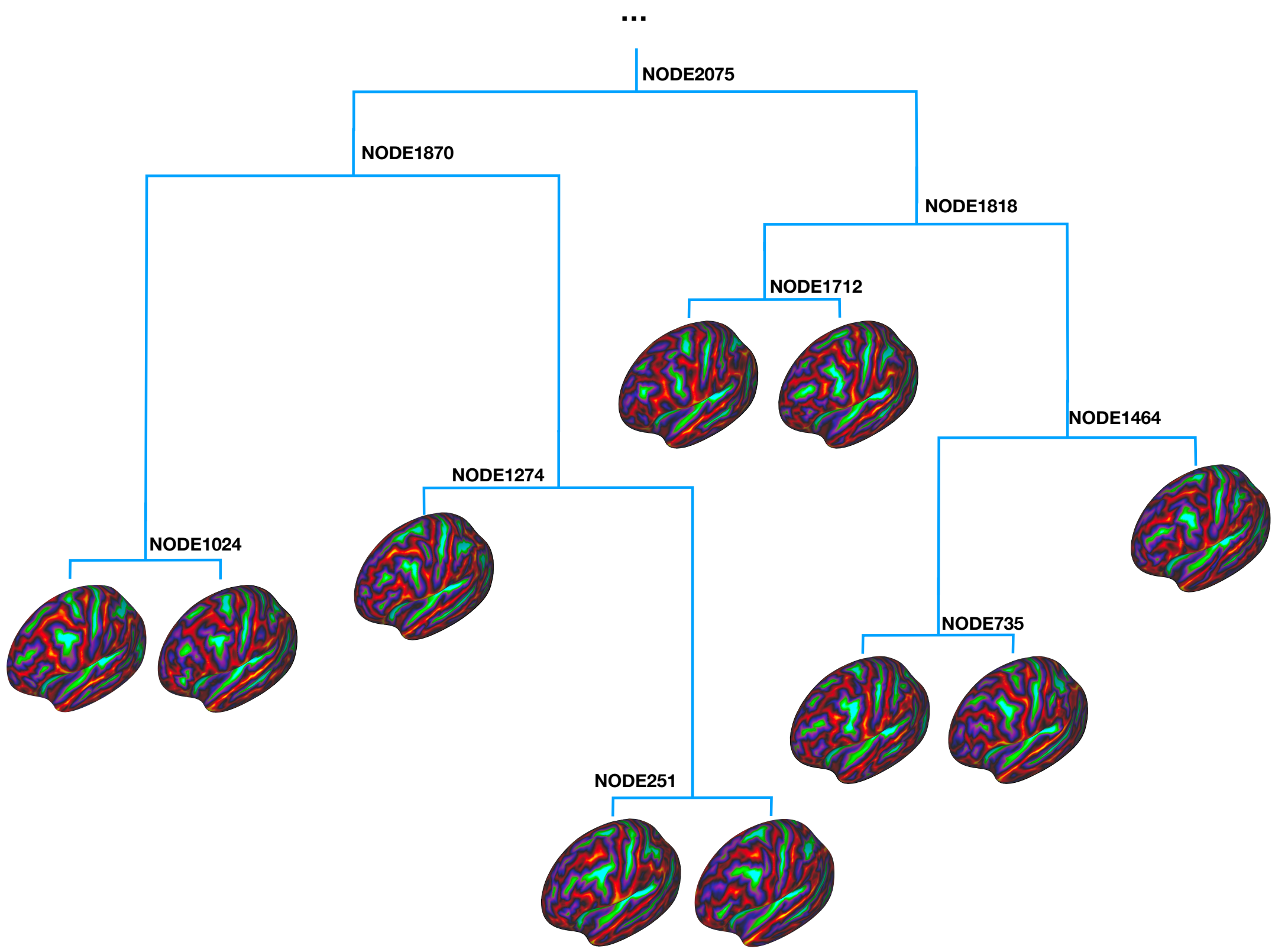

Fig. S11. Example partial subject-level dendrogram for the HCP-YA frontal lobe cluster NODE2164. For clarity, only a subset of subjects is shown, and individual subject identifiers are omitted. Source data are provided as a Supplementary Data file.

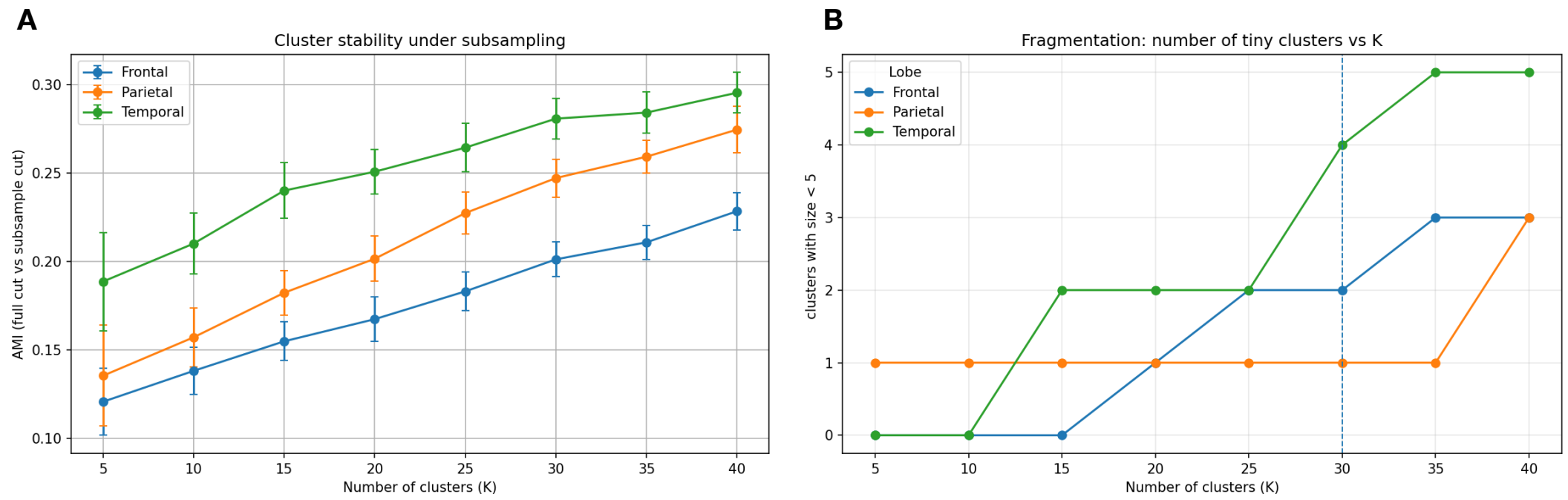

Fig. S12. Clustering consistency vs. the number of clusters (K). **A.** Cluster stability across lobes, quantified as adjusted mutual information (AMI) between the full-cohort clustering cut at K and each subsample's independent clustering at the same K. Points show mean AMI and error bars $\pm$ SD across 100 bootstrap subsamples per K (each 90% of hemispheres, sampled without replacement; $n = 2220$ hemispheres). AMI increased gradually with K; the $K = 30$–40 range used for the main analysis lies at the high-stability end. **B.** At larger K values, the number of small clusters (< 5 hemispheres) increased rapidly, indicating fragmentation into low-population clusters. Lobes: frontal, parieto-occipital, temporal. Source data are provided as a Supplementary Data file.

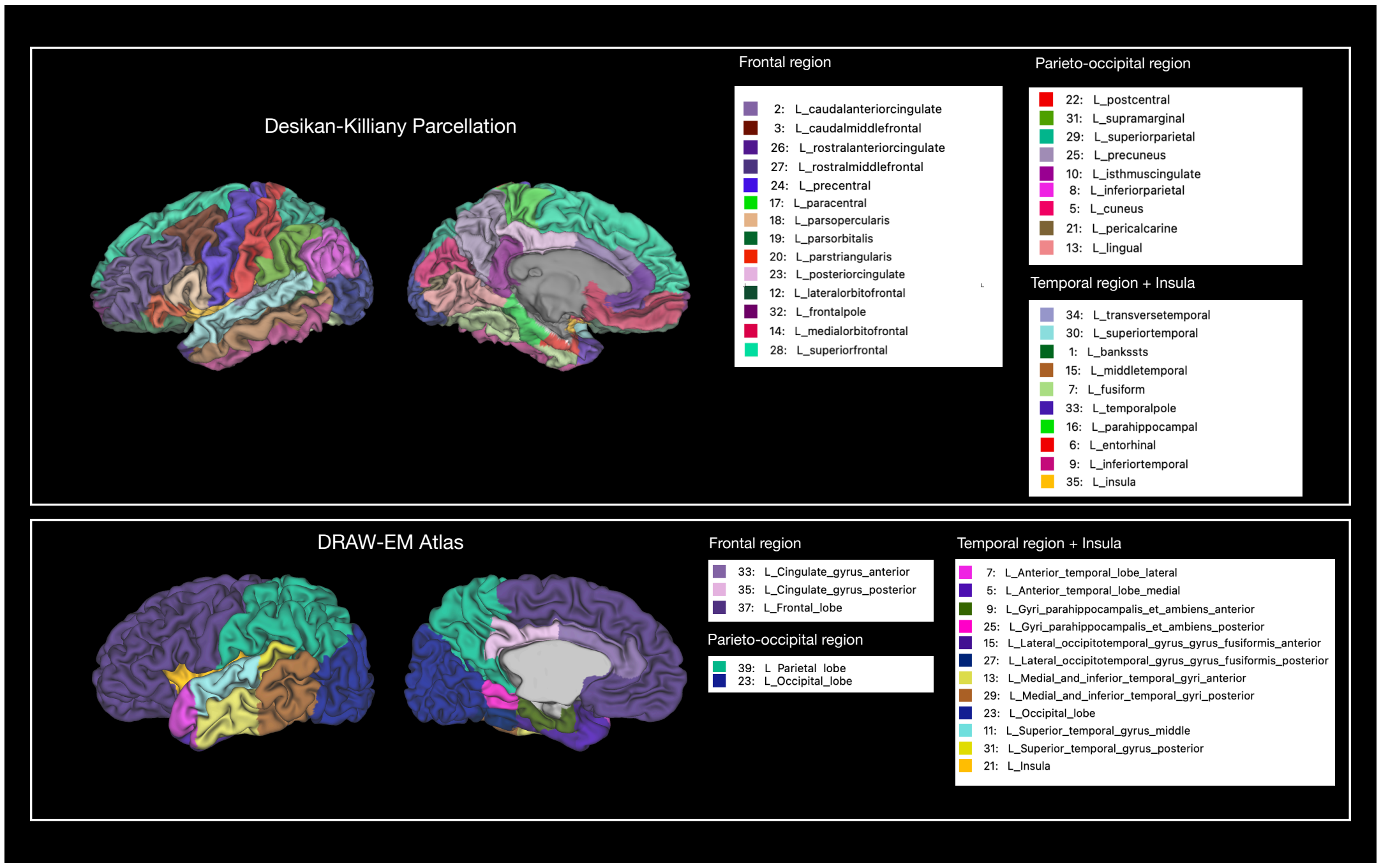

Fig. S13. Lobar regions for hierarchical clustering. Parcels included in the frontal, parieto-occipital, and temporal lobe regions are shown for adult (Desikan–Killiany) and neonatal (DRAW-EM) datasets, illustrating differences in atlas definitions across age groups. The insular cortex was grouped with the temporal lobe in the clustering analysis because sulcal depth patterns in the insula exhibited very limited inter-individual variability (mean pairwise cross-correlation $= 0.964$ in the population-average space), which did not yield stable clusters when analysed separately.

S4 Supplementary Tables

Table S1. AE model estimates of heritability (h^2) for left and right hemisphere similarity-to-template measures, and the genetic correlation between hemispheres (r_g^{LR}), across representative cortical folding clusters.

Lobe	Cluster	h^2_L	h^2_R	r_g^{LR}
**Frontal**				
Frontal	NODE2139	0.477	0.284	1.000
Frontal	NODE2146	0.533	0.344	1.000
Frontal	NODE2158	0.487	0.358	1.000
Frontal	NODE2161	0.468	0.305	1.000
Frontal	NODE2162	0.342	0.257	1.000
Frontal	NODE2166	0.447	0.288	1.000
Frontal	NODE2172	0.487	0.338	1.000
Frontal	NODE2176	0.508	0.383	1.000
Frontal	NODE2177	0.472	0.330	1.000
Frontal	NODE2181	0.496	0.316	1.000
Frontal	NODE2182	0.534	0.354	1.000
Frontal	NODE2183	0.473	0.331	1.000
Frontal	NODE2184	0.441	0.296	1.000
Frontal	NODE2185	0.532	0.353	1.000
Frontal	NODE2186	0.529	0.348	1.000
**Parieto-occipital**				
Parieto-occipital	NODE2162	0.323	0.480	0.895
Parieto-occipital	NODE2167	0.428	0.365	0.910
Parieto-occipital	NODE2168	0.315	0.452	0.902
Parieto-occipital	NODE2169	0.207	0.375	1.000
Parieto-occipital	NODE2174	0.504	0.444	0.778
Parieto-occipital	NODE2175	0.486	0.497	0.835
Parieto-occipital	NODE2177	0.341	0.452	0.961
Parieto-occipital	NODE2178	0.286	0.385	0.875
Parieto-occipital	NODE2179	0.393	0.463	0.835
Parieto-occipital	NODE2181	0.312	0.438	0.828
Parieto-occipital	NODE2182	0.480	0.483	0.850
Parieto-occipital	NODE2183	0.281	0.396	0.953
Parieto-occipital	NODE2184	0.223	0.364	0.908
Parieto-occipital	NODE2185	0.461	0.350	0.999
Parieto-occipital	NODE2186	0.283	0.341	0.969
Parieto-occipital	NODE2187	0.336	0.437	0.824
Parieto-occipital	NODE2188	0.261	0.404	0.933
**Temporal**				
Temporal	NODE2162	0.465	0.402	0.610
Temporal	NODE2164	0.539	0.494	0.549
Temporal	NODE2166	0.454	0.398	0.719
Temporal	NODE2168	0.492	0.430	0.669
Temporal	NODE2172	0.530	0.457	0.574
Temporal	NODE2173	0.534	0.392	0.647
Temporal	NODE2176	0.448	0.426	0.479
Temporal	NODE2177	0.467	0.489	0.619

Lobe	Cluster	h_L^2	h_R^2	r_g^{LR}
Temporal	NODE2180	0.530	0.461	0.645
Temporal	NODE2181	0.485	0.522	0.434
Temporal	NODE2184	0.502	0.428	0.606

Table S2. Coupling coefficient of structure and function. Structure-function coupling in each lobe was quantified as the Spearman rank correlation ρ between structural (folding) similarity and functional-activation similarity across all subject pairs (upper-triangular entries of the subject $\times$ subject similarity matrices). Significance was assessed with a two-sided Mantel permutation test ($5{,}000$ permutations); p-values were Benjamini–Hochberg-corrected across the three lobes. For every lobe no permuted statistic reached the observed value, so each p sits at the permutation floor, $1/(5{,}000+1) = 2\times10^{-4}$. The 95% confidence interval for ρ is from a subject-level (leave-one-subject-out) jackknife; $R^2 = 100\times\rho^2$.

Lobe	ρ (Spearman)	95% CI	R^2 (%)	p (Mantel)
Frontal	0.094	$[0.067, 0.121]$	0.89	2×10^{-4}
Parieto-occipital	0.104	$[0.077, 0.131]$	1.08	2×10^{-4}
Temporal	0.068	$[0.038, 0.097]$	0.46	2×10^{-4}

Table S3. Within- and between-cluster similarity for folding and function across lobes

	Folding			Function		
Lobe	Within	Between	Cohen's d	Within	Between	Cohen's d
Frontal	0.494	0.415	1.54	0.227	0.224	0.054
Parieto-occipital	0.461	0.378	1.67	0.388	0.384	0.060
Temporal	0.539	0.454	1.41	0.302	0.295	0.091

Table S4. Descriptive statistics for each motif. Curv$^+$ and Curv$^-$ denote mean positive and negative curvature, respectively. SD$^+$ and SD$^-$ denote mean positive and negative sulcal depth. AI denotes asymmetry index.

Lobe	Motif ID	Size	Curv$^+$	Curv$^-$	SD$^+$	SD$^-$	Area	AI
Frontal	NODE1951	6	0.150	-0.108	0.393	-0.458	37918.656	-0.333
Frontal	NODE2078	17	0.153	-0.108	0.394	-0.477	36902.441	-0.412
Frontal	NODE2116	40	0.152	-0.108	0.388	-0.472	34216.727	0.700
Frontal	NODE2126	8	0.153	-0.109	0.395	-0.469	36193.789	-1.000
Frontal	NODE2133	7	0.154	-0.108	0.394	-0.462	33781.459	0.714
Frontal	NODE2139	62	0.152	-0.108	0.391	-0.472	33417.712	0.226
Frontal	NODE2142	12	0.153	-0.108	0.386	-0.472	40277.335	-0.333
Frontal	NODE2143	48	0.152	-0.106	0.391	-0.478	35457.976	0.792
Frontal	NODE2146	129	0.152	-0.108	0.392	-0.474	33466.495	0.364
Frontal	NODE2152	21	0.155	-0.109	0.382	-0.471	36577.052	0.048
Frontal	NODE2154	5	0.155	-0.108	0.377	-0.464	39328.895	0.200
Frontal	NODE2158	90	0.153	-0.108	0.390	-0.472	34895.440	0.422
Frontal	NODE2159	8	0.155	-0.109	0.393	-0.465	34639.381	-0.250
Frontal	NODE2160	43	0.153	-0.109	0.390	-0.471	36284.392	-0.581
Frontal	NODE2161	80	0.152	-0.109	0.390	-0.467	33526.301	-0.375
Frontal	NODE2162	58	0.153	-0.108	0.391	-0.469	36037.588	-0.414
Frontal	NODE2164	17	0.153	-0.109	0.391	-0.469	36336.740	0.412
Frontal	NODE2166	95	0.153	-0.108	0.388	-0.471	36537.589	0.284
Frontal	NODE2167	26	0.154	-0.110	0.384	-0.465	37352.981	-0.231
Frontal	NODE2168	36	0.152	-0.108	0.389	-0.464	33232.439	-0.722
Frontal	NODE2172	122	0.153	-0.109	0.392	-0.464	33967.406	-0.557
Frontal	NODE2176	96	0.153	-0.108	0.390	-0.472	35674.152	0.188
Frontal	NODE2177	99	0.153	-0.108	0.391	-0.470	34806.530	-0.232
Frontal	NODE2178	12	0.150	-0.108	0.387	-0.468	39300.119	-0.167
Frontal	NODE2181	294	0.152	-0.108	0.391	-0.474	34310.359	0.184
Frontal	NODE2182	146	0.152	-0.108	0.391	-0.472	34499.602	-0.205
Frontal	NODE2183	134	0.153	-0.109	0.390	-0.473	36141.168	0.269
Frontal	NODE2184	143	0.152	-0.108	0.390	-0.470	35104.672	0.035
Frontal	NODE2185	120	0.153	-0.108	0.391	-0.467	33497.302	-0.150
Frontal	NODE2186	236	0.153	-0.108	0.389	-0.468	33471.926	-0.186
Parieto-occipital	NODE2096	27	0.159	-0.109	0.407	-0.509	35502.956	-0.926
Parieto-occipital	NODE2101	11	0.160	-0.110	0.403	-0.508	35202.931	0.273
Parieto-occipital	NODE2138	5	0.158	-0.111	0.411	-0.520	33812.546	-0.600
Parieto-occipital	NODE2142	39	0.159	-0.108	0.409	-0.511	35442.113	0.385
Parieto-occipital	NODE2143	36	0.160	-0.109	0.410	-0.505	34654.012	-0.222
Parieto-occipital	NODE2146	30	0.159	-0.110	0.407	-0.506	36457.110	-0.867
Parieto-occipital	NODE2150	34	0.158	-0.110	0.410	-0.509	36426.193	-0.353
Parieto-occipital	NODE2158	47	0.159	-0.109	0.408	-0.514	36139.296	-0.064
Parieto-occipital	NODE2161	27	0.160	-0.108	0.406	-0.508	35452.486	-0.259
Parieto-occipital	NODE2162	104	0.159	-0.109	0.408	-0.507	34917.016	0.519
Parieto-occipital	NODE2166	20	0.161	-0.110	0.411	-0.514	33273.503	-0.300
Parieto-occipital	NODE2167	94	0.159	-0.109	0.409	-0.509	36139.636	-0.596
Parieto-occipital	NODE2168	156	0.158	-0.109	0.407	-0.506	34523.326	0.500

Lobe	Motif ID	Size	Curv^{+}	Curv^{-}	SD^{+}	SD^{-}	Area	AI
Parieto-occipital	NODE2169	55	0.159	-0.109	0.408	-0.504	36863.658	0.091
Parieto-occipital	NODE2171	28	0.160	-0.109	0.405	-0.505	35663.340	0.571
Parieto-occipital	NODE2174	57	0.158	-0.108	0.412	-0.514	35477.042	-0.684
Parieto-occipital	NODE2175	75	0.160	-0.110	0.408	-0.506	34185.237	0.493
Parieto-occipital	NODE2176	24	0.158	-0.108	0.412	-0.519	38151.767	-0.667
Parieto-occipital	NODE2177	87	0.160	-0.109	0.409	-0.508	34908.263	0.034
Parieto-occipital	NODE2178	110	0.158	-0.108	0.409	-0.510	36291.744	-0.400
Parieto-occipital	NODE2179	317	0.159	-0.109	0.408	-0.509	34818.465	0.451
Parieto-occipital	NODE2180	5	0.158	-0.112	0.405	-0.506	35650.664	-0.200
Parieto-occipital	NODE2181	141	0.159	-0.109	0.406	-0.507	35196.182	0.390
Parieto-occipital	NODE2182	244	0.159	-0.109	0.409	-0.511	34352.975	-0.418
Parieto-occipital	NODE2183	60	0.159	-0.110	0.409	-0.502	34921.427	0.467
Parieto-occipital	NODE2184	84	0.158	-0.109	0.410	-0.506	36194.021	-0.357
Parieto-occipital	NODE2185	54	0.159	-0.109	0.413	-0.515	36213.634	-0.667
Parieto-occipital	NODE2186	103	0.158	-0.109	0.412	-0.511	36513.442	-0.689
Parieto-occipital	NODE2187	75	0.158	-0.109	0.410	-0.512	36909.589	-0.093
Parieto-occipital	NODE2188	69	0.159	-0.109	0.405	-0.503	35557.953	0.797
Temporal	NODE2071	17	0.147	-0.098	0.385	-0.541	20841.019	1.000
Temporal	NODE2129	10	0.146	-0.099	0.366	-0.519	21081.184	1.000
Temporal	NODE2131	7	0.145	-0.101	0.390	-0.540	21197.046	0.143
Temporal	NODE2139	22	0.149	-0.100	0.382	-0.535	20905.679	1.000
Temporal	NODE2142	40	0.145	-0.098	0.394	-0.525	20706.321	-0.650
Temporal	NODE2153	35	0.149	-0.100	0.392	-0.547	19265.919	0.886
Temporal	NODE2156	12	0.147	-0.100	0.387	-0.535	19778.717	0.833
Temporal	NODE2159	44	0.146	-0.098	0.396	-0.540	20489.587	0.409
Temporal	NODE2160	31	0.148	-0.099	0.387	-0.528	20269.169	0.355
Temporal	NODE2162	123	0.146	-0.098	0.391	-0.525	20807.521	-0.593
Temporal	NODE2163	16	0.148	-0.100	0.381	-0.535	20461.406	1.000
Temporal	NODE2164	161	0.147	-0.099	0.391	-0.542	19570.467	0.789
Temporal	NODE2166	85	0.146	-0.097	0.392	-0.521	20013.846	-0.812
Temporal	NODE2168	211	0.145	-0.098	0.393	-0.525	20081.262	-0.526
Temporal	NODE2169	31	0.146	-0.099	0.390	-0.530	20575.338	0.548
Temporal	NODE2170	8	0.151	-0.099	0.380	-0.538	20956.388	1.000
Temporal	NODE2171	11	0.146	-0.098	0.387	-0.519	21761.020	-0.273
Temporal	NODE2172	156	0.147	-0.099	0.390	-0.535	19589.283	0.346
Temporal	NODE2173	150	0.146	-0.098	0.393	-0.533	19754.839	0.053
Temporal	NODE2174	21	0.147	-0.099	0.379	-0.526	21338.497	0.810
Temporal	NODE2175	15	0.151	-0.101	0.376	-0.531	20404.051	1.000
Temporal	NODE2176	58	0.146	-0.099	0.388	-0.534	21233.078	0.586
Temporal	NODE2177	130	0.146	-0.098	0.394	-0.531	19766.891	-0.308
Temporal	NODE2178	31	0.146	-0.098	0.388	-0.513	21981.212	-0.935
Temporal	NODE2179	42	0.150	-0.100	0.387	-0.543	19500.004	1.000
Temporal	NODE2180	424	0.146	-0.098	0.394	-0.532	19529.270	-0.481
Temporal	NODE2181	68	0.148	-0.099	0.390	-0.540	20363.395	0.765

Lobe	Motif ID	Size	Curv$^+$	Curv$^-$	SD$^+$	SD$^-$	Area	AI
Temporal	NODE2182	41	0.147	-0.100	0.396	-0.546	21558.057	1.000
Temporal	NODE2183	24	0.146	-0.100	0.378	-0.531	21533.460	1.000
Temporal	NODE2184	178	0.147	-0.098	0.395	-0.529	19641.251	-0.191

Table S5. Cluster mass comparison between MSM-HT and MSM-P across tasks and lobes. Paired two-tailed t-tests; p-values FDR-corrected (Benjamini–Hochberg) across the three lobes within each task. The relative difference is $100 \times (\overline{M}_{\text{P}} - \overline{M}_{\text{HT}})/\overline{M}_{\text{P}}$, so positive values indicate lower total cluster mass under MSM-HT; 95% confidence intervals are from a paired cluster-level bootstrap (10,000 resamples).

Task	Lobe	t (df)	Corrected p	Rel. diff. (%)	95% CI (%)
Language	Frontal	9.62 (57)	4.6×10^{-13}	4.25	$[3.67, 4.87]$
	Parieto-occipital	7.34 (57)	1.3×10^{-9}	2.18	$[1.77, 2.54]$
	Temporal	2.08 (46)	0.043	1.02	$[0.08, 1.85]$
WM	Frontal	7.10 (56)	4.7×10^{-9}	11.55	$[9.16, 13.78]$
	Parieto-occipital	7.05 (55)	4.7×10^{-9}	8.97	$[6.86, 10.95]$
	Temporal	-2.96 (44)	5.0×10^{-3}	-3.17	$[-5.18, -1.06]$
Motor	Frontal	6.54 (57)	2.7×10^{-8}	8.31	$[6.25, 10.34]$
	Parieto-occipital	7.15 (57)	5.4×10^{-9}	9.40	$[7.71, 10.91]$
	Temporal	4.14 (46)	1.4×10^{-4}	9.99	$[5.85, 13.99]$
Social	Frontal	2.50 (49)	0.016	3.76	$[0.82, 6.48]$
	Parieto-occipital	6.75 (52)	3.7×10^{-8}	10.62	$[8.17, 12.80]$
	Temporal	3.27 (38)	3.4×10^{-3}	5.28	$[2.38, 8.11]$

S5 References

1. Ma, Q. et al. The developing Human Connectome Project: a fast deep learning-based pipeline for neonatal cortical surface reconstruction. *Med. Image Anal.* **100**, 103394 (2025).
2. Tustison, N. J. et al. N4ITK: improved N3 bias correction. *IEEE Trans. Med. Imaging* **29**, 1310–1320 (2010).
3. Schuh, A. et al. Unbiased construction of a temporally consistent morphological atlas of neonatal brain development. Preprint at bioRxiv https://doi.org/10.1101/251512 (2018).
4. Fischl, B., Sereno, M. I. & Dale, A. M. Cortical surface-based analysis: II: inflation, flattening, and a surface-based coordinate system. *Neuroimage* **9**, 195–207 (1999).
5. Fischl, B. FreeSurfer. *Neuroimage* **62**, 774–781 (2012).
6. Makropoulos, A. et al. Automatic whole brain MRI segmentation of the developing neonatal brain. *IEEE Trans. Med. Imaging* **33**, 1818–1831 (2014).
7. Makropoulos, A. et al. The developing Human Connectome Project: a minimal processing pipeline for neonatal cortical surface reconstruction. *Neuroimage* **173**, 88–112 (2018).
8. Meng, Y. et al. Discovering cortical sulcal folding patterns in neonates using large-scale dataset. *Hum. Brain Mapp.* **39**, 3625–3635 (2018).
9. Duan, D. et al. Exploring folding patterns of infant cerebral cortex based on multi-view curvature features: methods and applications. *Neuroimage* **185**, 575–592 (2019).
10. Guillon, L. et al. Identification of rare cortical folding patterns using unsupervised deep learning. *Imaging Neurosci.* **2**, 1–27 (2024).
11. Gaudin, A. et al. Optimizing contrastive learning for cortical folding pattern detection. Preprint at https://arxiv.org/abs/2401.18035 (2024).
12. Chen, C.-H. et al. Hierarchical genetic organization of human cortical surface area. *Science* **335**, 1634–1636 (2012).
13. Lerch, J. P. et al. Mapping anatomical correlations across cerebral cortex (MACACC) using cortical thickness from MRI. *Neuroimage* **31**, 993–1003 (2006).
14. Raznahan, A. et al. Patterns of coordinated anatomical change in human cortical development: a longitudinal neuroimaging study of maturational coupling. *Neuron* **72**, 873–884 (2011).
15. Alexander-Bloch, A., Raznahan, A., Bullmore, E. & Giedd, J. The convergence of maturational change and structural covariance in human cortical networks. *J. Neurosci.* **33**, 2889–2899 (2013).
