# Supplementary material for: Motifs of brain cortical folding from birth to adulthood
Source: Nat Commun. 2026 Sep 3;17:10482. doi: 10.1038/s41467-026-77314-5 (PMC13635187; doi:10.1038/s41467-026-77314-5)
Supplement: Supplementary file 4 [file 41467_2026_77314_MOESM2_ESM.pdf]

Description of Additional Supplementary Files

File name: Supplementary Data 1
Description: Statistical source data used for Supplementary Figure S4.

File name: Supplementary Data 2
Description: Statistical source data used for Supplementary Figure S5.

File name: Supplementary Data 3
Description: Statistical source data used for Supplementary Figure S6.

File name: Supplementary Data 4
Description: Statistical source data used for Supplementary Figure S7.

File name: Supplementary Data 5
Description: Statistical source data used for Supplementary Figure S8 A and B.

File name: Supplementary Data 6
Description: Statistical source data used for Supplementary Figure S10-11.

File name: Supplementary Data 7
Description: Statistical source data used for Supplementary Figure S12.
